# Supplementary material for: Novofumigatonin biosynthesis involves a non-heme iron-dependent endoperoxide isomerase for orthoester formation
Source: Nat Commun. 2018 Jul 3;9:2587. doi: 10.1038/s41467-018-04983-2 (PMC6030086; doi:10.1038/s41467-018-04983-2)
Supplement: Supplementary file 1 — Supplementary Information [file 41467_2018_4983_MOESM1_ESM.pdf]

*Supplementary Information for*

# Novofumigatonin biosynthesis involves a non-heme iron-dependent endoperoxide isomerase for orthoester formation

**Matsuda *et al.***

## Table of Contents

| | |
|---|---|
| **Supplementary Figures 1-100** | 2-55 |
| **Supplementary Tables 1-22** | 56-75 |
| **Supplementary Notes 1-18** | 76-84 |
| **Supplementary References** | 85 |

# Supplementary Figures

## a

ATGGGCAGAGATCAGGTATCTCACAAGCGAAGCCAGAACTCCAATGTCTCTGAGATACCGGATCTAGACTCCAGTCTG
CACAGAAGTGAAGATACTCTTGTCGCAGAGATTATCGAGGCCATGACCTTAGCTGGGGTCTGCGTTGTGAGGAACTTA
TTCACTAAGTCCCTGGTTGATCAGGTACTCAAGGATTTCGAGCCTCACGTGTCGTCCACTAAACTATTTGACGGTATGC
CATACAATGCTCTATTCATGGTCAGCTTTATATTATTCACCCCTTAATTGCATGTTAATTCTCCTGATCACCAATAATTACTG
TTCGAGCAATAACGATATGGTGCTGTGCAGGCTACCCCGGCAATGGCTGCCATCTGACAGGATTGCTCAGCAAATCGG
AGATCTATGCCCATATGGTTGTGGGAAACAGCGTGTTTGAGAAAGTTCGGAACCACTTTCTCAGTACCACCTTTCGGTC
ATGGATAGGTGGGAAGATGATGACATTCACCAGTCCCCCCCAGCTCGACTCTACTATCTGCTCCTACATCAACCCACAG
TCACCCGGTGAGCATCTACACCGCGATGACGCGATTCATTACGGCTGGAATGAGGCAGCGAGTGAGTACACAGTCGG
CCGAGACATAAGCATGTCCATGTTTCTCGCTTTAACGGAATCCACGCGAGAGAATGGCACAACTCGATTTTTCCCAGGA
AGTCATCTGTGGGACTACAGCCAAGATTTTCCAAGTGCTGATGACACTCGGATTCGTTACGCTGAACTGCATCCTGGC
GACTGTTACTTCATGCTCAGCAGCGTAACTCATAGCAGTACCGACAACCGGAGTACCAATCGACCACGAGTTCTCGCA
GCGACGATTGTCACTCGGAGCCACCTGCGGCAAGAGGAGAACCAATATCTCACATATGACCCGATAACAGTCGGAAG
GTTTCCCACGTGGCTCCAGAGGCTCGTGGGCTACGCGCCATCAGCACCATTCCTCGGTTGGGTGGACAAGCGGGAT
CCTCGATGTGTTATTGACCCCAAGGCGGCTGATGATCACTGTGGTGGAGAATATTATGAGACGAACGAAGAGACCTTG
AATTAA

## b

MGRDQVSHKRSQNSNVSEIPDLDSSLHRSEDTLVAEIIEAMTLAGVCVVRNLFTKSLVDQVLKDFEPHVSSTKLFDGYPGN
GCHLTGLLSKSEIYAHMVVGNSVFEKVRNHFLSTTFRSWIGGKMMTFTSPPQLDSTICSYINPQSPGEHLHRDDAIHYGWN
EAASEYTVGRDISMSMFLALTESTRENGTTRFFPGSHLWDYSQDFPSADDTRIRYAELHPGDCYFMLSSVTHSSTDNRSTN
RPRVLAATIVTRSHLRQEENQYLTYDPITVGRFPTWLQRLVGYAPSAPFLGWVDKRDPRCVIDPKAADDHCGGEYYETNEE
TLN*

Supplementary Figure 1. Sequence revision of *nvfE.* **a**, Revised DNA sequence of *nvfE.* The introns are highlighted in yellow. **b**, Revised protein sequence of NvfE.

**a**

ATGGTTGGATCACGGACCTGGTGTGAAAGTGAGATGCTCTTTGTACAGCCCGATGCAGGCACCAAAGAGGAGTTATAT

TATCGCGTTACCCCCAAGCCTGGCCAGACTCAGGCAAACTTCAACTGGACTCCTCACAAGGTCCGATTCCATGATGCC

CGTCCCCAACGTGATTCGTTCGATCTGAACACCCATGGTTTTACGTTCGTCGAAGATGCCATTTCTCCGCAGCTTATTG

AACGTATCAGGGCAGACGACACAGCTGCAGTTGAAGGGGATTACTTTGCTTCTGTGGCTGCGCTCGTGAAGCGAGTT

ACTGGGGCTGACCATGTCGTCTGTTTCAGCCCCTACACTCGCAAGGAGAACTCGGAAAAGGGAATATTCGGACAGCC

TGCCAGAACGGTAGGTATTATTCCCTTTCACAATTGAAACACAAACTTATCCGTTTTATCAGGTCCACTGCGATCAGTAC

GCCACTATCTACATGTTGTCCTTCCTATCCACTAACACTCTTATCCAGCACTCCAGCAGCAGCCATTGAACTGACTCATA

AGCTCTGTGGCGAGGATGCAGTTCGGCTATTGCAGTCTCGTTTCCGCGCATTCAGGTAATTGCCCAGGATTTTCATGAT

ACAACAGCTTACAGTTATTGCGGACGAACAGTGTATGGCGCCCTTTAGTCGAGCCGGTGCTCGACTGGCCTTTAGCCG

TTGTTGATGGACGAACCATAGCCCCCGACGACTTGCACCCGGTCCACTGGCTCAGGTACGAGAAGAAGGATACCGAA

CCACCCTTCCAACTCAGCTTCTCGGAGACGCAGAAATGGTATTATCTCAGTAGACAGCGCAGTGATGAGGTCAGCATC

GTTAAAAACTATGATTCGGAAGTTGTCCCATCTCCACGTACGTGACTGTCCCTTTTTTTCCGCATTTTCCAACGTACCCT

CAAGATCATCATGACCAAGATATGACTAACCGGGCACTAGGGTCGGCCCATTGCGCCTTTAAGCACCCGTTCGTACCTA

AAGATGCTCCTCCCCGCGAGAGCATTGATGTCAGATGTCTTGTTTTTGGAGGGCGTTAG

**b**

MVGSRTWCESEMLFVQPDAGTKEELYYRVTPKPGQTQANFNWTPHKVRFHDARPQRDSFDLNTHGFTFVEDAISPQLIE

RIRADDTAAVEGDYFASVAALVKRVTGADHVVCFSPYTRKENSEKGIFGQPARTVHCDHTPAAAIELTHKLCGEDAVRLLQS

RFRAFSVWRPLVEPVLDWPLAVVDGRTIAPDDLHPVHWLRYEKKDTEPPFQLSFSETQKWYYLSRQRSDEVSIVKNYDSE

VVPSPRSAHCAFKHPFVPKDAPPRESIDVRCLVFGGR*

Supplementary Figure 2. Sequence revision of *nvfI*. **a**, Revised DNA sequence of *nvfI*. The “C” base in red is missing in the database sequence. The introns are highlighted in yellow. **b**, Revised protein sequence of NvfI.

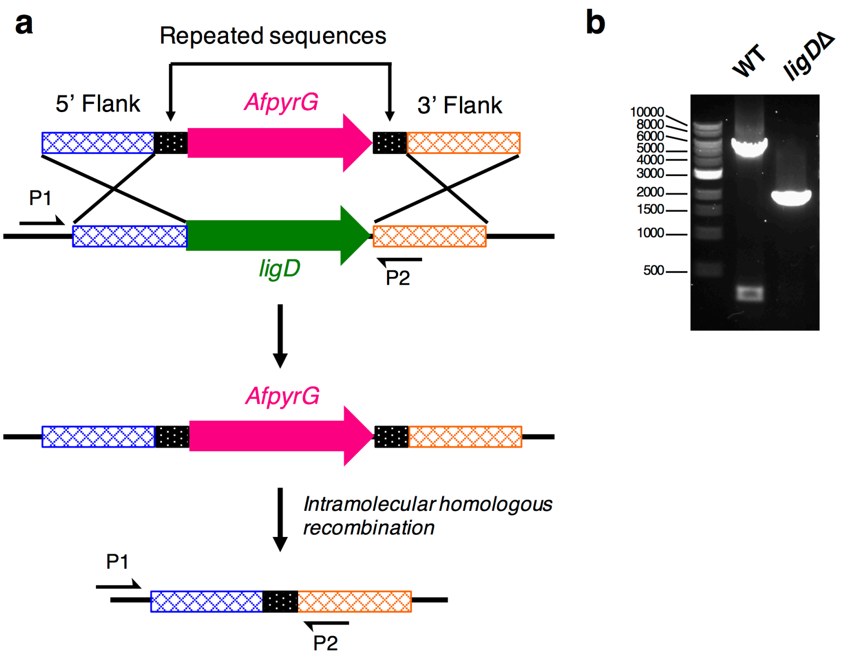


Supplementary Figure 3. Deletion of *ligD* in *A. novofumigatus*. **a**, Procedure for the deletion of *ligD* using *AfpyrG* as a selection marker and the elimination of *AfpyrG* by intramolecular homologous recombination to construct the *pyrG*$^{-}$, *ligD*Δ strain. **b**, Result of diagnostic PCR performed with the two primers P1, binding outside of the 5' flanking region of *ligD* used for the homologous recombination, and P2, binding to the 3' flanking region of *ligD* (see Supplementary Data 1 for the primer sequences).

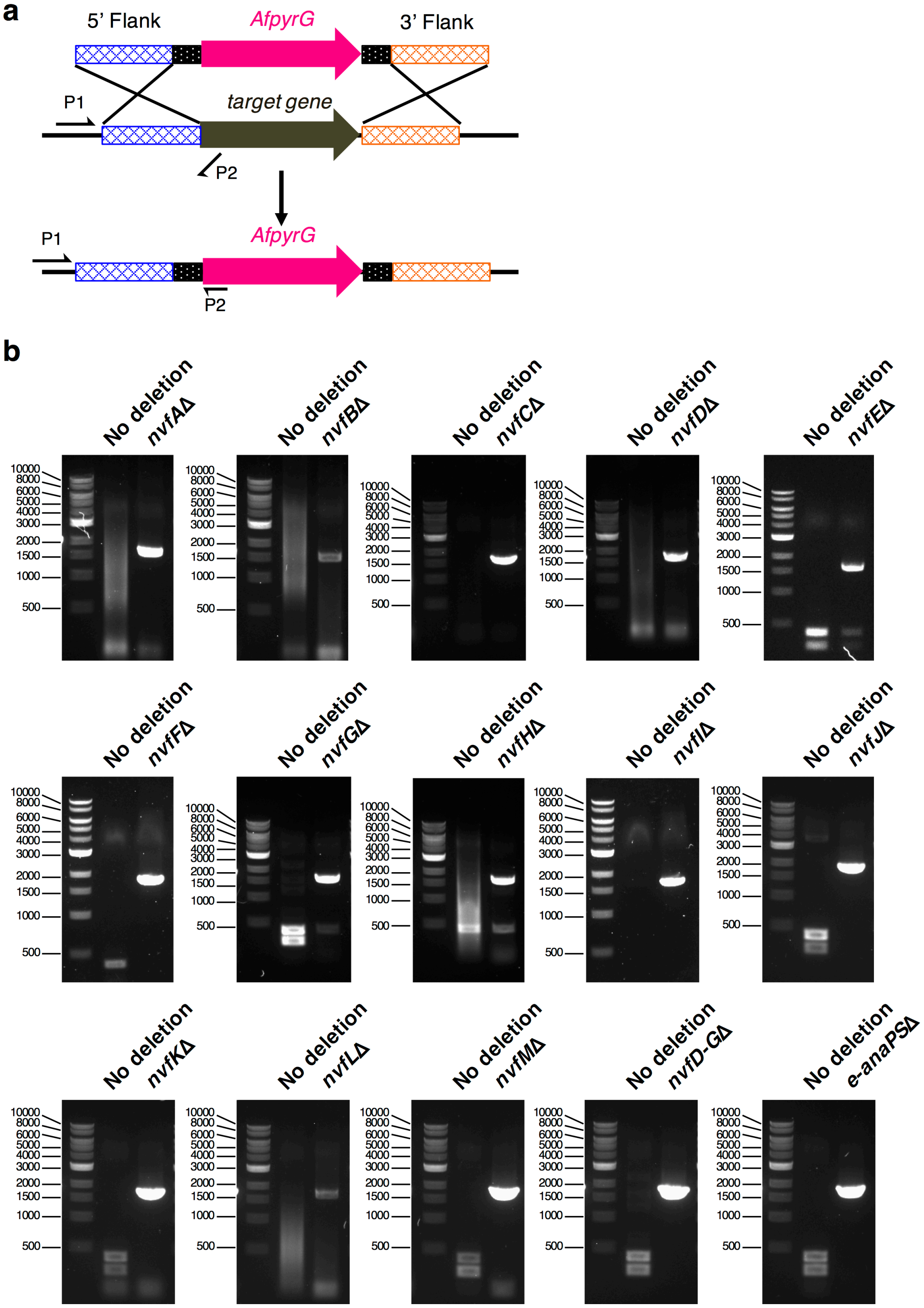


Supplementary Figure 4. Deletion of the genes in the *nvf* cluster and *e-anaPS*. **a**, Procedure for the deletion of each biosynthetic gene using *AfpyrG* as a selection marker. **b**, Result of diagnostic PCR performed with the two primers P1, binding outside of the 5' flanking region of each targeted gene used for the homologous recombination, and P2, binding to the promoter region of *AfpyrG* (see Supplementary Data 1 for the primer sequences). Since P2 does not bind to the wild type genome, a band (~1700 bp) is only amplified upon successful deletion of targeted gene.

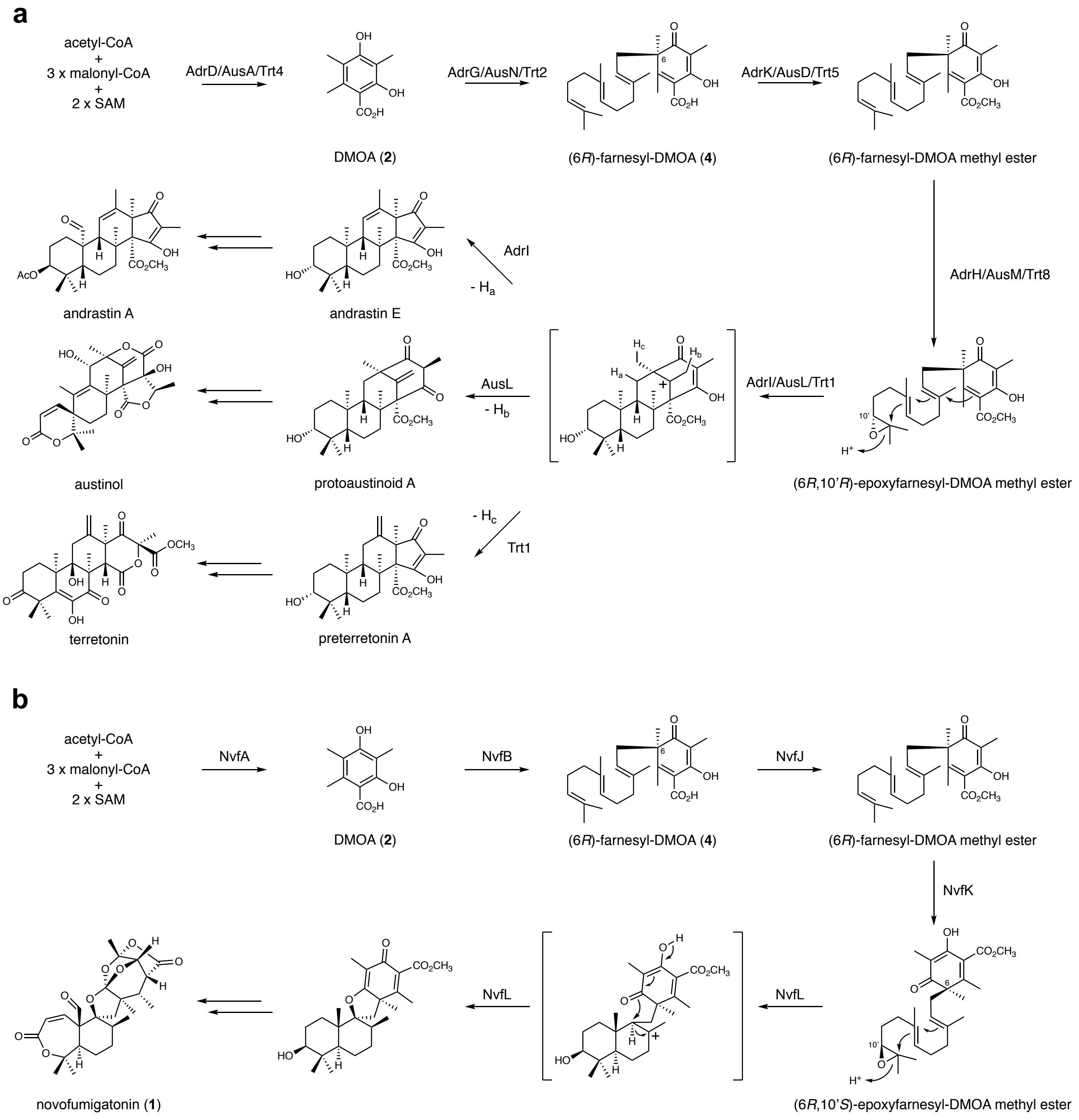


Supplementary Figure 5. Biosynthesis of DMOA-derived meroterpenoids. **a**, Early-stage biosynthesis of andrastin A, austinol, and terretonin. Methyl esterification by AdrK/AusD/Trt5 is essential before the cyclization of the farnesyl moiety. **b**, Predicted early-stage biosynthesis of novofumigatonin (**1**) based on those for the other DMOA-derived meroterpenoids.

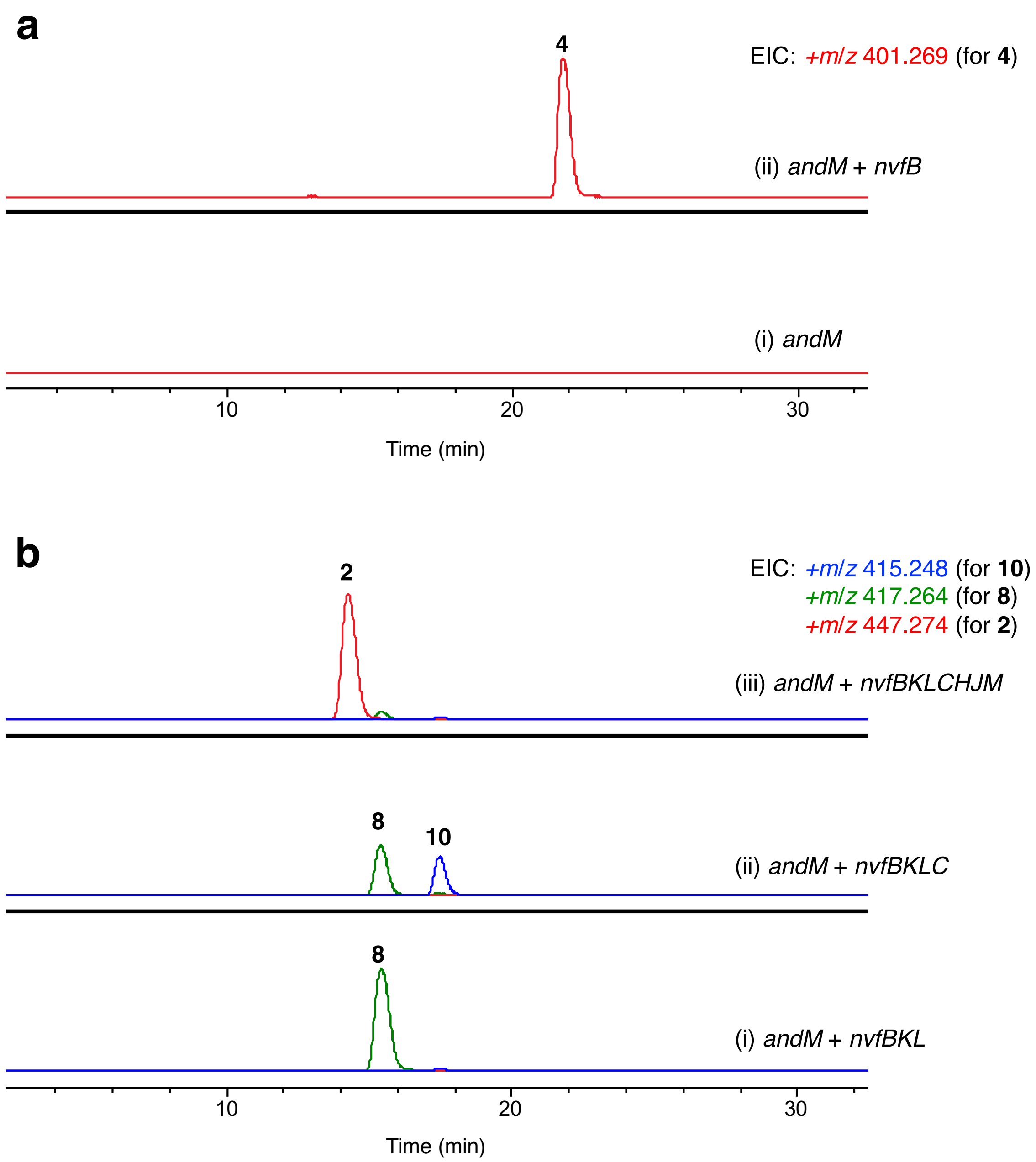


Supplementary Figure 6. Heterologous expression of the novofumigatonin biosynthetic genes in *A. oryzae.* **a**, LC-MS profiles of culture supernatant extracts from *A. oryzae* transformants harboring (i) *andM*; (ii) *andM*+*nvfB*. **b**, LC-MS profiles of culture supernatant extracts from *A. oryzae* transformants harboring (i) *andM*+*nvfBKL*; (ii) *andM*+*nvfBKLC*; (iii) *andM*+*nvfBKLCHJM*.

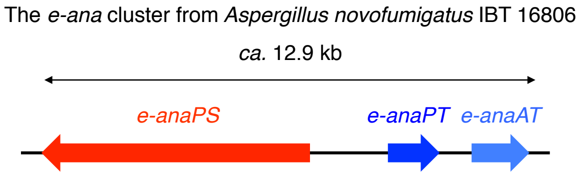


Supplementary Figure 7. Schematic representation of the *e-ana* cluster. The direction of the arrow indicates the direction from the start to the stop codon.

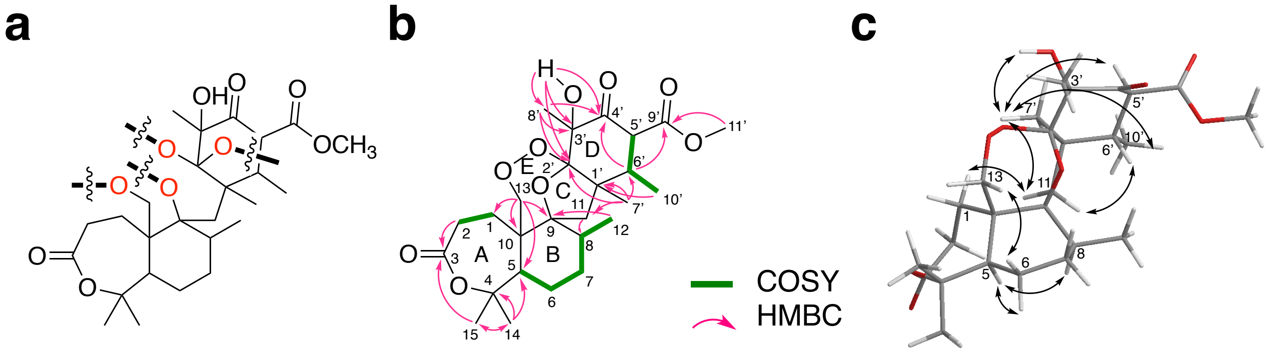


Supplementary Figure 8. Structural determination of **14**. **a**, Partial structure of **14** confirmed by NMR analysis. Four oxygen atoms in red color were not completely placed in the structure. Given the total number of oxygen atoms in **14**, two out of the four oxygen atoms must be identical. **b**, Key $^{1}$H-$^{1}$H DQF-COSY/HMBC correlation for **14**. **c**, Key NOESY correlations for **14**.

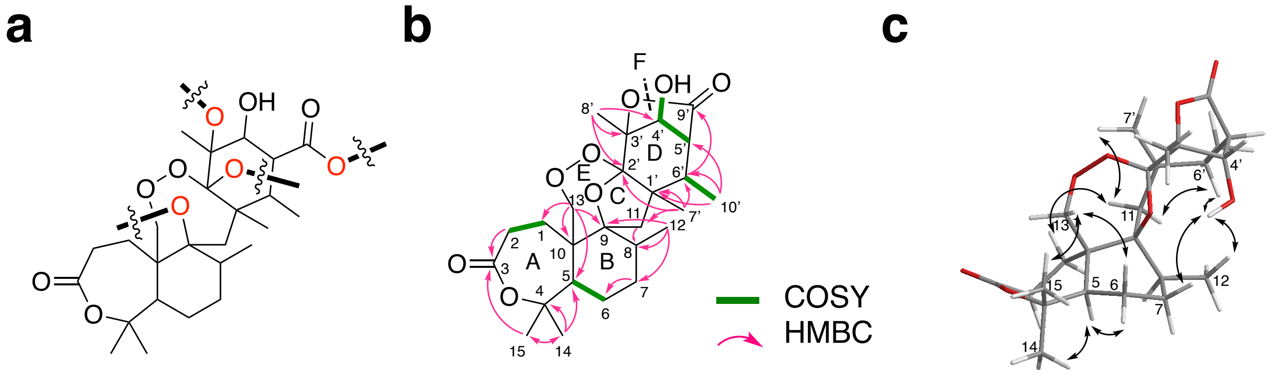


Supplementary Figure 9. Structural determination of **15**. **a**, Partial structure of **15** confirmed by NMR analysis and by the reduction of peroxide. Four oxygen atoms in red color were not completely placed in the structure. Given the total number of oxygen atoms in **15**, two out of the four oxygen atoms must be respectively identical with one of the remaining two oxygen atoms. **b**, Key $^{1}$H-$^{1}$H DQF-COSY/HMBC correlations for **15**. **c**, Key NOESY correlations for **15**.

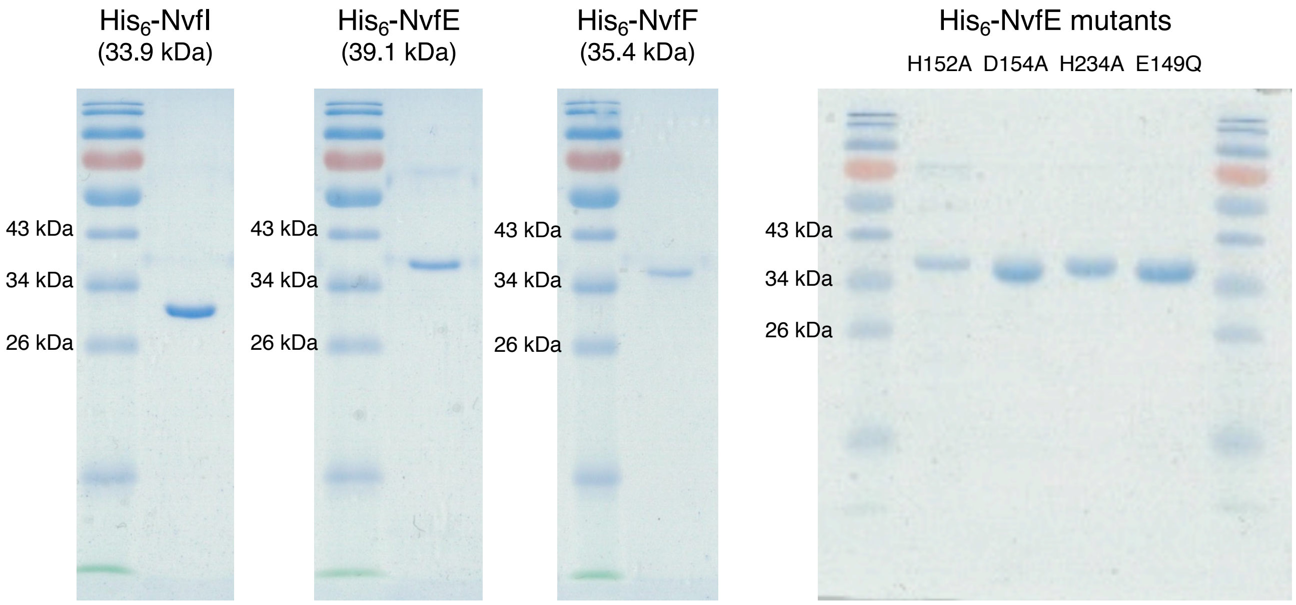


Supplementary Figure 10. SDS-PAGE analysis of the purified proteins. Selected enzymes that are important for the orthoester synthesis as well as NvfE mutants were purified from the *E. coli* expression system.

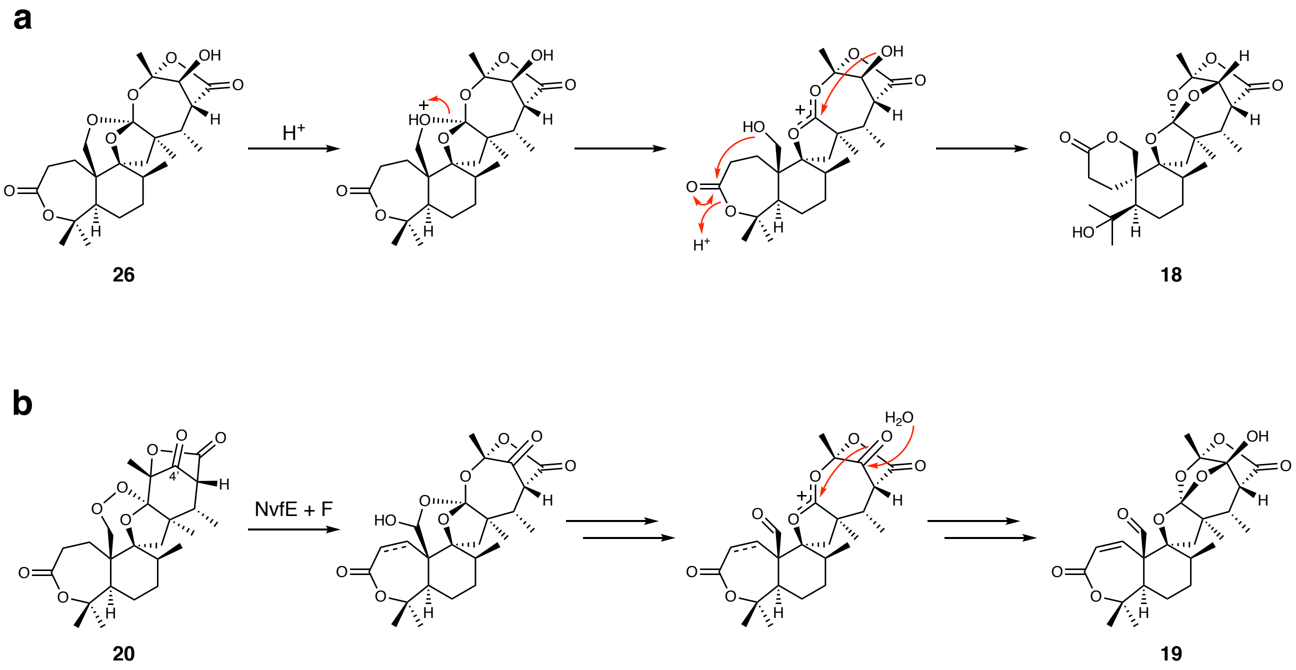


Supplementary Figure 11. Proposed mechanisms for the biogenesis of shunt products from the novofumigatonin pathway. **a**, Proposed mechanism to give asnovolin G (**18**) obtained from the *A. novofumigatus nvfF*Δ strain. **b**, Proposed mechanism to give novofumigatonol (**19**) obtained from the *A. novofumigatus nvfG*Δ strain.

```
NvfE (Aspergillus novofumigatus)     1 MGRDQVSHKRSQNSNVSEIPDLDSSLHRSEDTLVAEIIEAMTLAGVCVVRNLFTKSLVDQ
AusE (Aspergillus nidulans)          1 MG--SATPSRLQK--F------------PATAPADEIYAAFKEDGCVIIEGFVPPDQMAR
PrhA (Penicillium brasilianum)       1 MA--PMIPPRLQR--F------------PATASADEIFAAFQEDGCVVIEGFISPEQVAR
FtmF (Aspergillus fumigatus)         1 ---MTVDSKQLQR--L------------AADADVDRMCRLLEEDGAFILKGLLPFDVVES
AsqJ (Aspergillus nidulans)          1 MTSKDHVKSQIPR--L------------SAINDLHKIWPTVEEHGAAIIESFLSLDIVRR
EasH (Claviceps purpurea)            1 MTSQHQEHTGTKR--F------------SIQSDPVEIHRAIVEDGVAIIEGFLTPEQVQK

NvfE (Aspergillus novofumigatus)    61 VLKDFEPHVSSTKLFDG----------------YPGNGCHLTGLLSKSEIYAHMVVGNSV
AusE (Aspergillus nidulans)         45 FSQEIQPAMEKIQVQVTNDGN------------SNDRVKRFSKLVTTSPTFRHEILENDL
PrhA (Penicillium brasilianum)      45 FSQEVDPAMEKIPVEVTNNGN------------SNDRTKRFSKCVIASPTFRNEIIESDL
FtmF (Aspergillus fumigatus)        44 FNRELDVQMAIPPPKGE-------RLL---ADKYPPHFKYVPNVATTCPTFRNTVLINPV
AsqJ (Aspergillus nidulans)         47 LNEEVDPFVKIEPIPAA-------KTKDHPNHVLSTTTRLVNVLAPISKAYREDVLNSKV
EasH (Claviceps purpurea)           47 LNKDVDAPLKADREQLKFKADKKDDPHFWLADFIPDHVARVHNLVDFSHCFRHEILNHEL

                                                                                      *  * *
NvfE (Aspergillus novofumigatus)   105 FEKVRNHFLSTTFR---SWIGGKMMTFTSPPQLDSTICSYINPQSPGEHLHRDDAIHYGW
AusE (Aspergillus nidulans)         93 MHELLQRVFSKPGEGMGYHFNDT-------------MVIEVQPGAPAQRLHRDQE-LYPW
PrhA (Penicillium brasilianum)      93 MHELCDRVFSKPGEGMGYHFNDN-------------MVIEVQPGAPAQRLHRDQE-LYPW
FtmF (Aspergillus fumigatus)        94 IHAICEAYFQRTGD---YWLSAA-------------FLREIESGMPAQPFHRDD-ATHPL
AsqJ (Aspergillus nidulans)        100 LHRICSDAFHVYGD---YWVLMG-------------AVMELAPSNPAQPLHRDMRFSHPI
EasH (Claviceps purpurea)          107 LHKICRLTFEESGD---YWLGYG-------------AVIENGPGTTEQKWHRDQP-RYPL

NvfE (Aspergillus novofumigatus)   162 NEAASEYTVGRDISMSMFLALTESTRENGTTRFFPGSHLWDYSQDFPSAD-----DTRIR
AusE (Aspergillus nidulans)        139 WNSMG--PDAPECLVNFFCAVTPFTVENGATRLVPGSNRWPELTLINATDCPQYGKIDSV
PrhA (Penicillium brasilianum)     139 WNSMG--PAGPECVINFFCAVTPFTEENGATRLVPGSHLWPEFTQINERDCPQFGKIETV
FtmF (Aspergillus fumigatus)       137 MHYQP--LEAPPVSLSVIFPLTEFTEENGATEVILGSHRWTEVGTPERDQAVL-------
AsqJ (Aspergillus nidulans)        144 VEYLK--PDAPATSINFLVALSPFTAENGATHVILGSHKWQNLSNVSMDATVR-------
EasH (Claviceps purpurea)          150 V-KEG--PDAPEGMLNFFTALTDFDAETGKTQYILGSNKRVELGEPDADHPIE-------

                                                        *
NvfE (Aspergillus novofumigatus)   217 YAELHPGDCYFMLSSVTHSSTDNRST--NRPRVLAATIVTRSHLRQEENQYLTYDPITVG
AusE (Aspergillus nidulans)        197 PAIMQPGDCYMMSGKVIHGAGHNATL-SDQRRALAFS-TIRRELRPVQAFPLWIPMQIAT
PrhA (Penicillium brasilianum)     197 PAIMQPGDCYLMSGKVIHGAGHNATT-TDRRRALALA-IIRRELRPMQAFSLSVPMKLAR
FtmF (Aspergillus fumigatus)       188 -ATMDPGDVLIVRQRVVHAGGGNRTTAGKPRRVVLAY-FNSVQLTPFETY-RTMPREMVE
AsqJ (Aspergillus nidulans)        195 -ALMNPGDALLITDSTIHCGGAETT-GTETRRLLTIT-MGISQLTPLESN-LAVPRPVIE
EasH (Claviceps purpurea)          200 YVGLKPGDTTIVSGKITHRGSDNRS--DKMRRAMPIM-IIPSILTPFDAT-CHLSRELVE

NvfE (Aspergillus novofumigatus)   275 RFPTWLQRLVGYAPSAPFLG---------W-VDKRDPRCVIDPKAADDHCGGEYYETNEE
AusE (Aspergillus nidulans)        255 ELSPRTQAMFGFRSSTQHCDVD---TVHFWGNDGKDIGEHLGLISSA*------------
PrhA (Penicillium brasilianum)     255 EMSERSQTMFGFRSSVQHCDVD---MVHFWGNDGKDIAHHLGLEAPSVHV*---------
FtmF (Aspergillus fumigatus)       245 SMTVLGQRMLGWRTMKPSDPNIVGIN----LIDDKRLENVLQLKAADSPA*---------
AsqJ (Aspergillus nidulans)        251 SLTPLAQRLLGWASQRSAAPRDIGLL----TIRGNSIEKTMNLKAEQPLHDDEAEPLCRE
EasH (Claviceps purpurea)          256 TMTPLAQKMICRRSVMIPAPGTVEVKTGIWCVNMREAGEQIGLKSNQRAKEDAEATDA--

NvfE (Aspergillus novofumigatus)   325 TLN*
AusE (Aspergillus nidulans)            ----
PrhA (Penicillium brasilianum)         ----
FtmF (Aspergillus fumigatus)           ----
AsqJ (Aspergillus nidulans)        307 TI*-
EasH (Claviceps purpurea)          314 -V*-
```

Supplementary Figure 12. Sequence alignment between NvfE and selected αKG-dependent enzymes involved in fungal natural products biosynthesis. Blue asterisks indicate the conserved 2-His-1-carboxylate iron-binding triad, and red asterisk indicates the highly conserved glutamine residue important for the αKG binding.

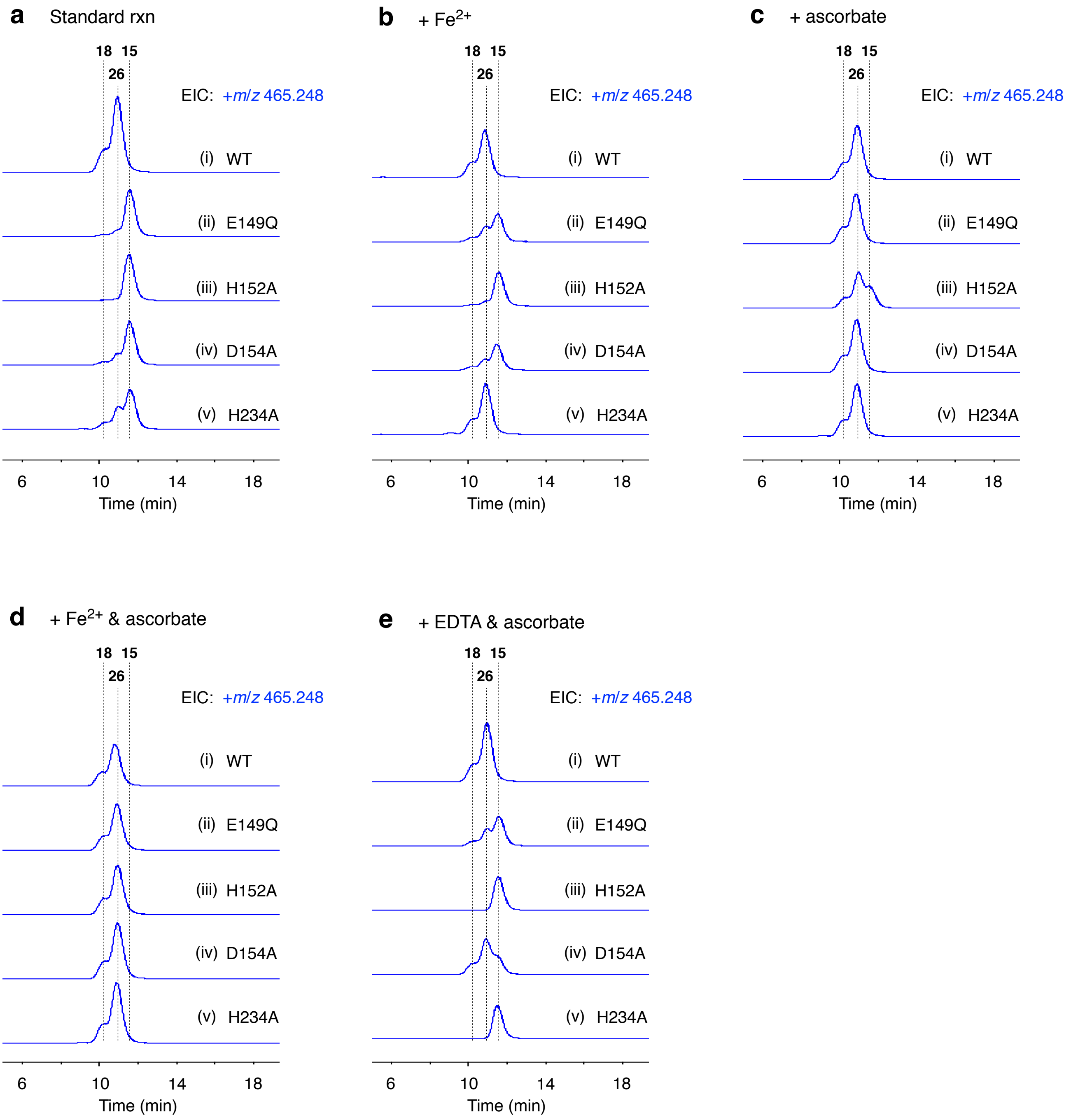


Supplementary Figure 13. LC-MS profiles of the products from *in vitro* enzymatic reactions of NvfE and its mutants with **15**. **a**, Standard reaction; **b**, with $FeSO_4$; **c**, with ascorbate; **d**, with both $FeSO_4$ and ascorbate; and **e**, with both EDTA and ascorbate.

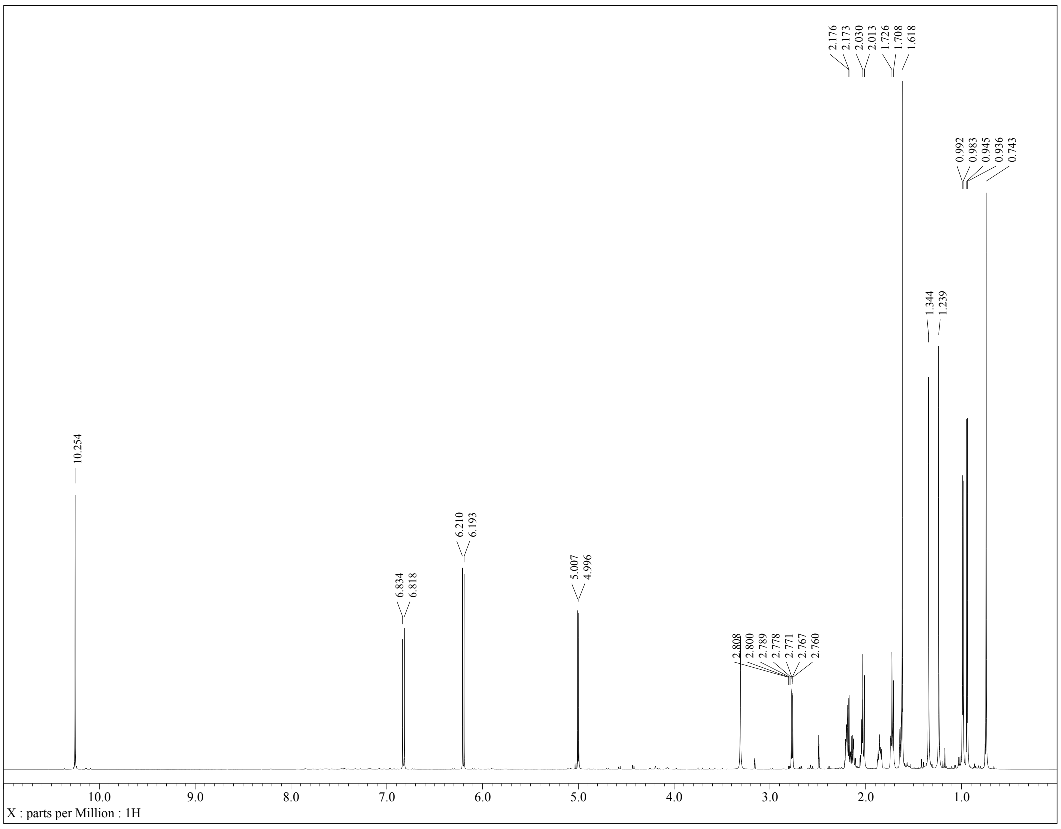


Supplementary Figure 14. $^{1}$H NMR spectrum of **1**. See Supplementary Table 5 for the NMR data.

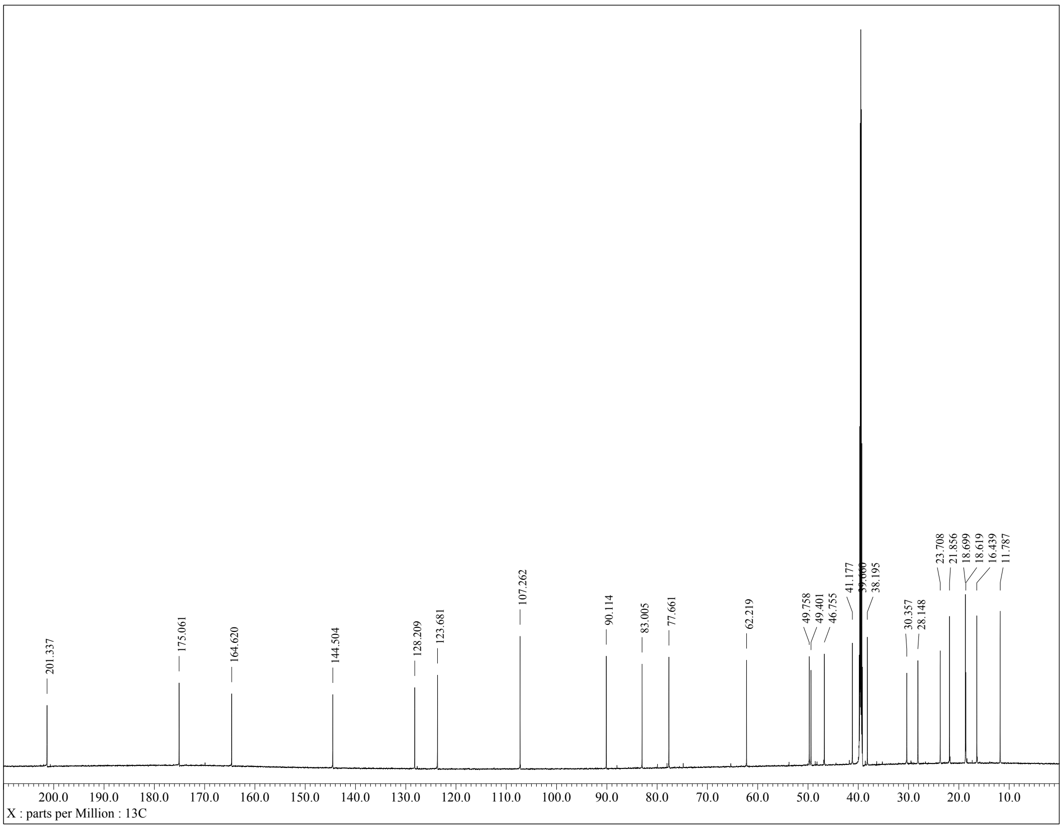


Supplementary Figure 15. $^{13}$C NMR spectrum of **1**. See Supplementary Table 5 for the NMR data.

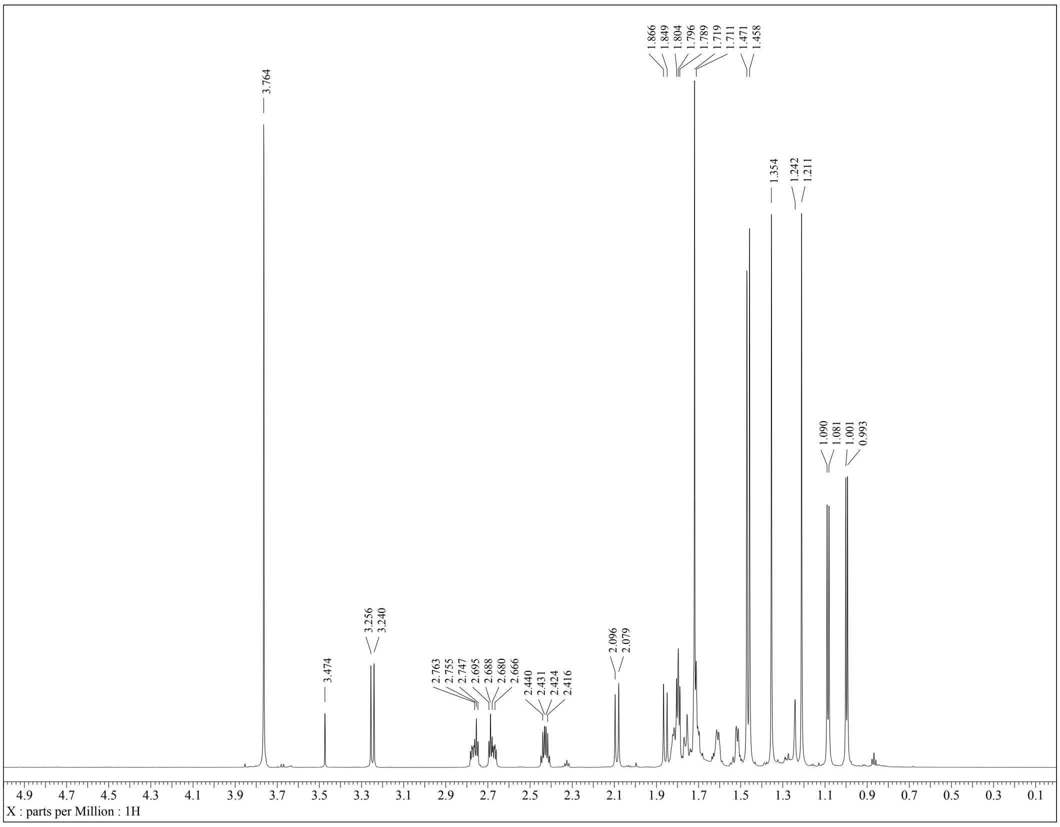


Supplementary Figure 16. $^{1}$H NMR spectrum of **2**. See Supplementary Table 6 for the NMR data.

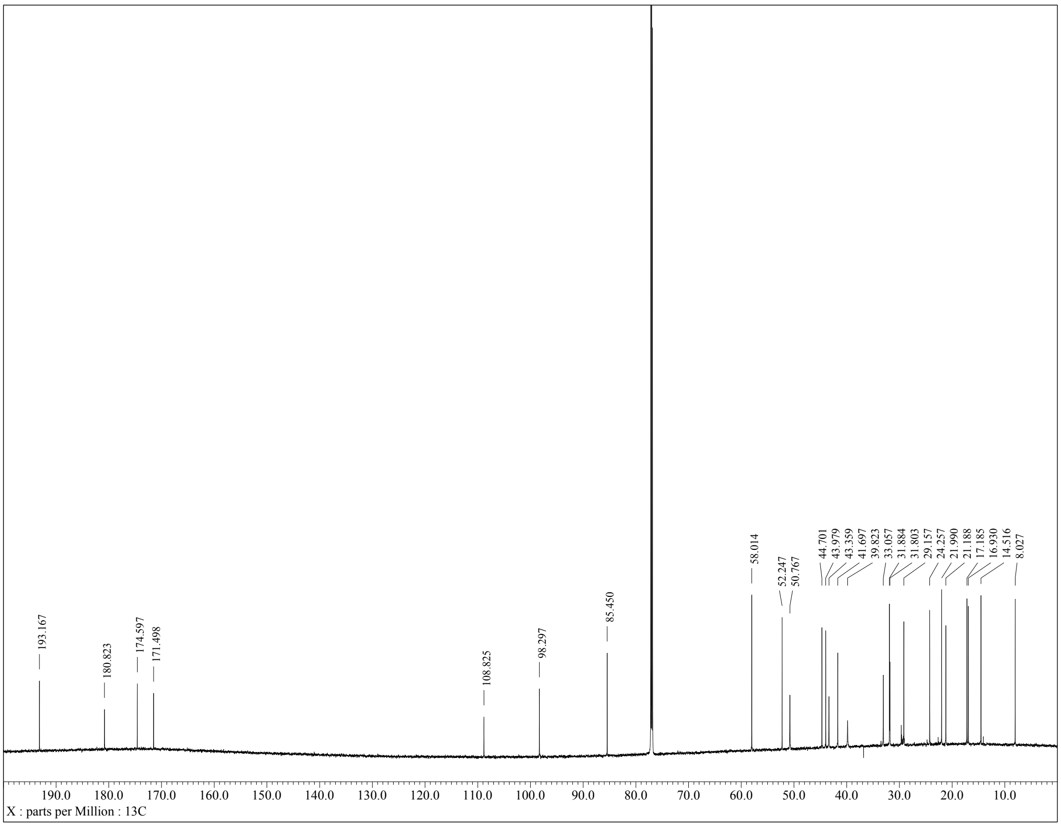


Supplementary Figure 17. $^{13}$C NMR spectrum of **2**. See Supplementary Table 6 for the NMR data.

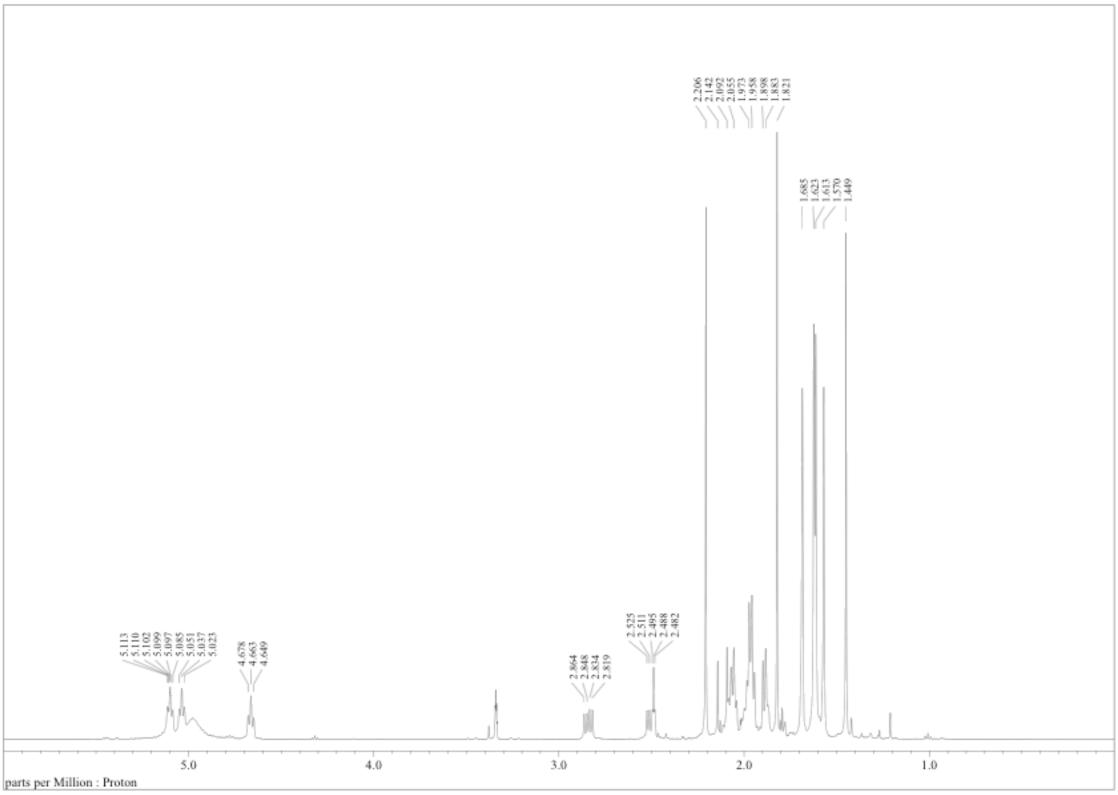


Supplementary Figure 18.$^{1}$H NMR spectrum of. See Supplementary Table 7 for the NMR data.

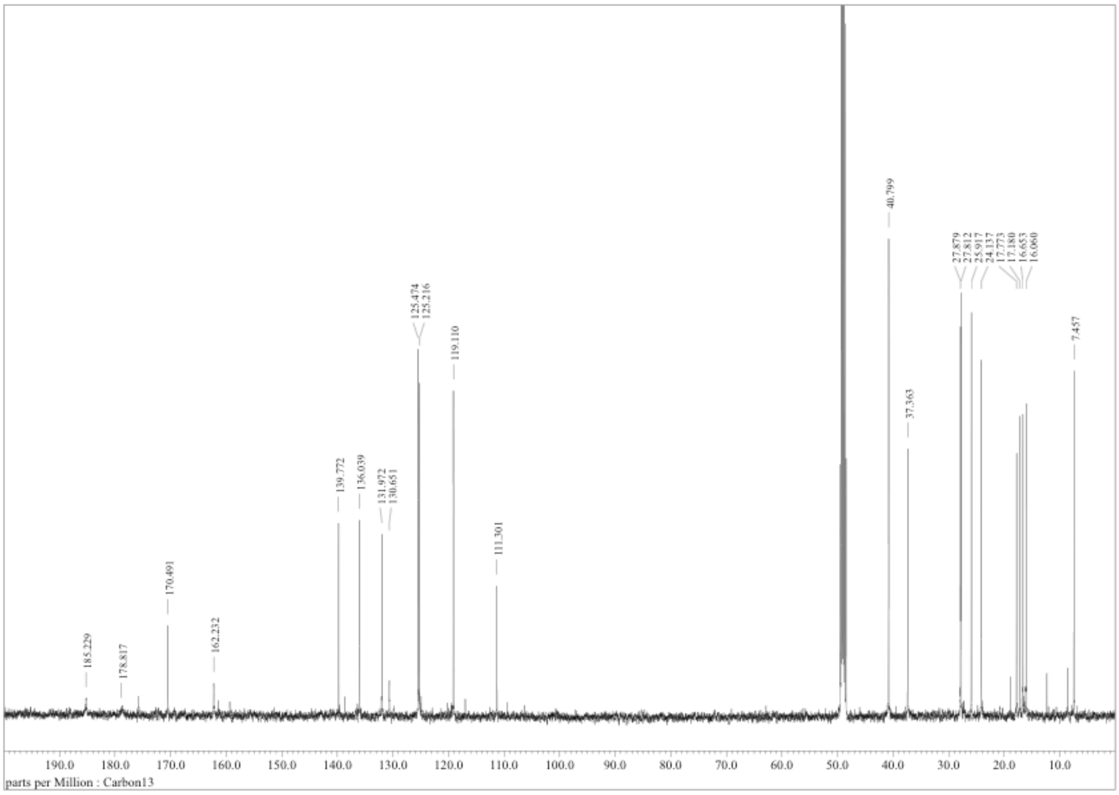


Supplementary Figure 19.$^{13}$C NMR spectrum of **4**. See Supplementary Table 7 for the NMR data.

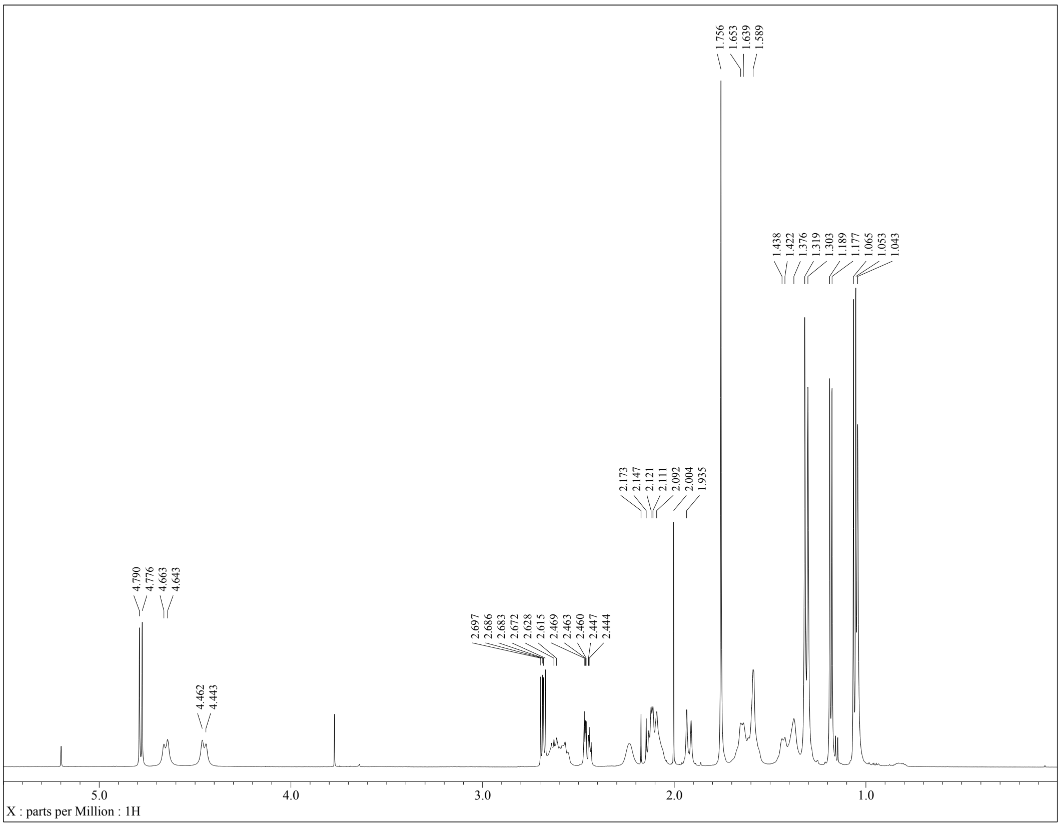


Supplementary Figure 20. $^{1}$H NMR spectrum of **18**. See Supplementary Table 8 for the NMR data.

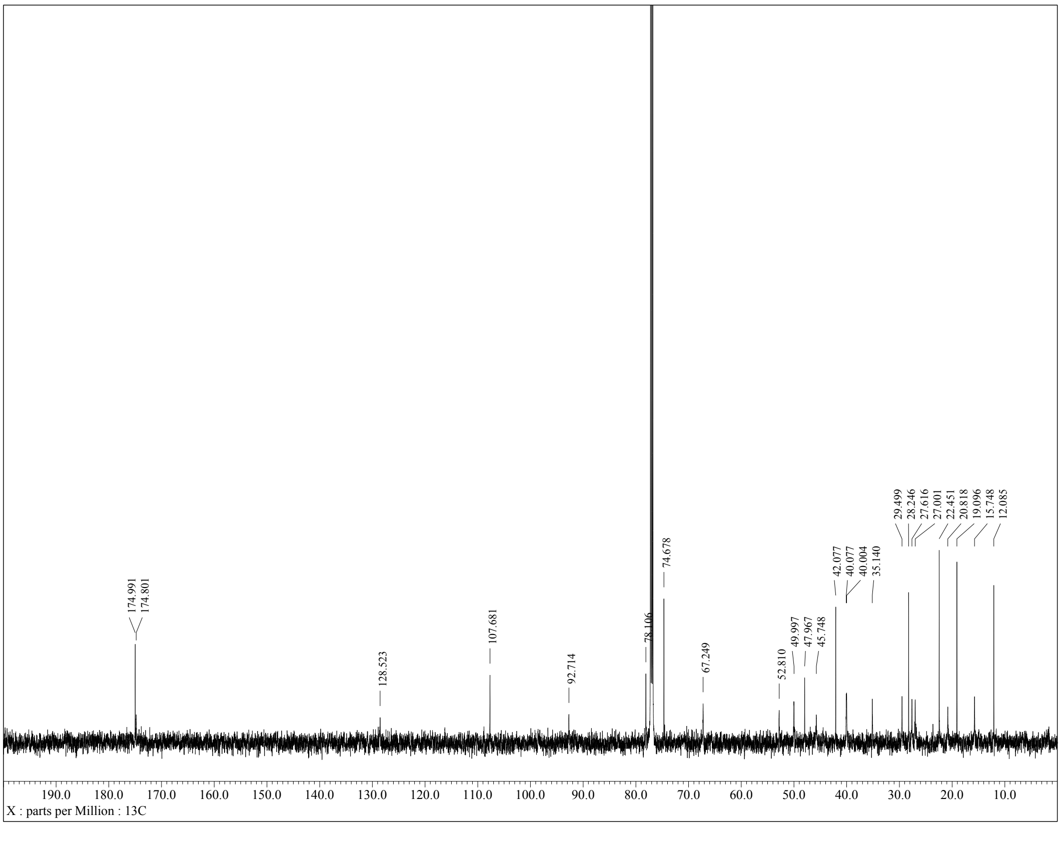


Supplementary Figure 21. $^{13}$C NMR spectrum of **18**. See Supplementary Table 8 for the NMR data.

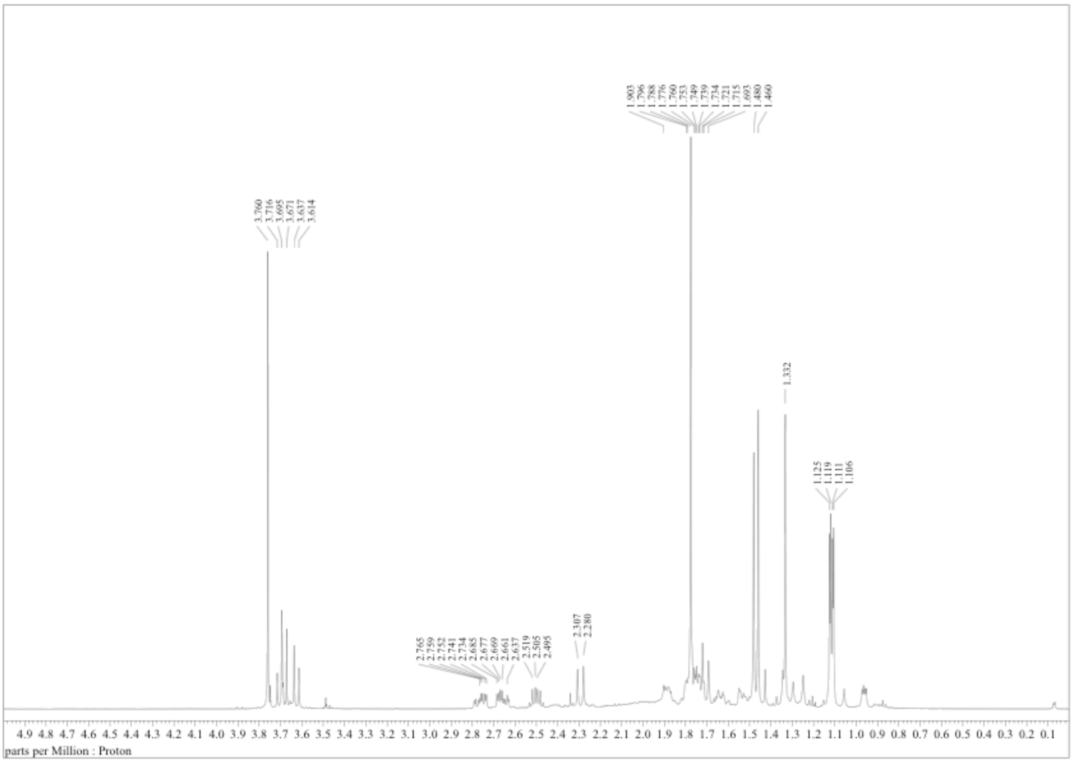


Supplementary Figure 22. $^{1}$H NMR spectrum of **3**. See Supplementary Table 9 for the NMR data.

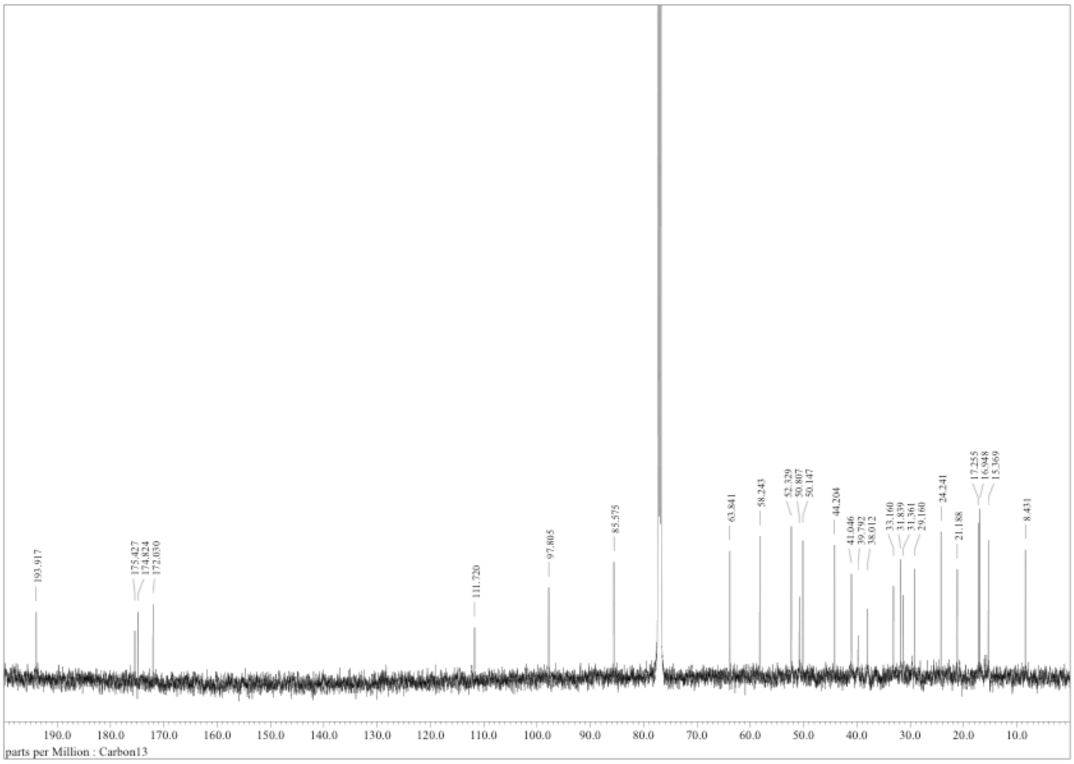


Supplementary Figure 23. $^{13}$C NMR spectrum of **3**. See Supplementary Table 9 for the NMR data.

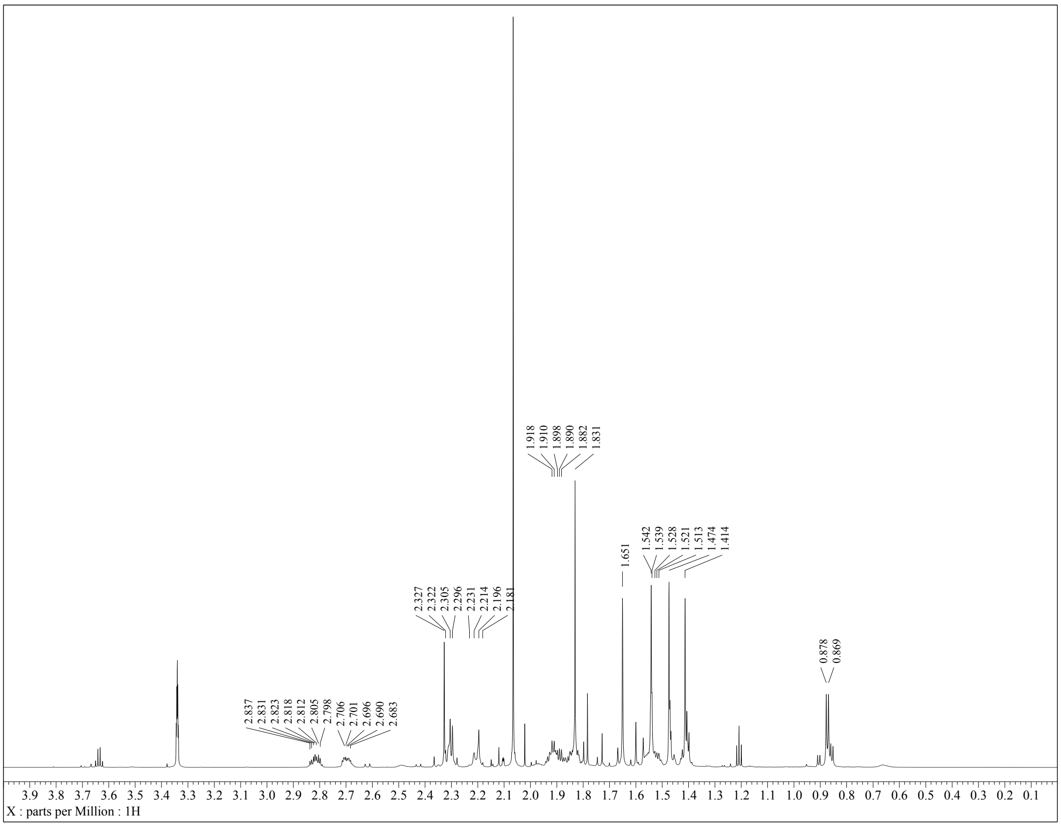


Supplementary Figure 24. $^{1}H$ NMR spectrum of **5**. See Supplementary Table 10 for the NMR data.

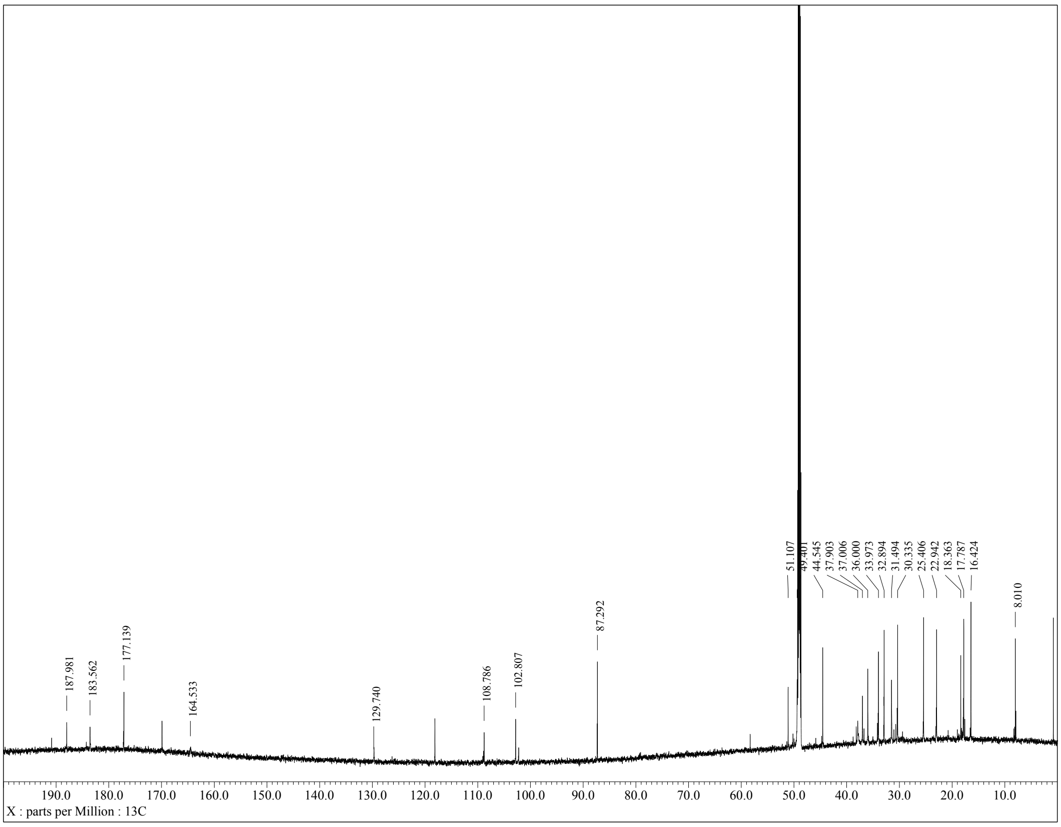


Supplementary Figure 25. $^{13}C$ NMR spectrum of **5**. See Supplementary Table 10 for the NMR data.

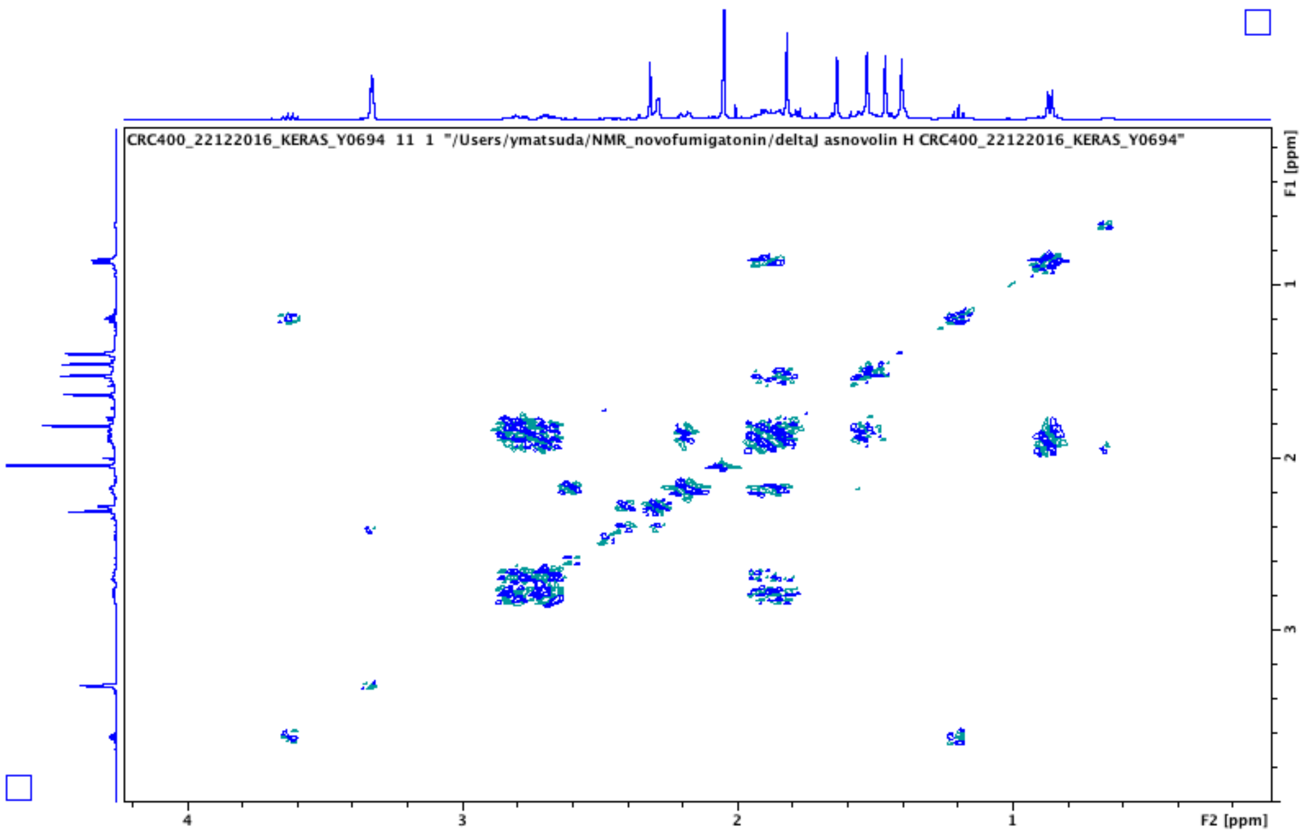


Supplementary Figure 26. $^{1}H$-$^{1}H$ DQF-COSY spectrum of **5**. See Supplementary Table 10 for the NMR data.

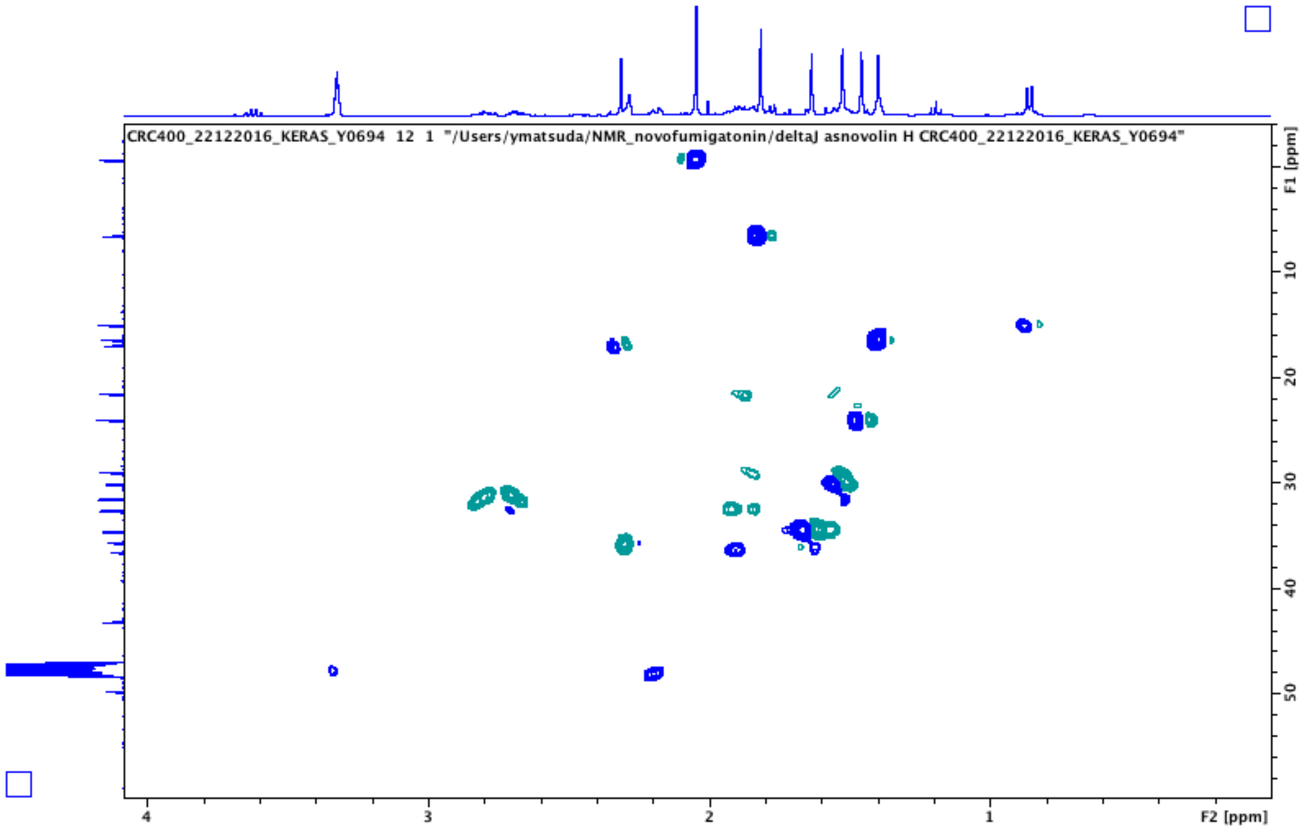


Supplementary Figure 27. HSQC spectrum of **5**. See Supplementary Table 10 for the NMR data.

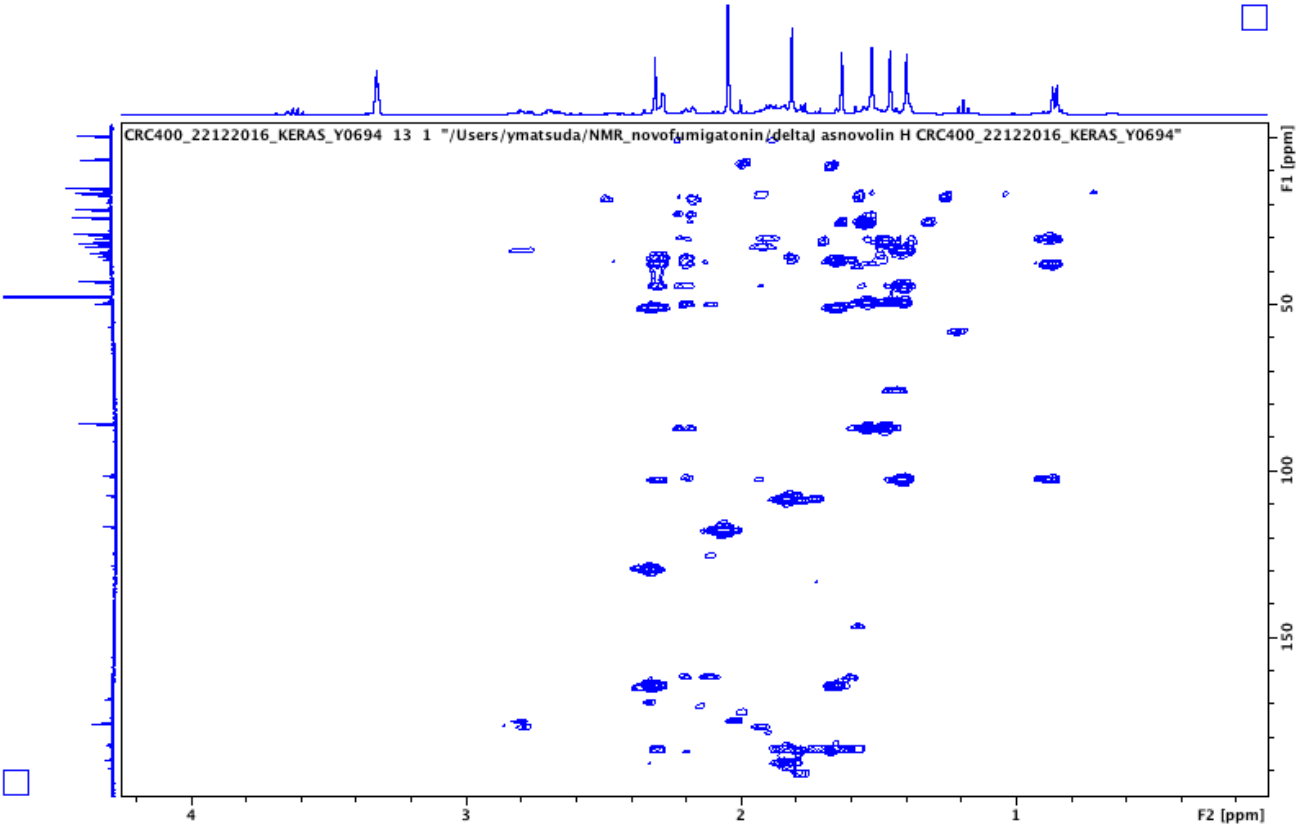


Supplementary Figure 28. HMBC spectrum of **5**. See Supplementary Table 10 for the NMR data.

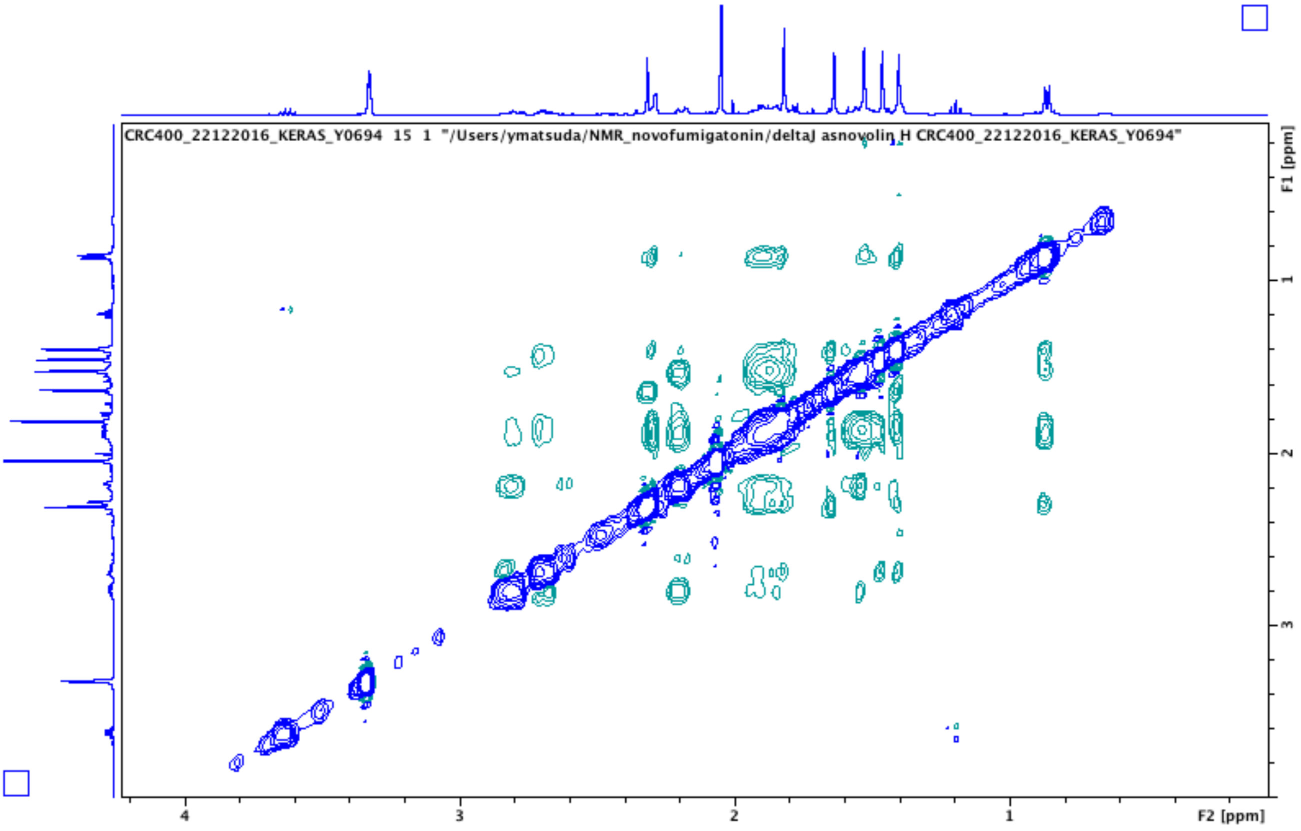


Supplementary Figure 29. NOESY spectrum of **5**. See Supplementary Table 10 for the NMR data.

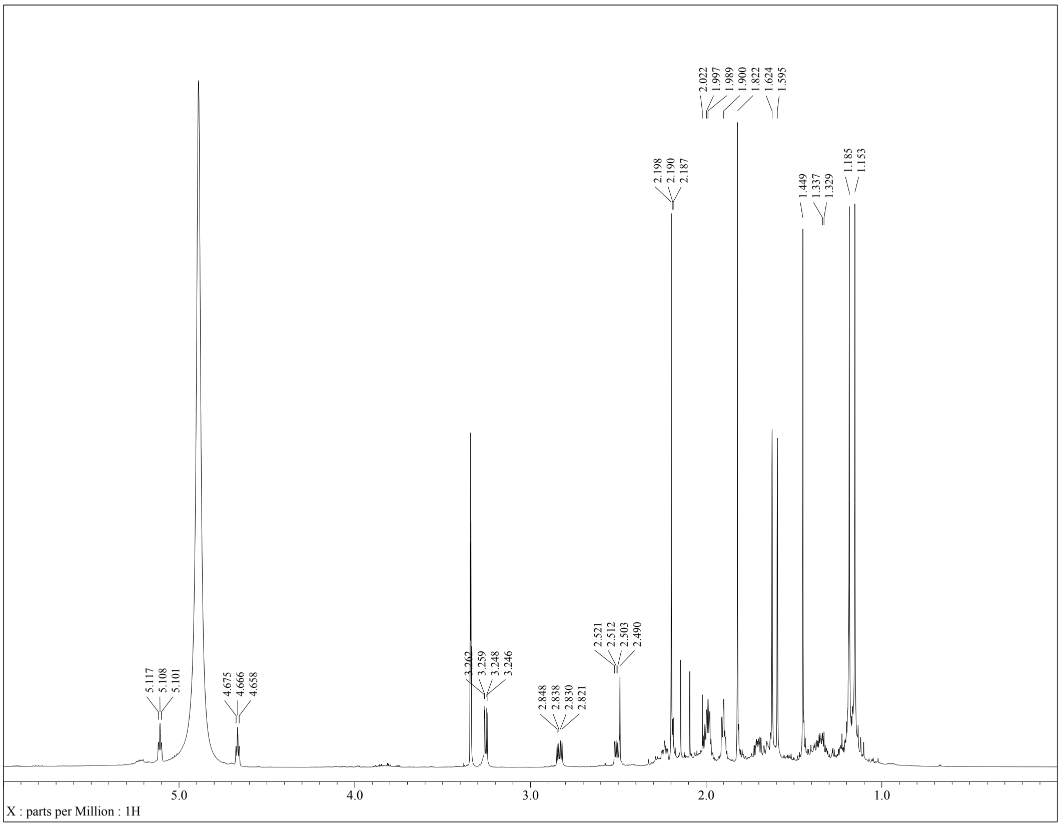

Supplementary Figure 30. $^{1}$H NMR spectrum of **6**. See Supplementary Table 11 for the NMR data.

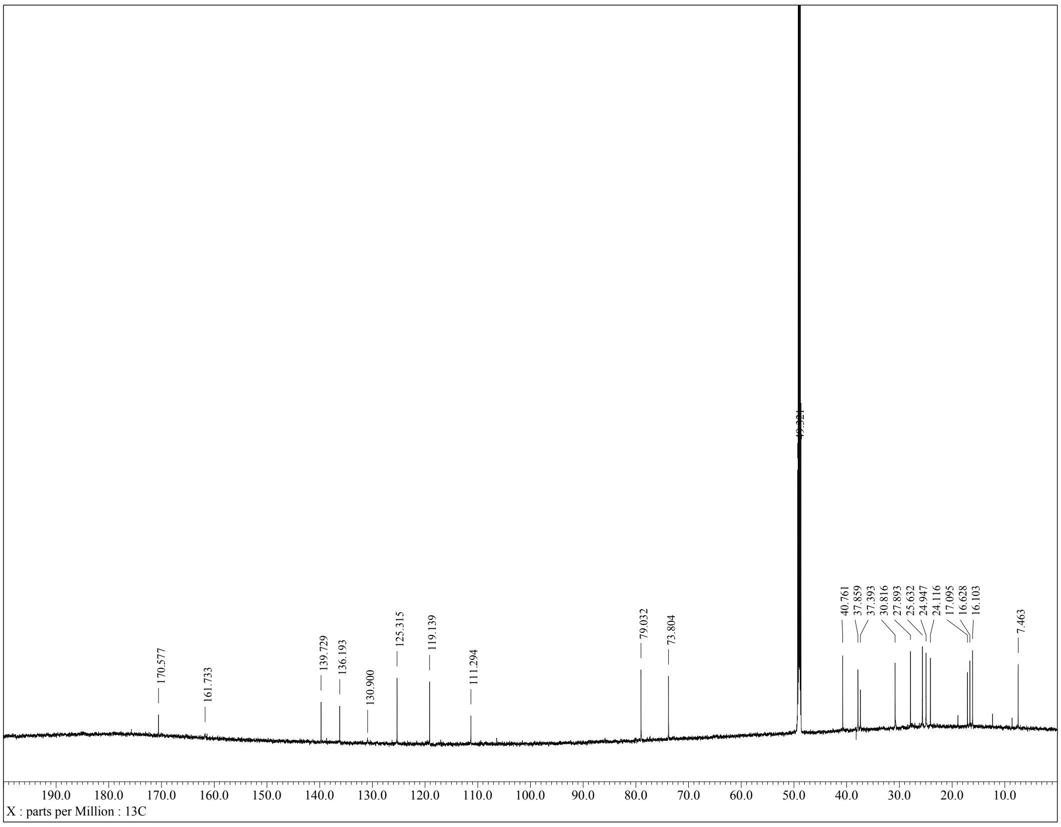

Supplementary Figure 31. $^{13}$C NMR spectrum of **6**. See Supplementary Table 11 for the NMR data.

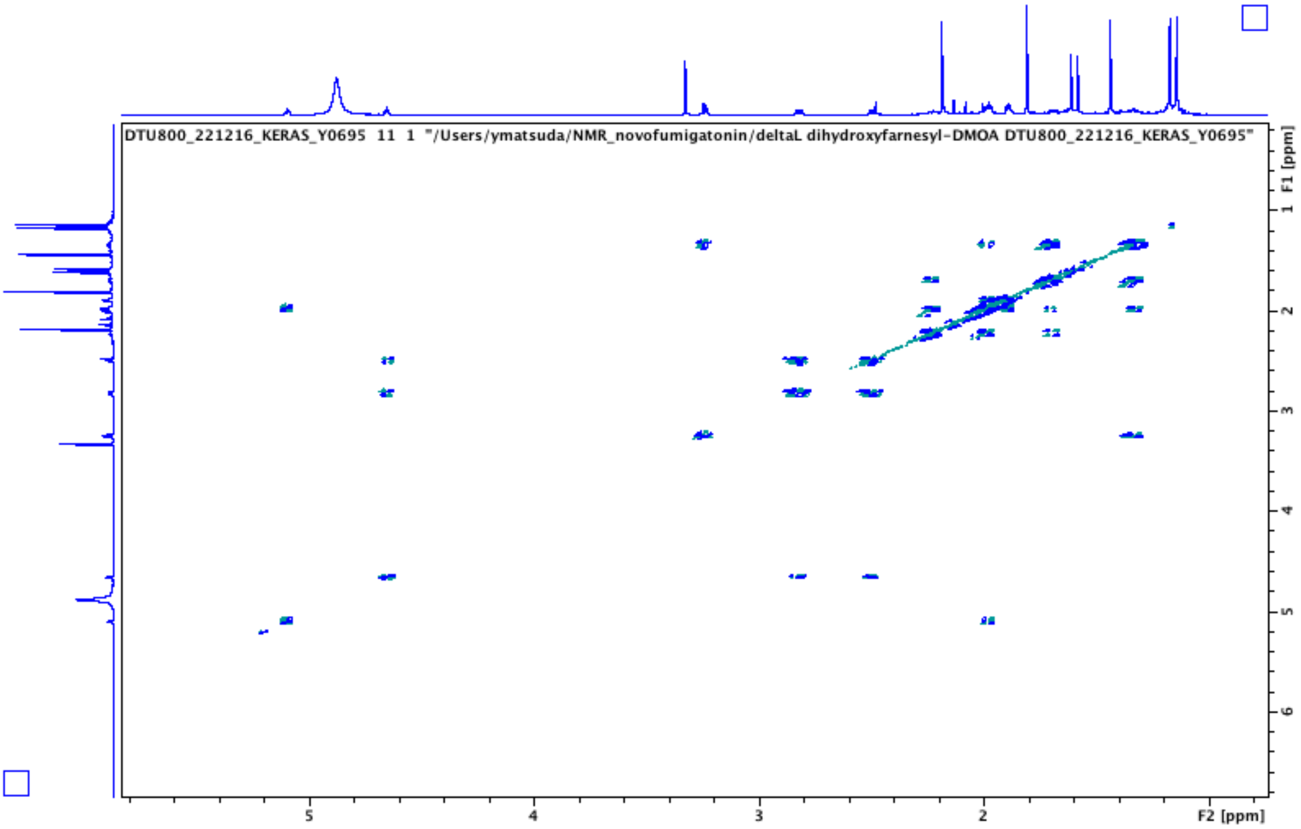


Supplementary Figure 32. $^{1}H$-$^{1}H$ DQF-COSY spectrum of **6**. See Supplementary Table 11 for the NMR data.

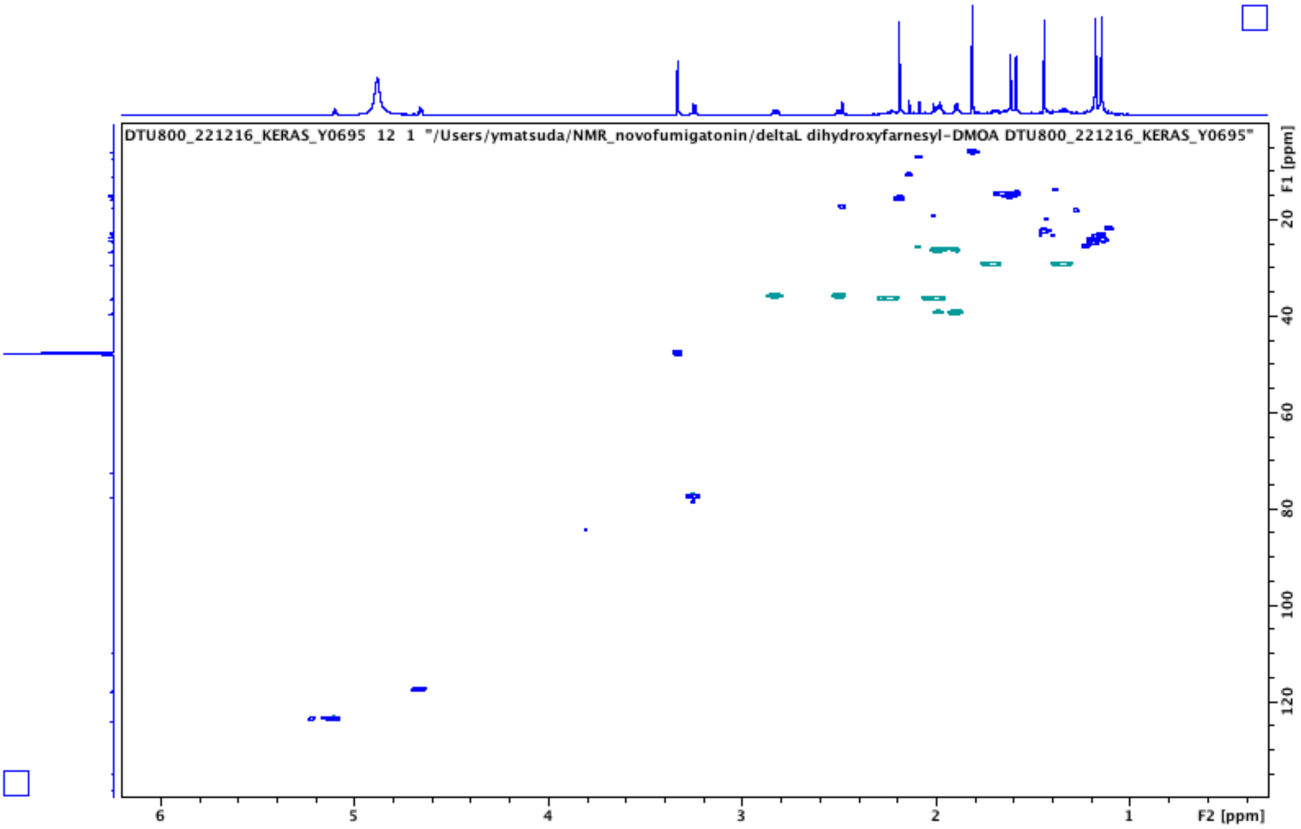


Supplementary Figure 33. HSQC spectrum of **6**. See Supplementary Table 11 for the NMR data.

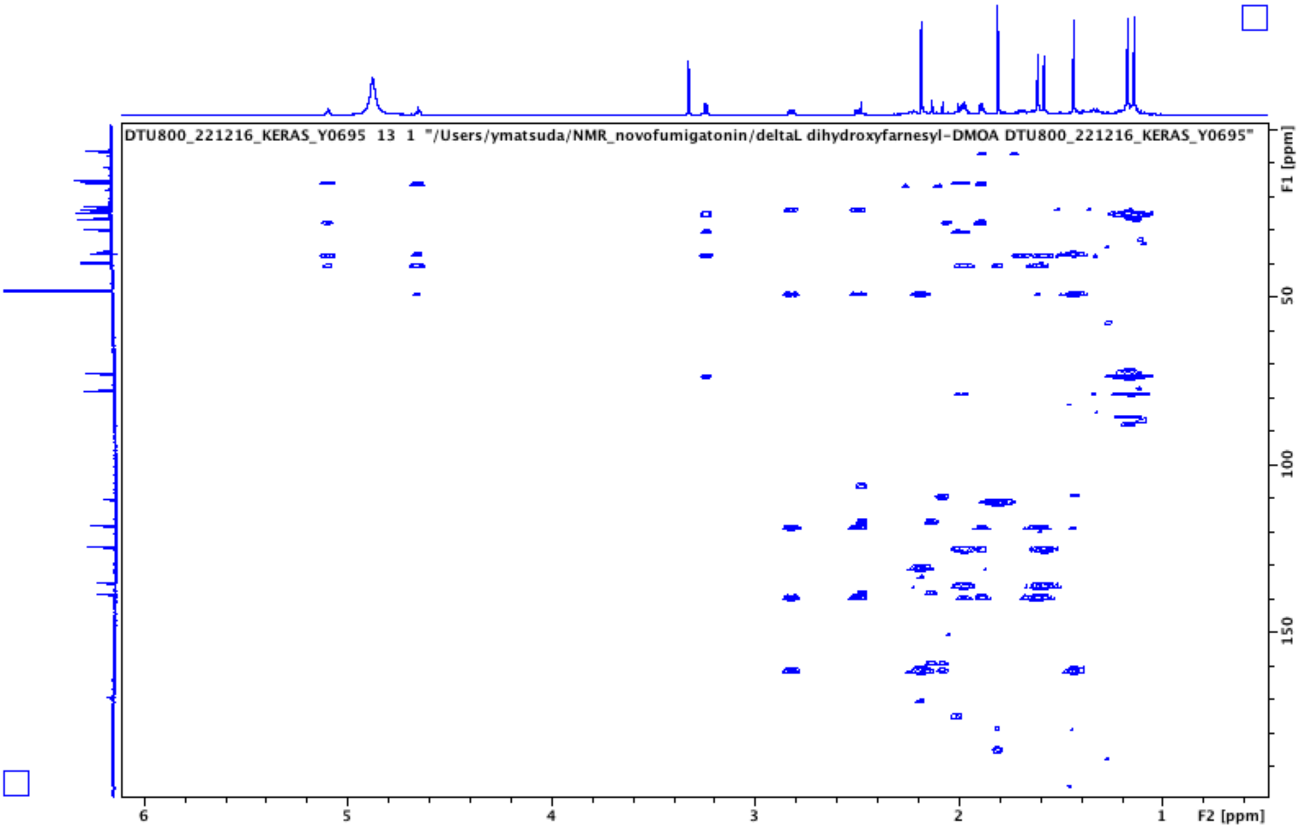


Supplementary Figure 34. HMBC spectrum of **6**. See Supplementary Table 11 for the NMR data.

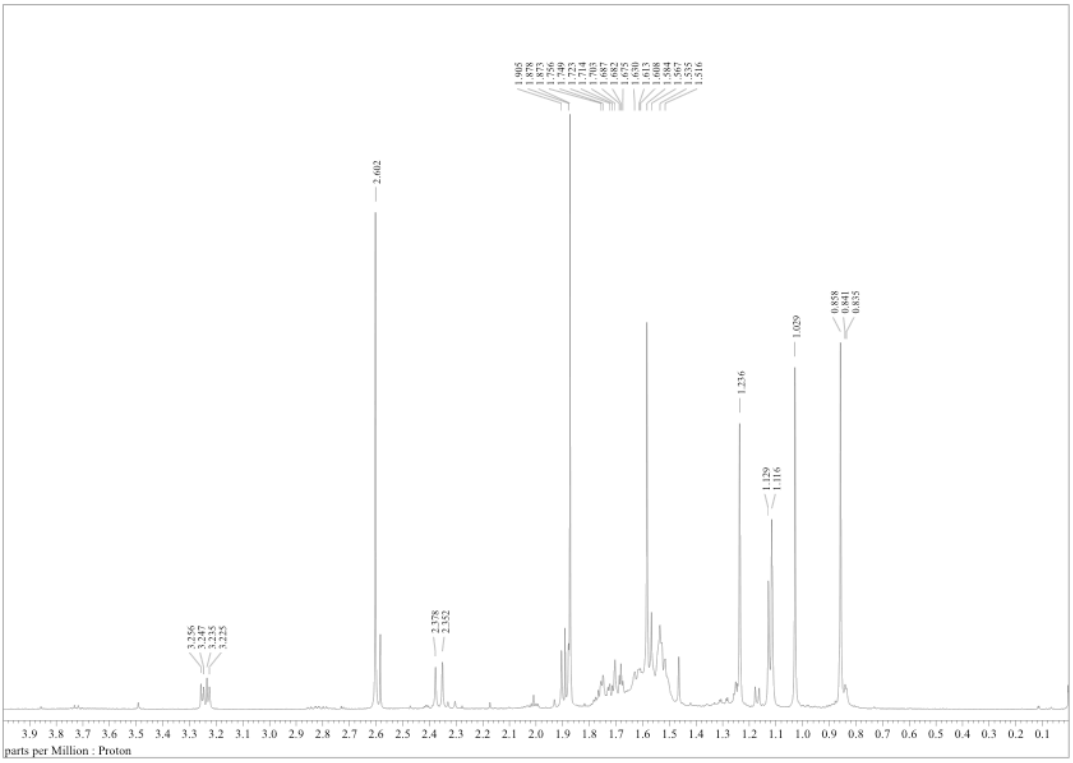

Supplementary Figure 35. $^{1}$H NMR spectrum of **8**. See Supplementary Table 12 for the NMR data.

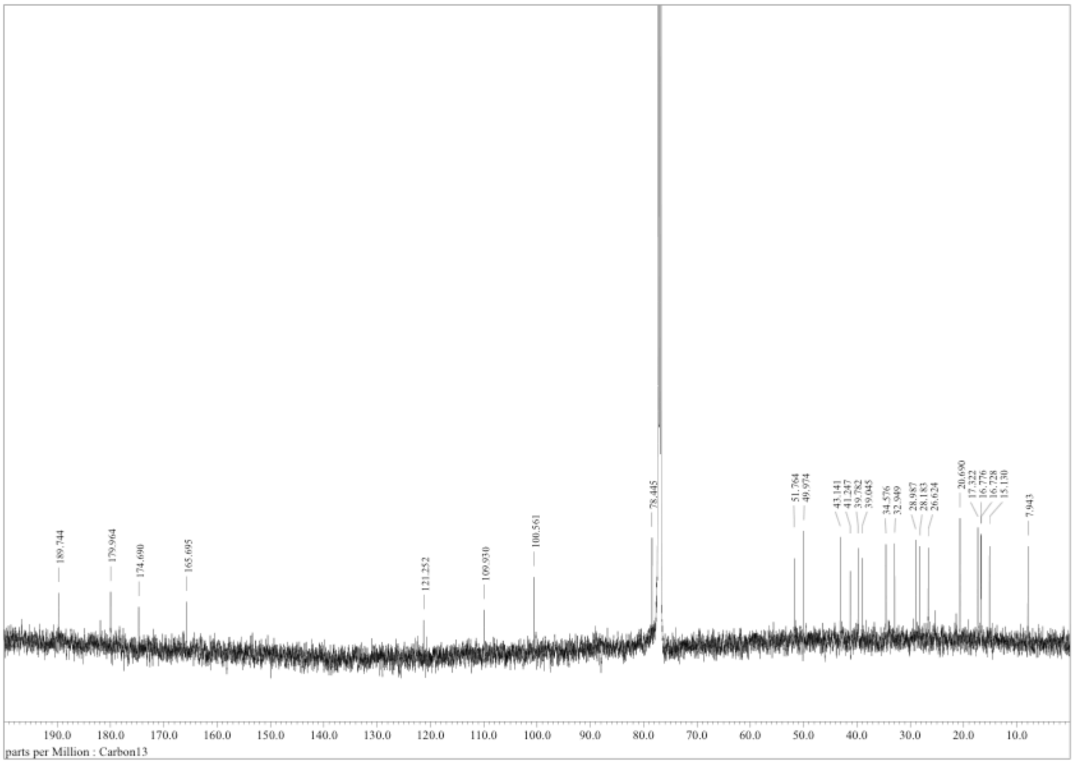

Supplementary Figure 36. $^{13}$C NMR spectrum of **8**. See Supplementary Table 12 for the NMR data.

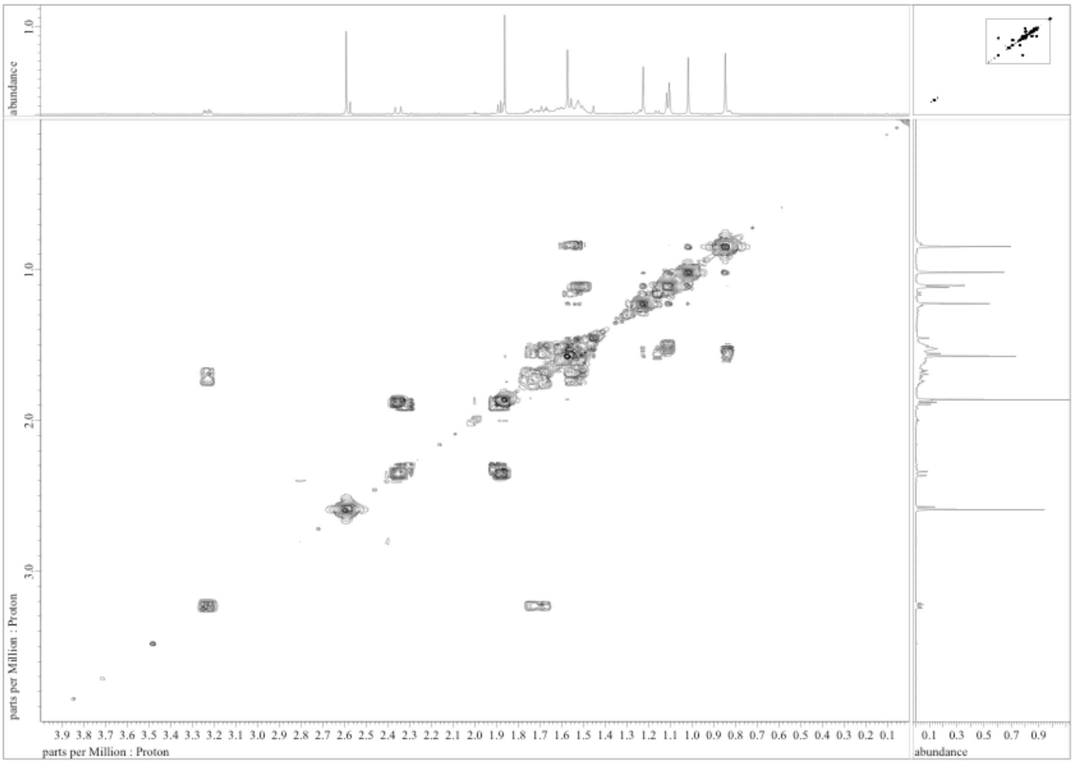


Supplementary Figure 37. $^{1}H$-$^{1}H$ COSY spectrum of **8**. See Supplementary Table 12 for the NMR data.

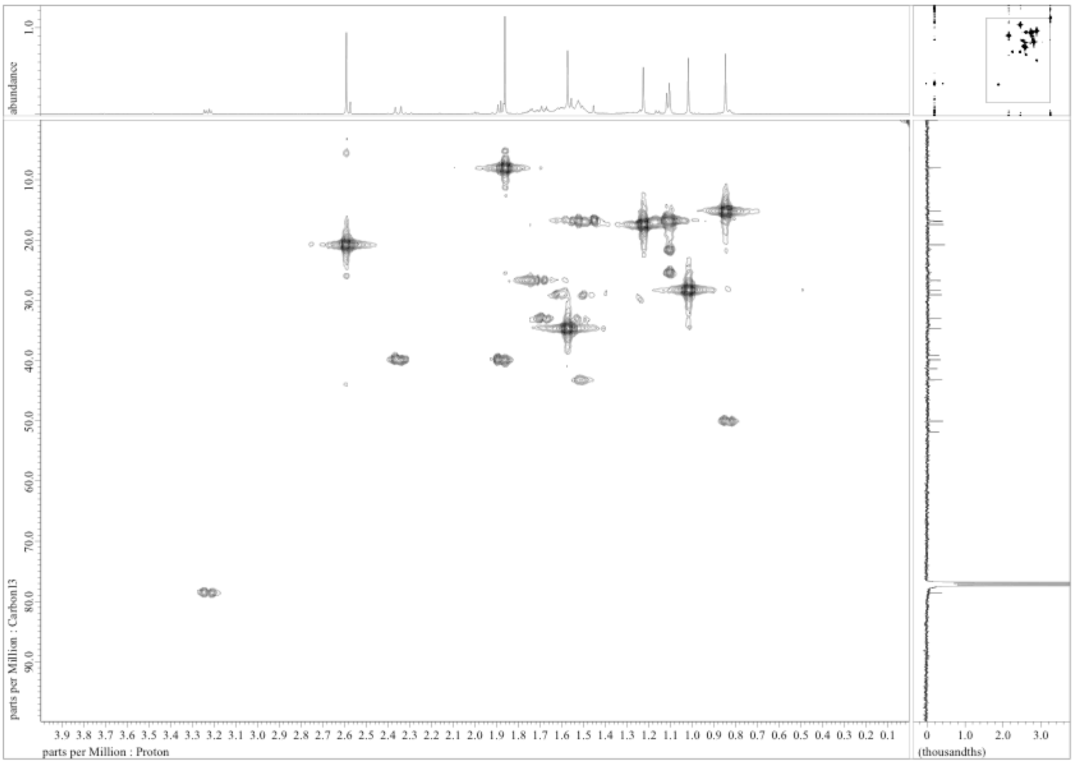


Supplementary Figure 38. HMQC spectrum of **8**. See Supplementary Table 12 for the NMR data.

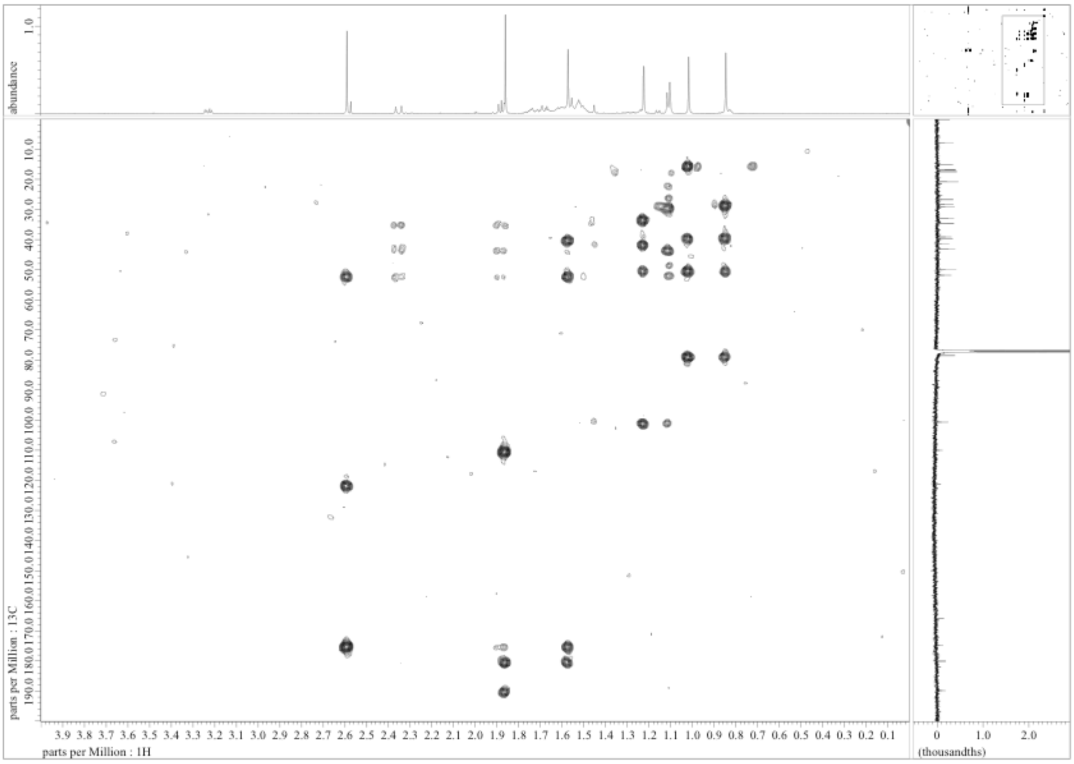


Supplementary Figure 39. HMBC spectrum of **8**. See Supplementary Table 12 for the NMR data.

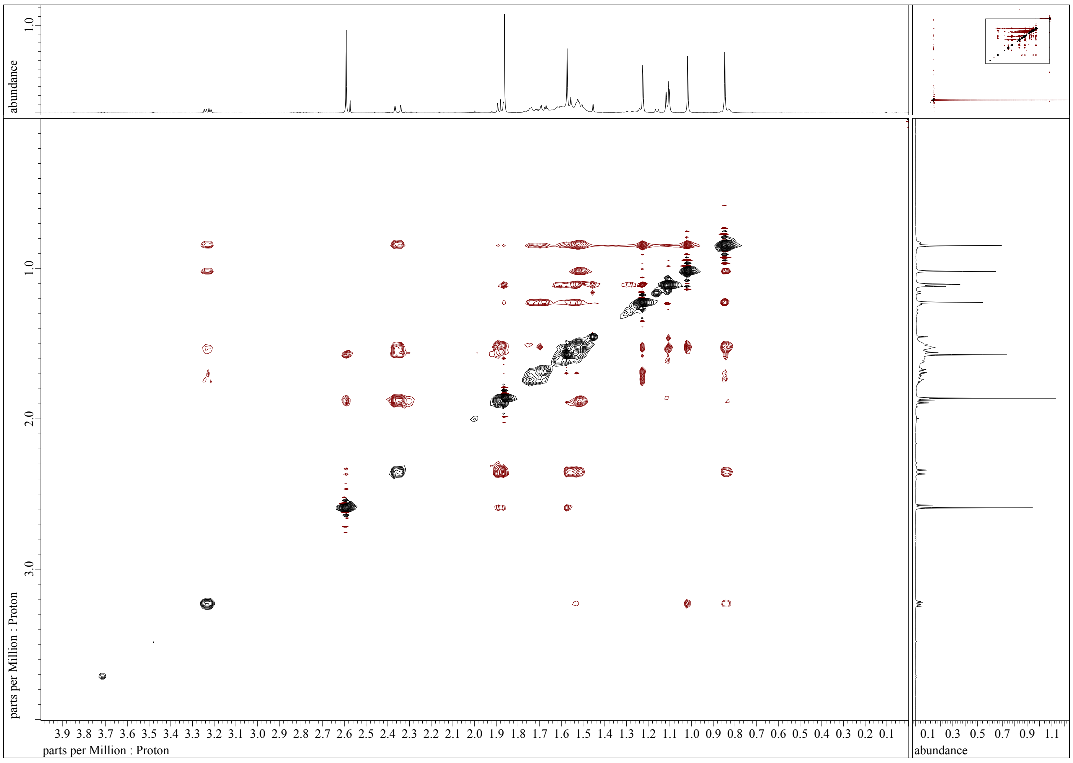


Supplementary Figure 40. NOESY spectrum of **8**. See Supplementary Table 12 for the NMR data.

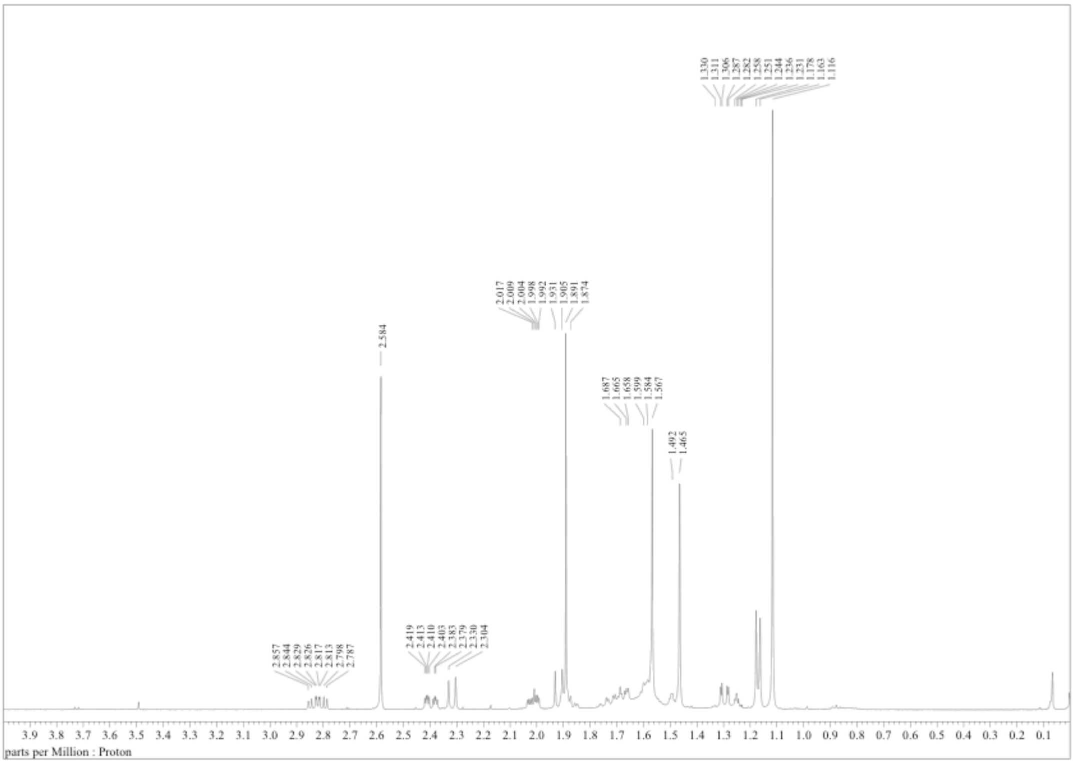

Supplementary Figure 41. $^{1}$H NMR spectrum of **10**. See Supplementary Table 13 for the NMR data.

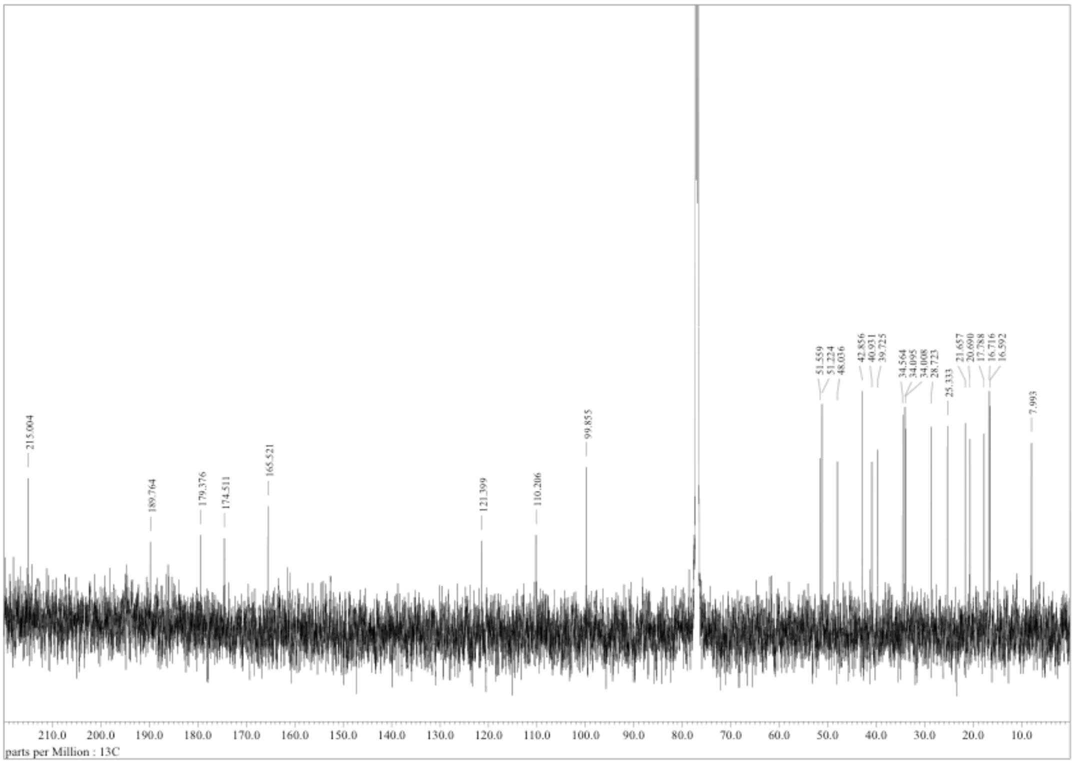

Supplementary Figure 42. $^{13}$C NMR spectrum of **10**. See Supplementary Table 13 for the NMR data.

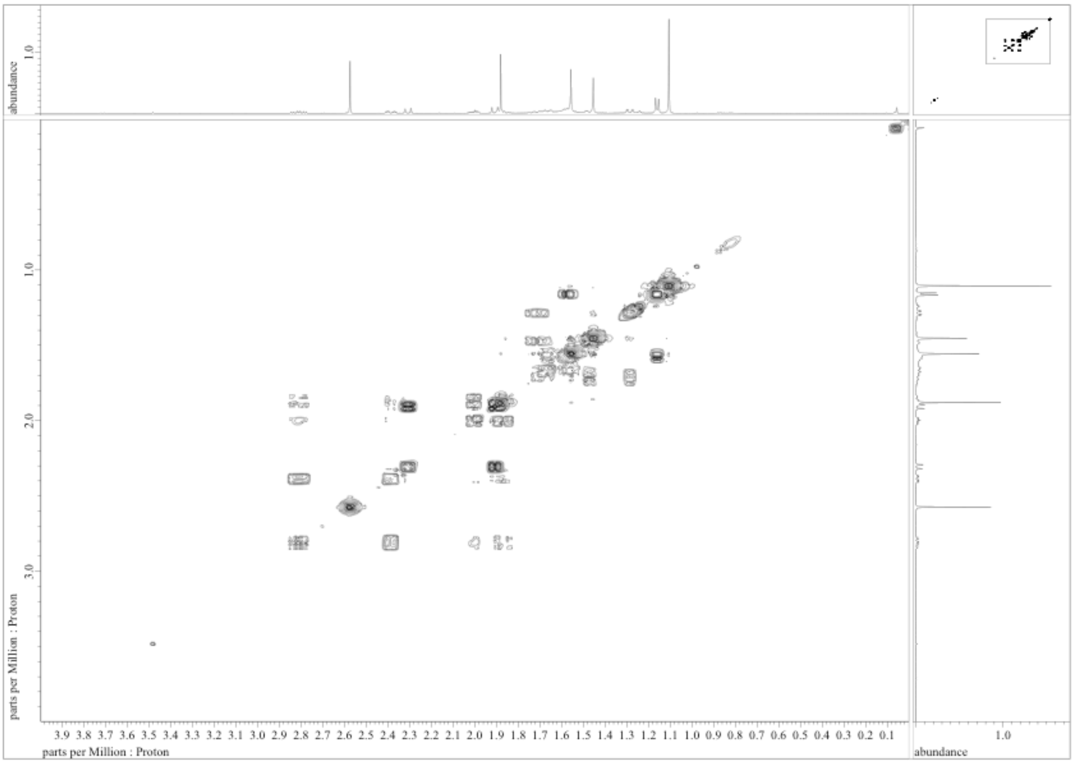


Supplementary Figure 43. $^{1}H$-$^{1}H$ COSY spectrum of **10**. See Supplementary Table 13 for the NMR data.

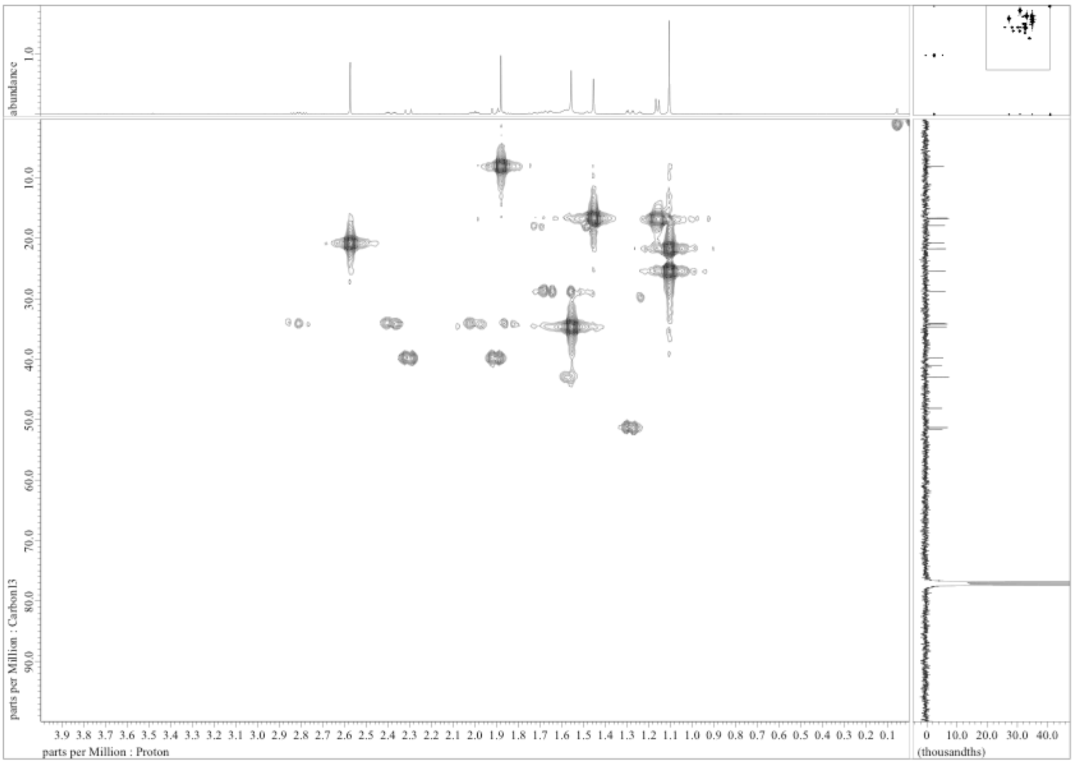


Supplementary Figure 44. HMQC spectrum of **10**. See Supplementary Table 13 for the NMR data.

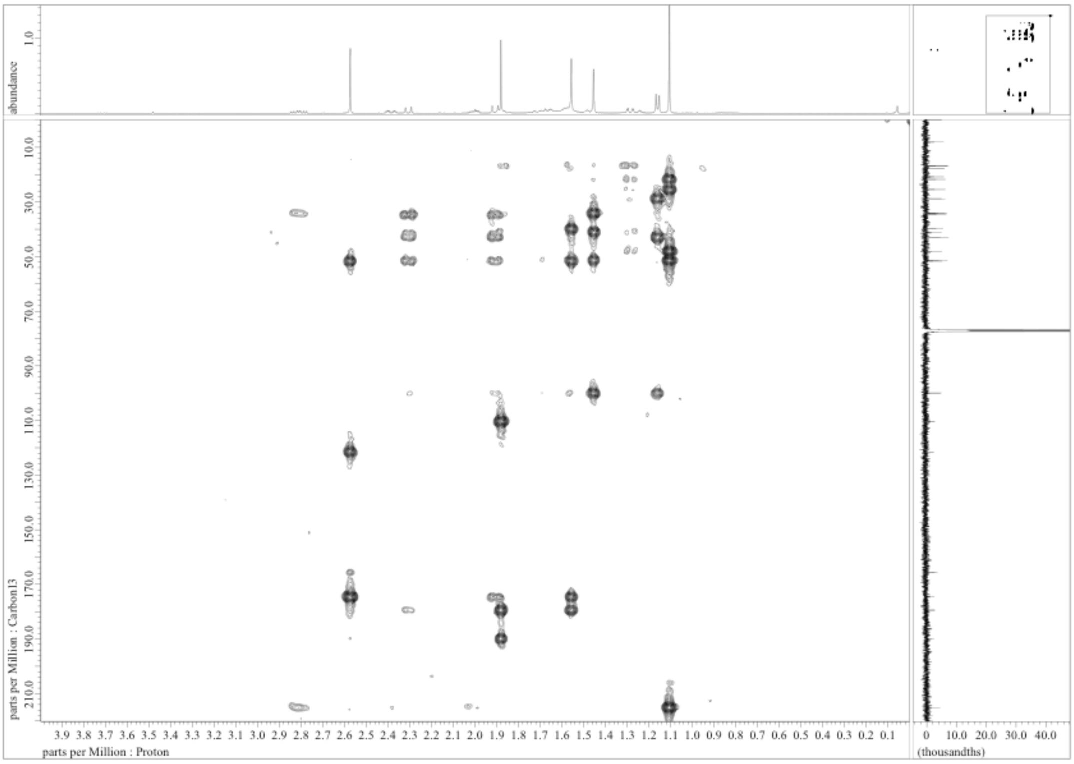


Supplementary Figure 45. HMBC spectrum of **10**. See Supplementary Table 13 for the NMR data.

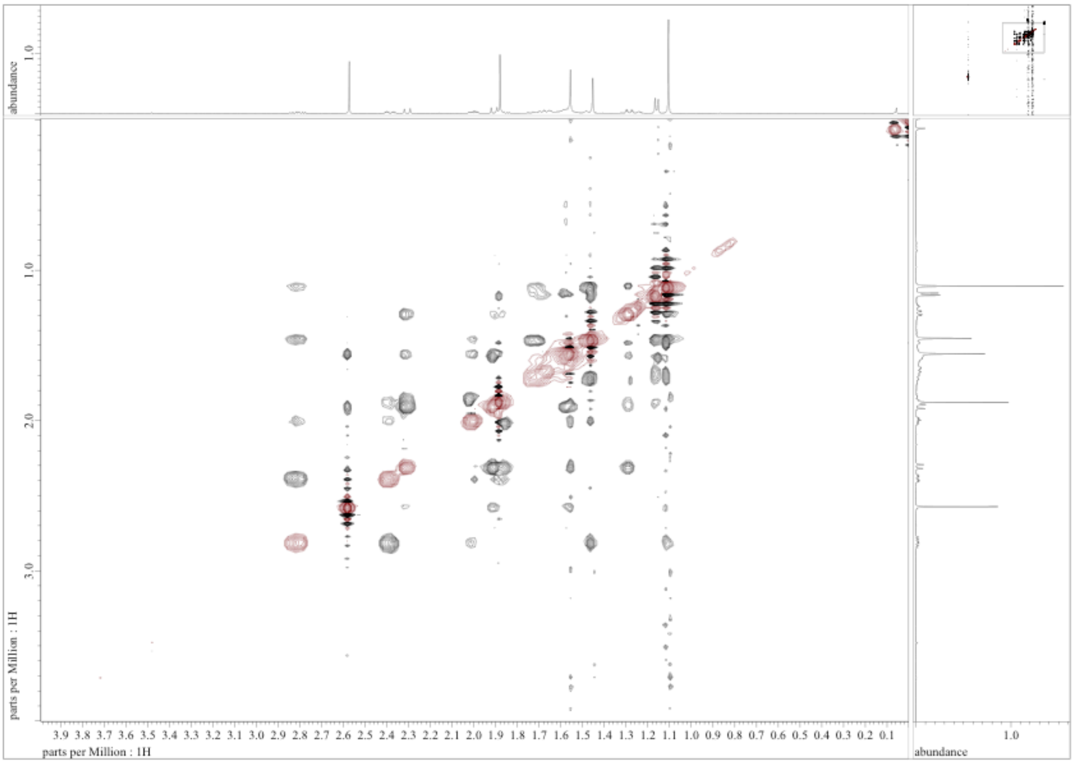


Supplementary Figure 46. NOESY spectrum of **10**. See Supplementary Table 13 for the NMR data.

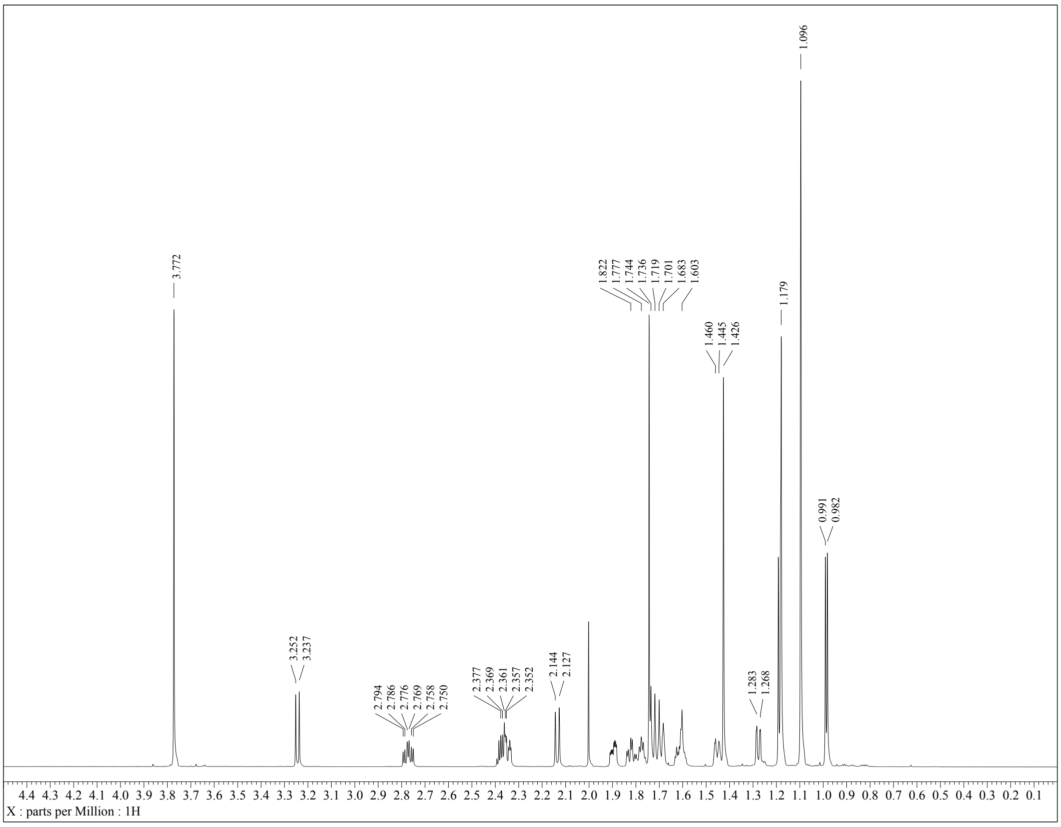


Supplementary Figure 47. $^1$H NMR spectrum of **11**. See Supplementary Table 14 for the NMR data.

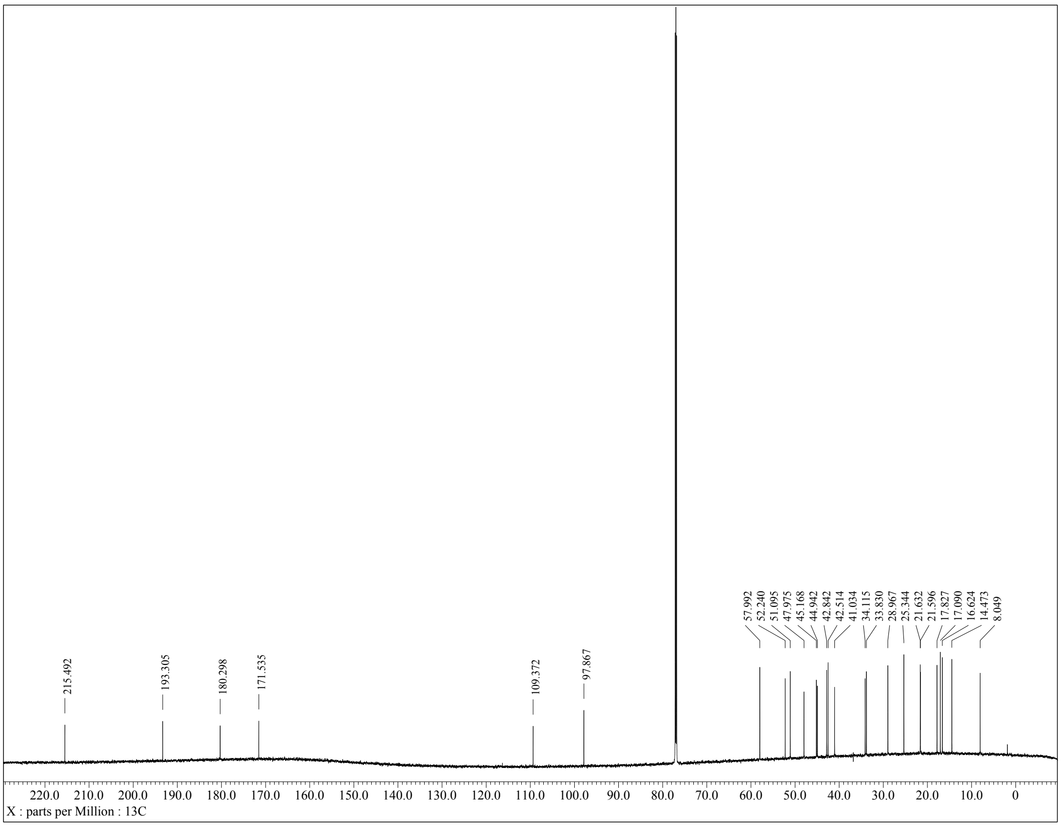


Supplementary Figure 48. $^{13}$C NMR spectrum of **11**. See Supplementary Table 14 for the NMR data.

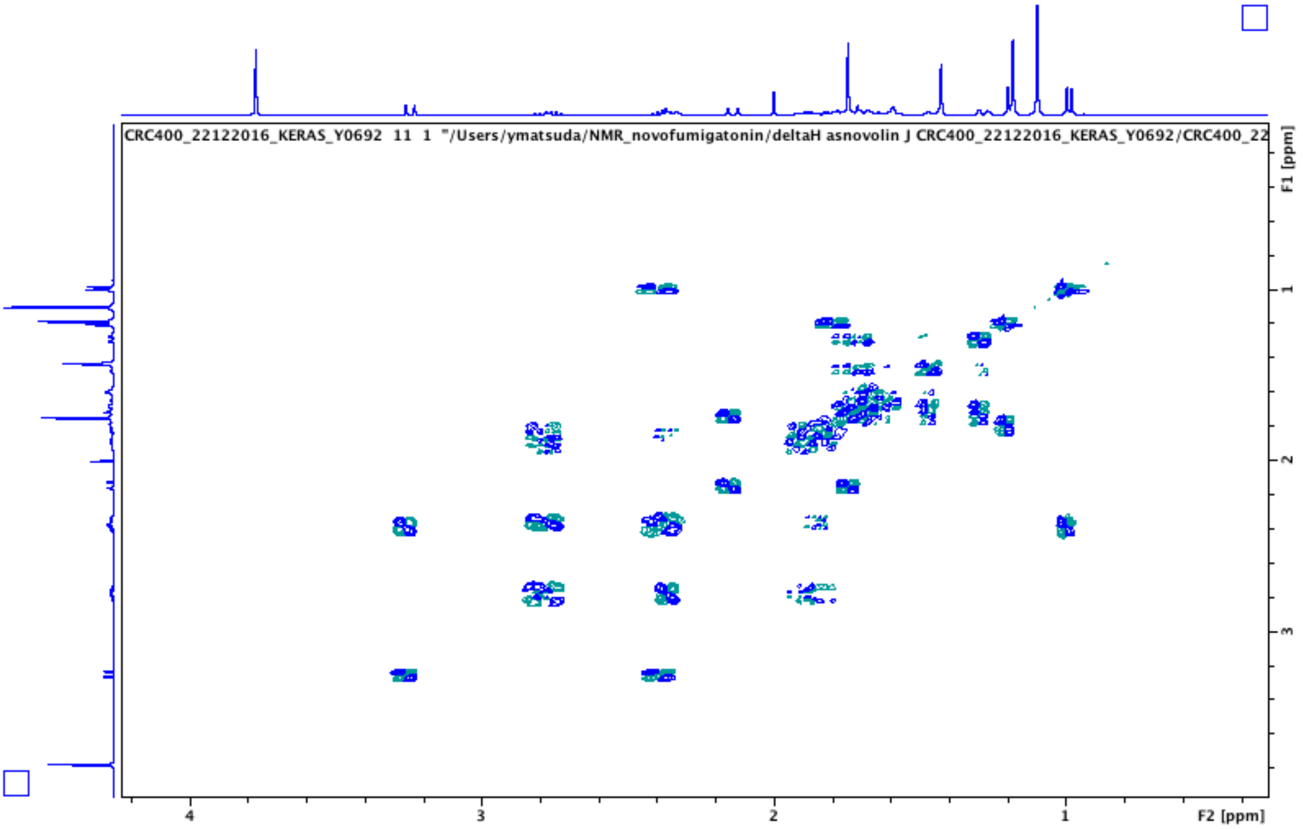


Supplementary Figure 49. $^{1}H$-$^{1}H$ DQF-COSY spectrum of **11**. See Supplementary Table 14 for the NMR data.

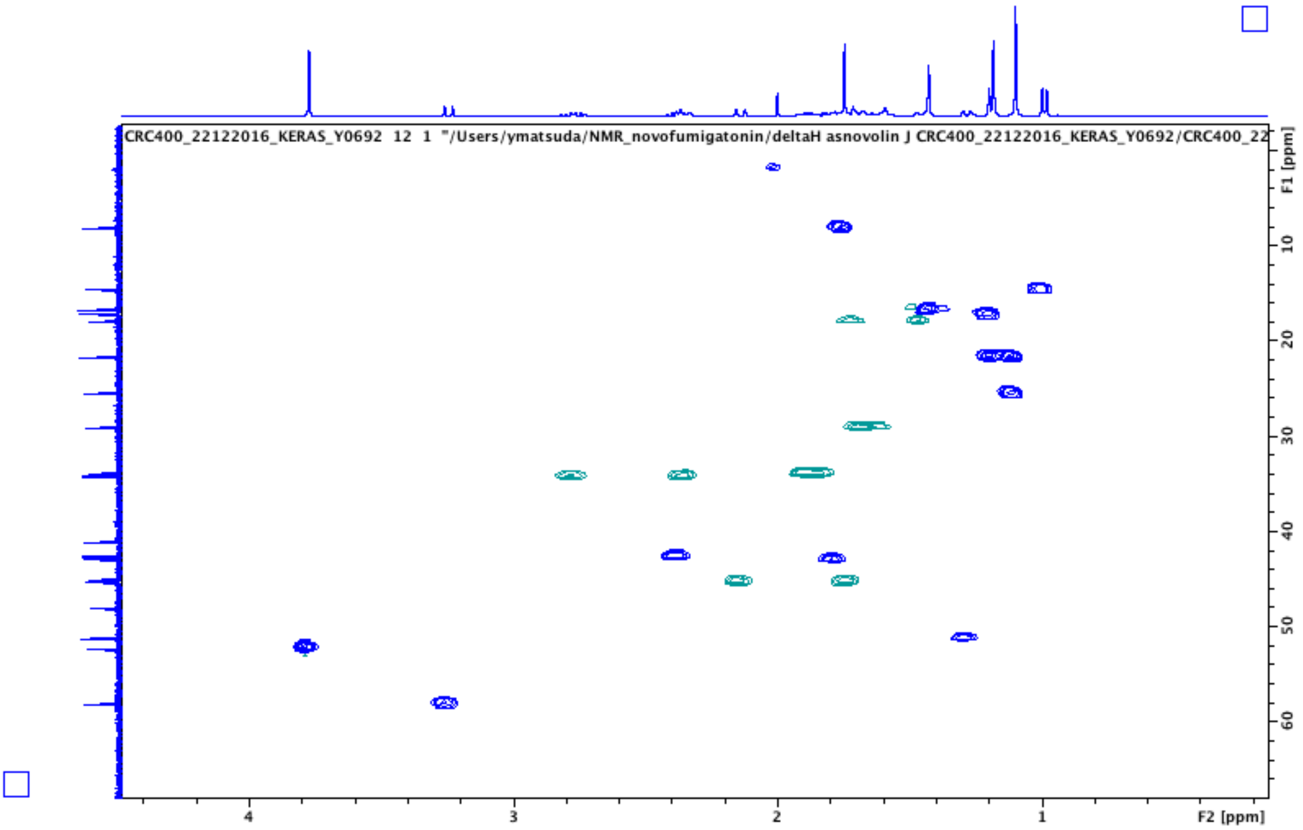


Supplementary Figure 50. HSQC spectrum of **11**. See Supplementary Table 14 for the NMR data.

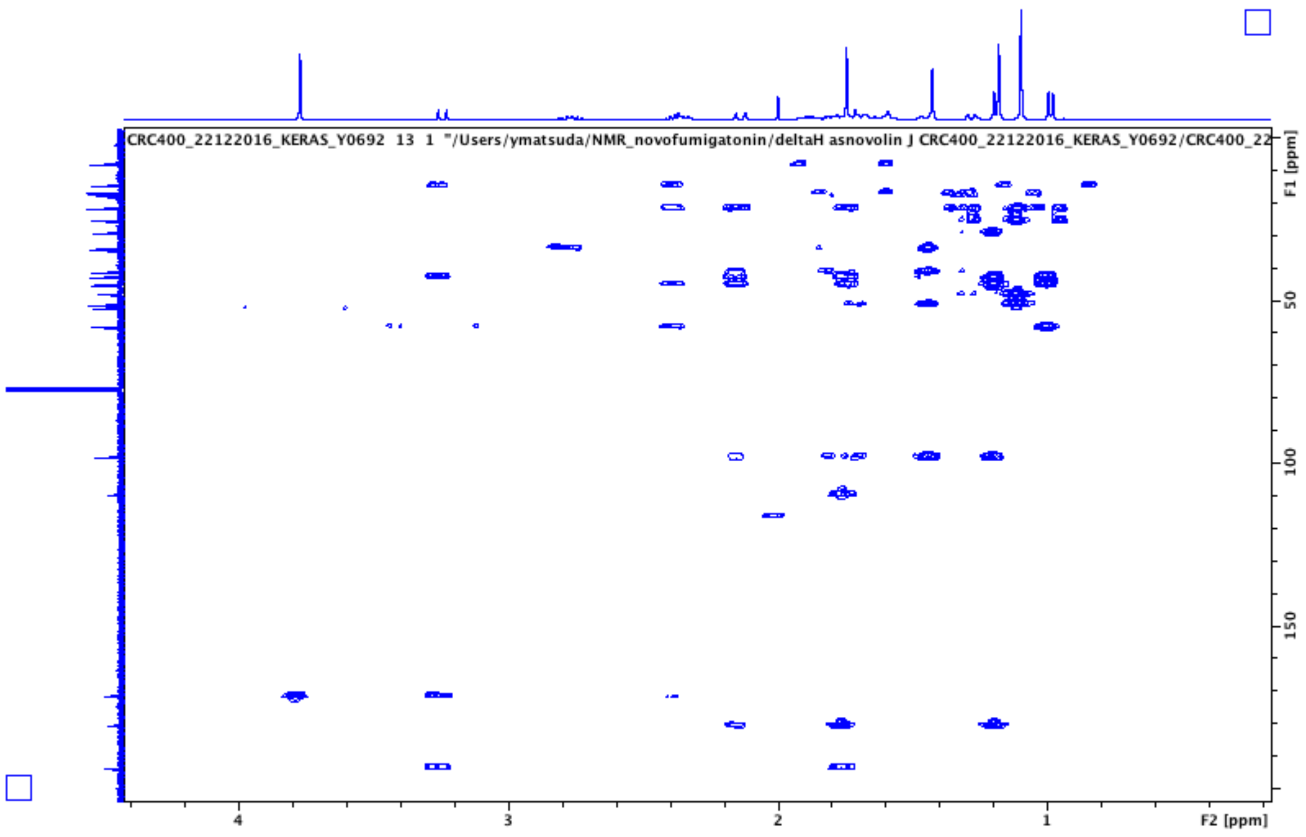


Supplementary Figure 51. HMBC spectrum of **11**. See Supplementary Table 14 for the NMR data.

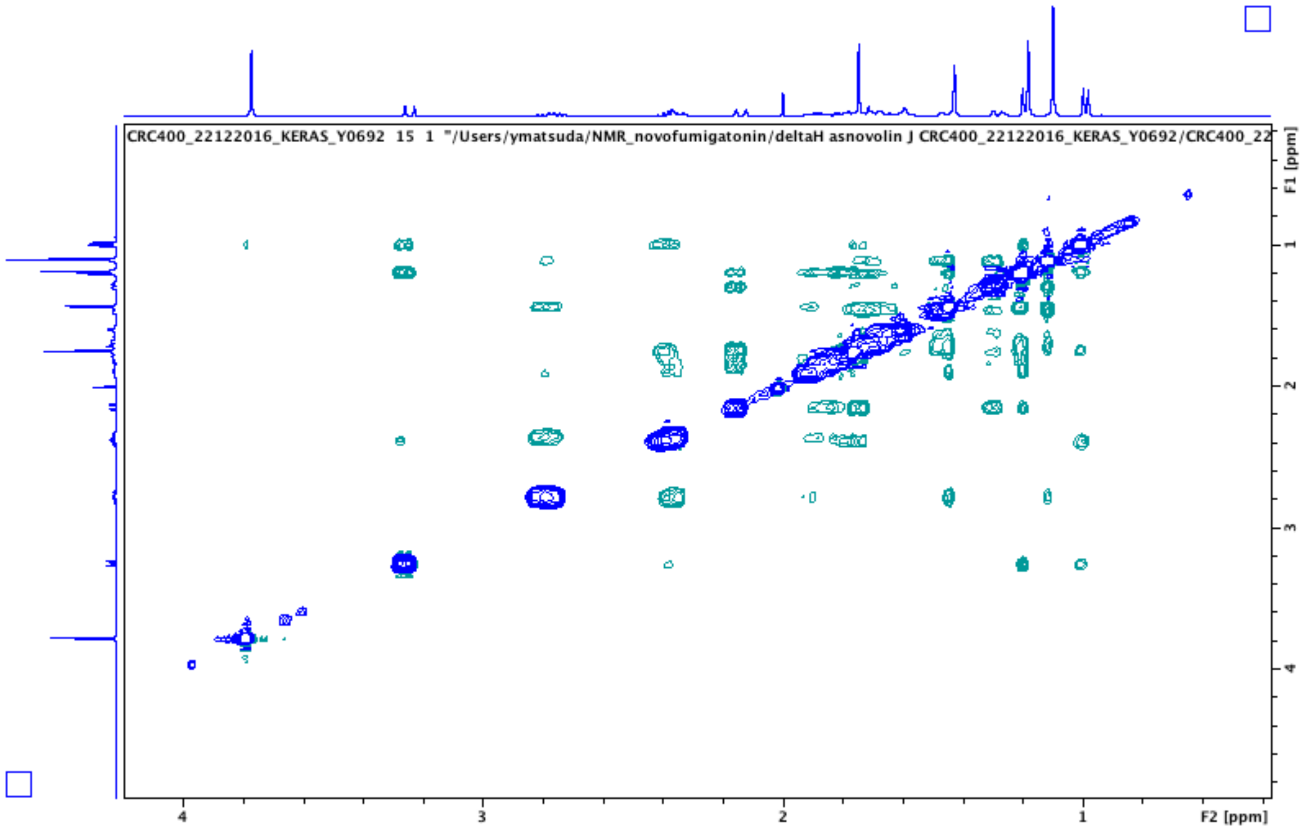


Supplementary Figure 52. NOESY spectrum of **11**. See Supplementary Table 14 for the NMR data.

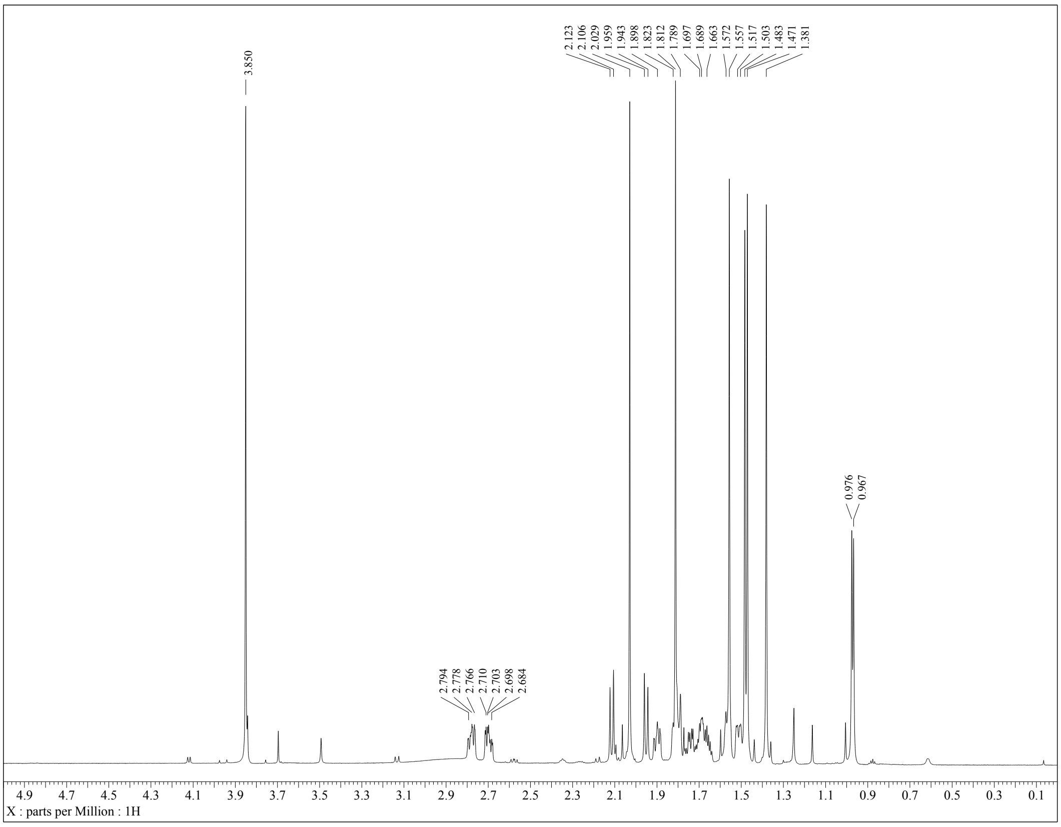


Supplementary Figure 53. $^{1}H$ NMR spectrum of **12**. See Supplementary Table 15 for the NMR data.

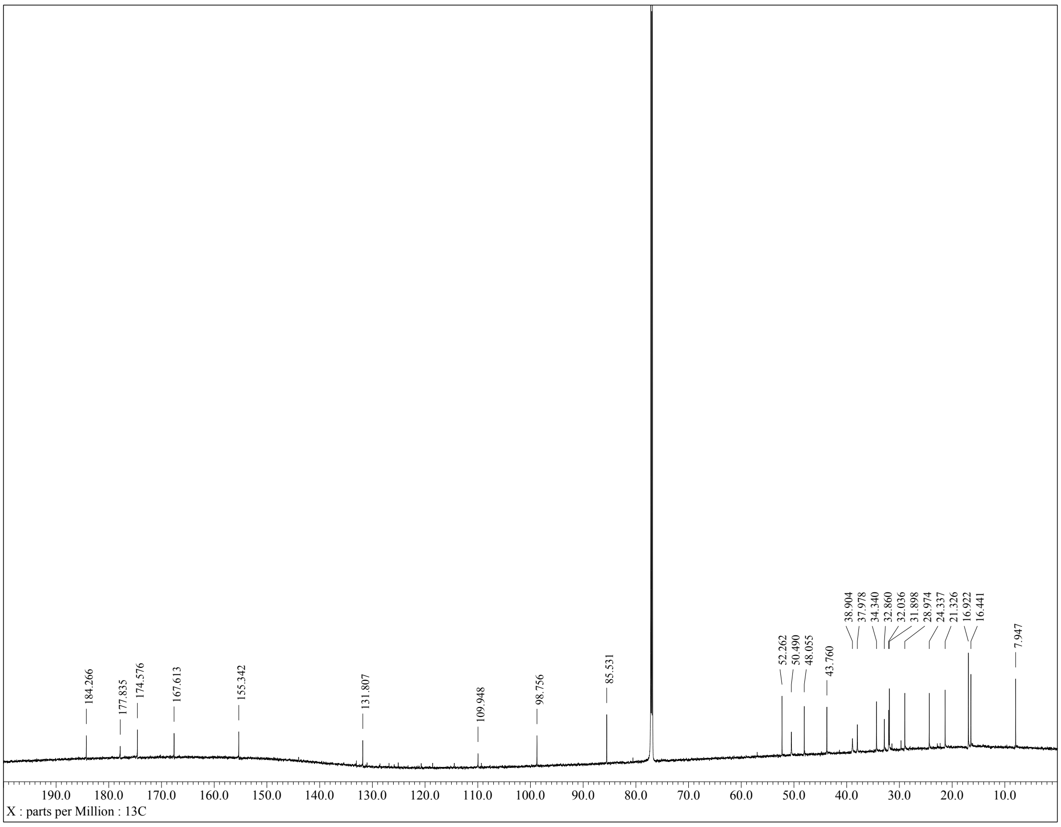


Supplementary Figure 54. $^{13}C$ NMR spectrum of **12**. See Supplementary Table 15 for the NMR data.

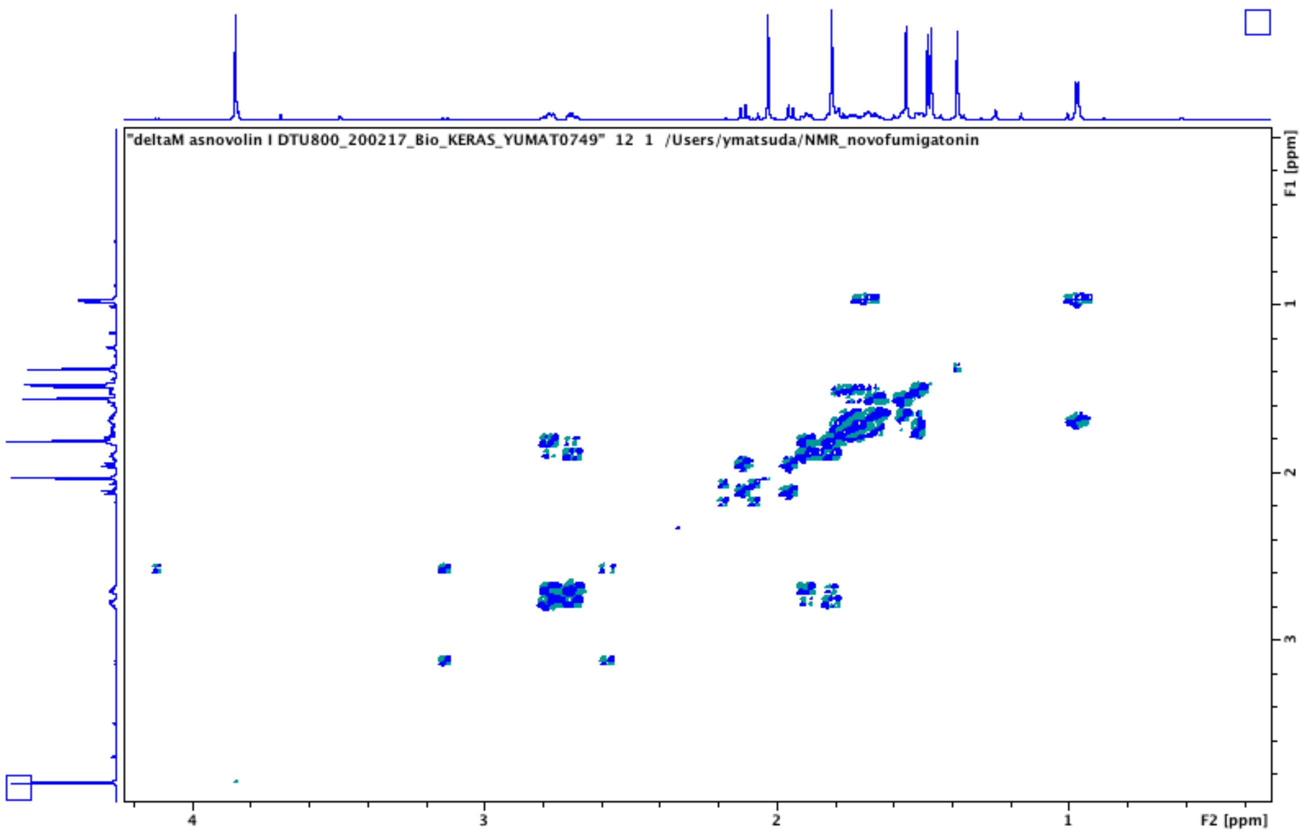


Supplementary Figure 55. $^{1}$H-$^{1}$H DQF-COSY spectrum of **12**. See Supplementary Table 15 for the NMR data.

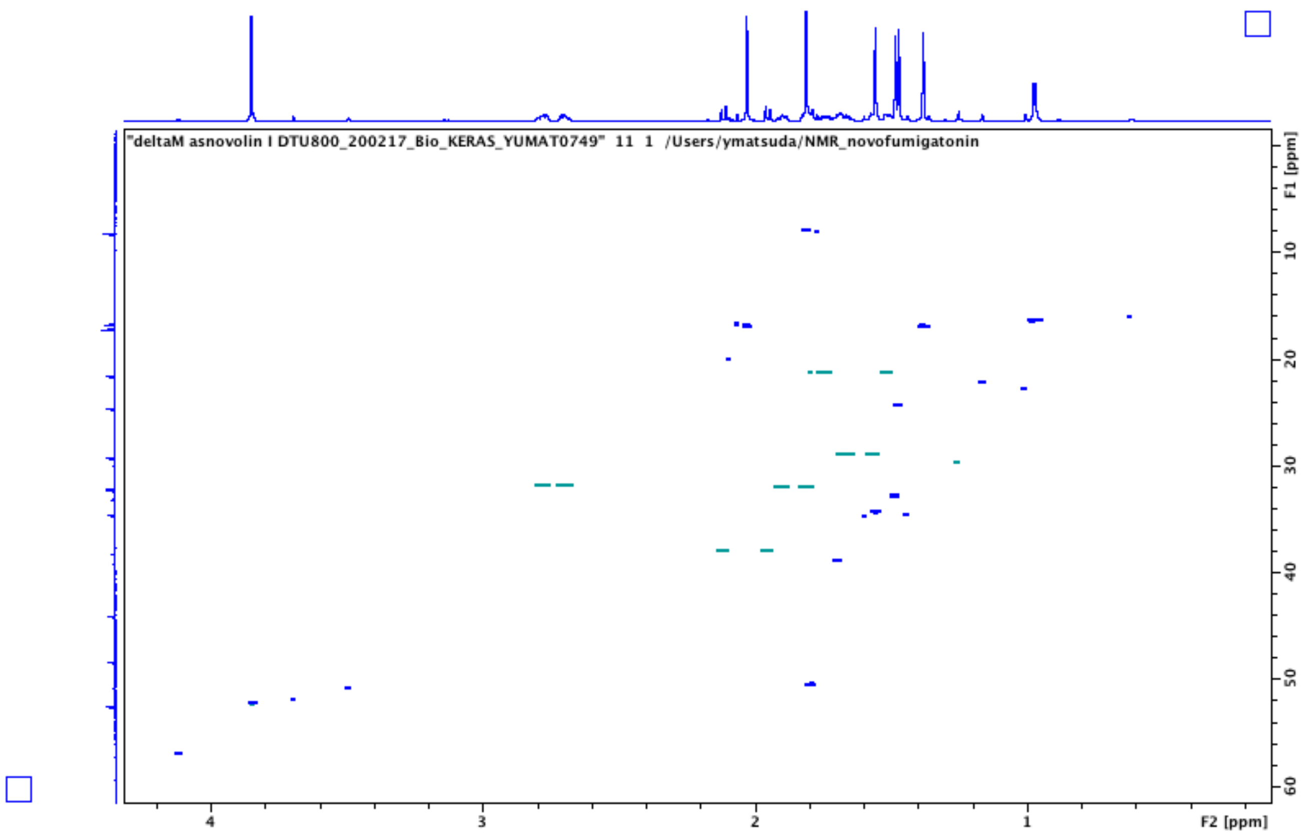


Supplementary Figure 56. HSQC spectrum of **12**. See Supplementary Table 15 for the NMR data.

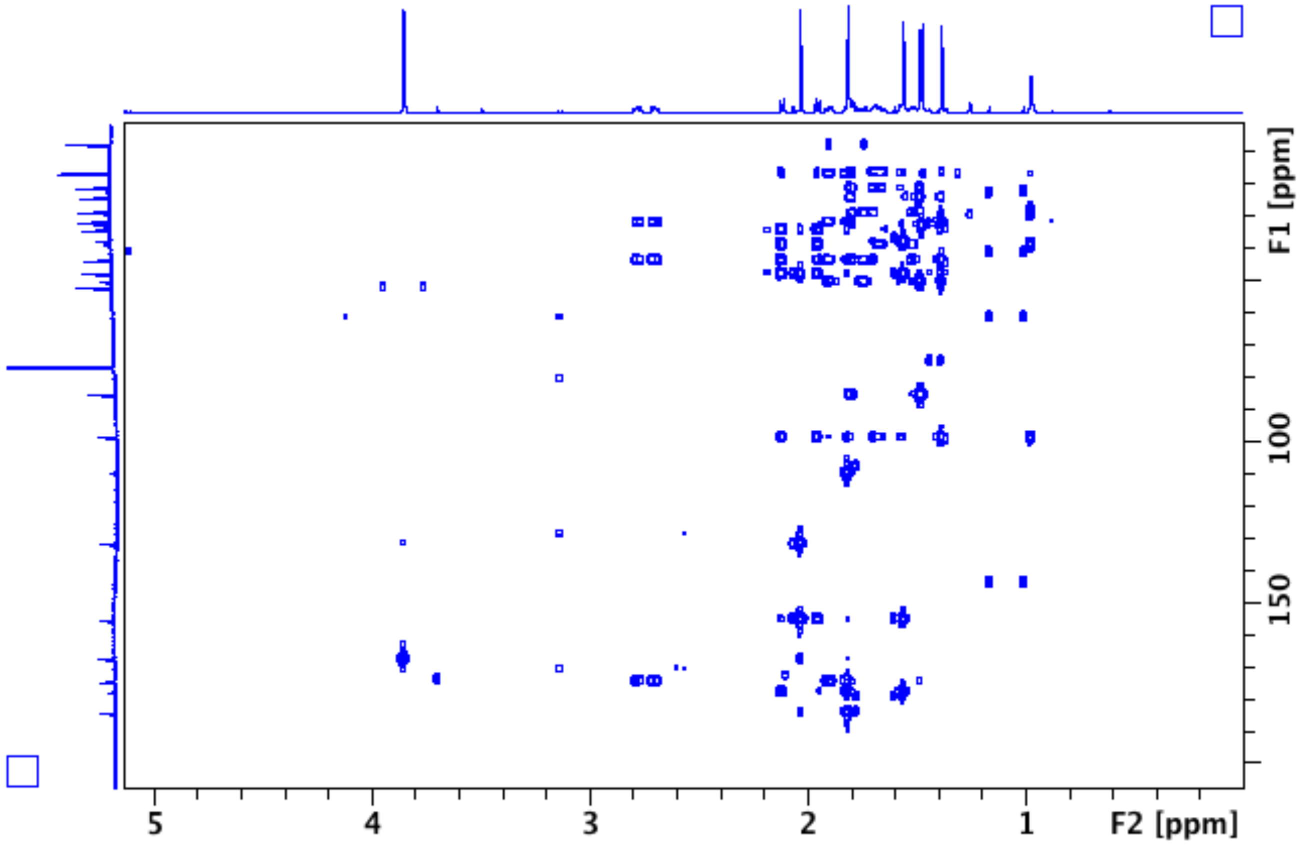


Supplementary Figure 57. HMBC spectrum of **12**. See Supplementary Table 15 for the NMR data.

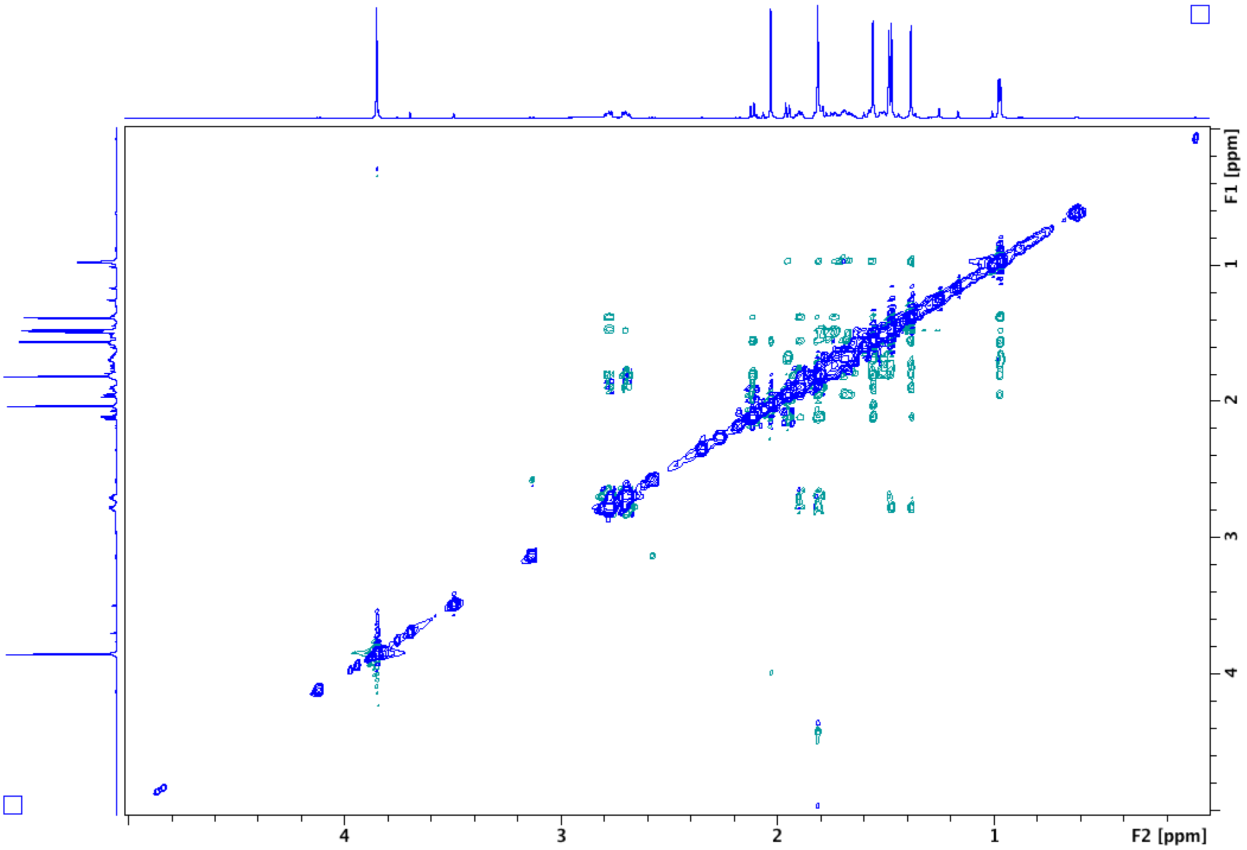


Supplementary Figure 58. NOESY spectrum of **12**. See Supplementary Table 15 for the NMR data.

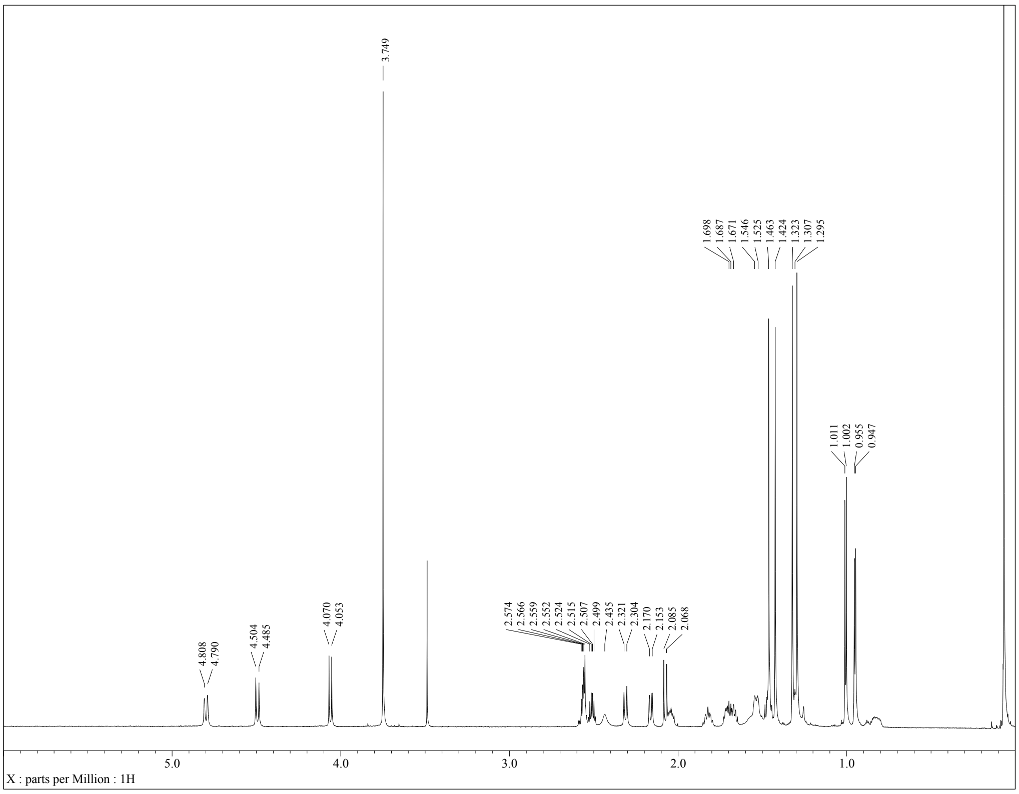


Supplementary Figure 59. $^{1}H$ NMR spectrum of **14** in $CDCl_3$. See Supplementary Table 16 for the NMR data.

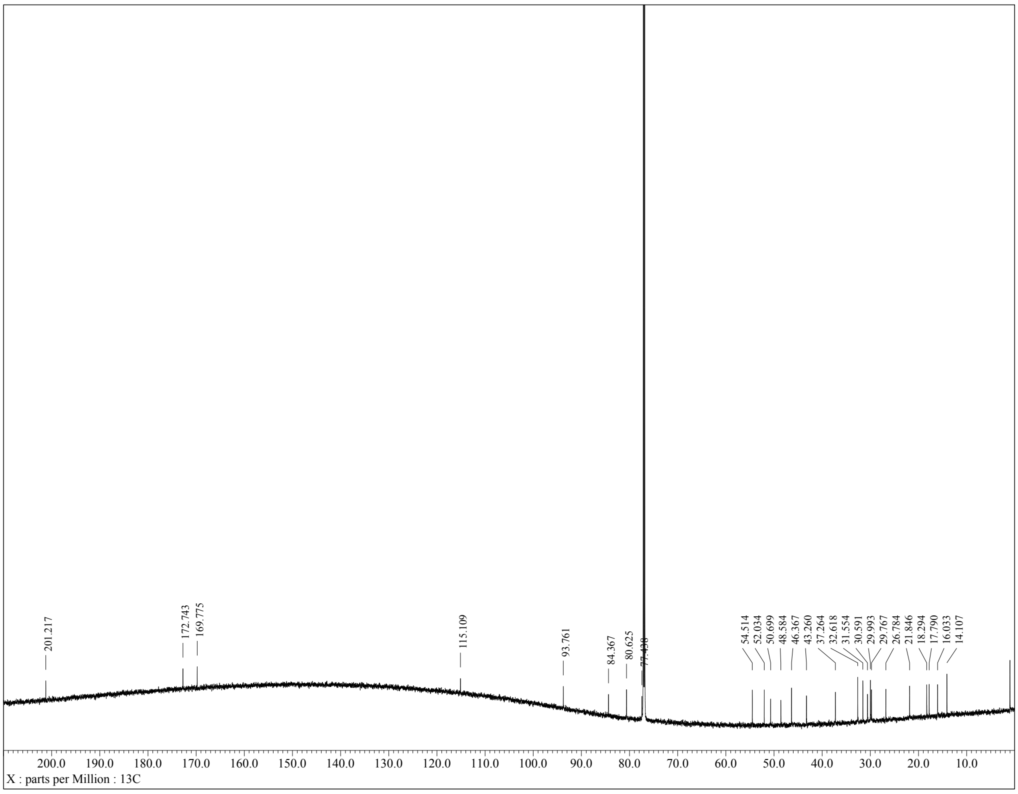


Supplementary Figure 60. $^{13}C$ NMR spectrum of **14** in $CDCl_3$. See Supplementary Table 16 for the NMR data.

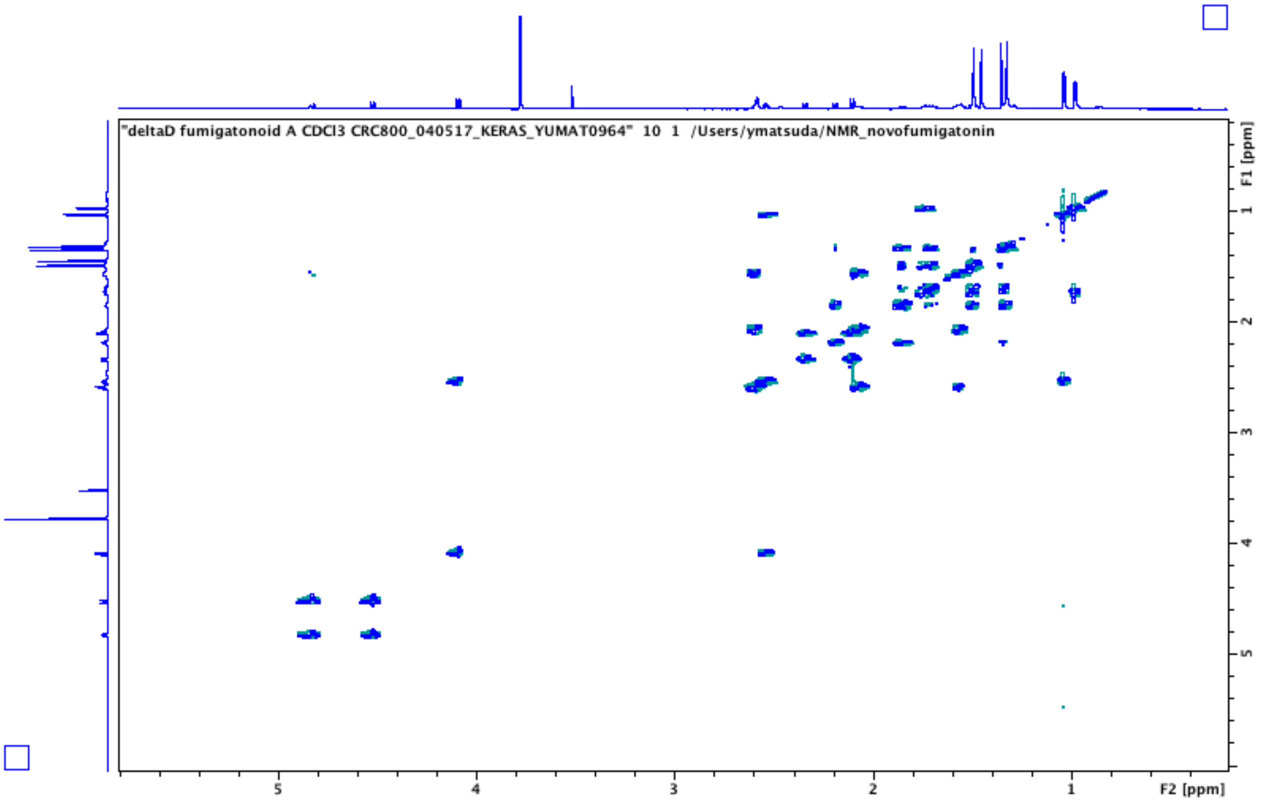


Supplementary Figure 61. $^{1}$H-$^{1}$H DQF-COSY spectrum of **14** in $CDCl_3$. See Supplementary Table 16 for the NMR data.

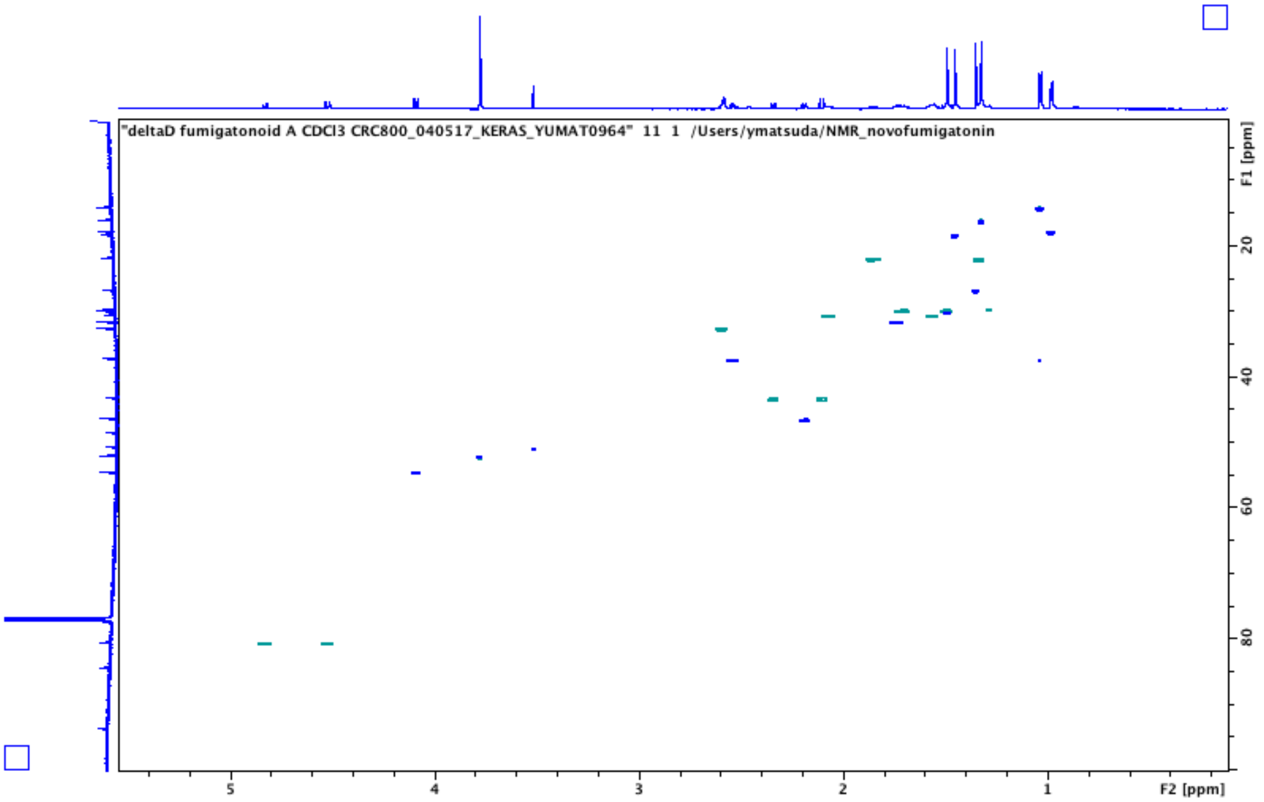


Supplementary Figure 62. HSQC spectrum of **14** in $CDCl_3$. See Supplementary Table 16 for the NMR data.

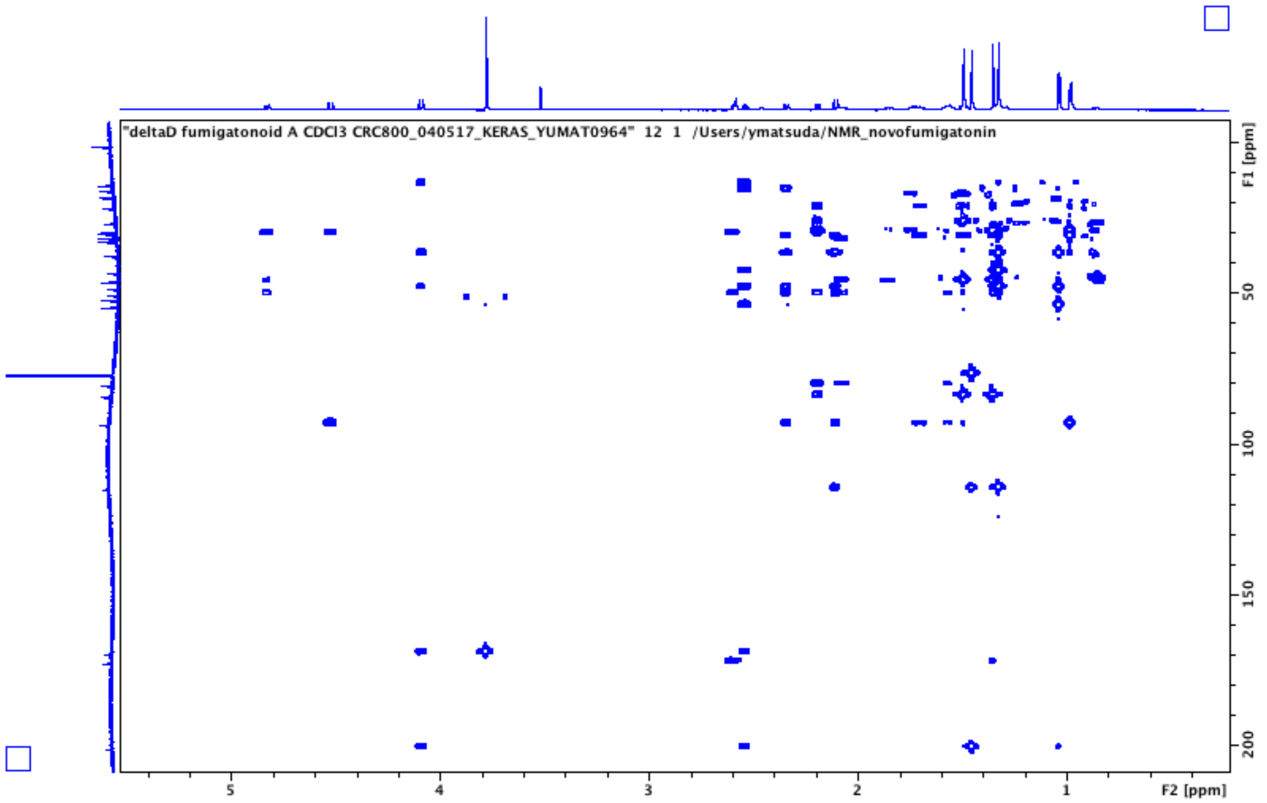


Supplementary Figure 63. HMBC spectrum of **14** in $CDCl_3$. See Supplementary Table 16 for the NMR data.

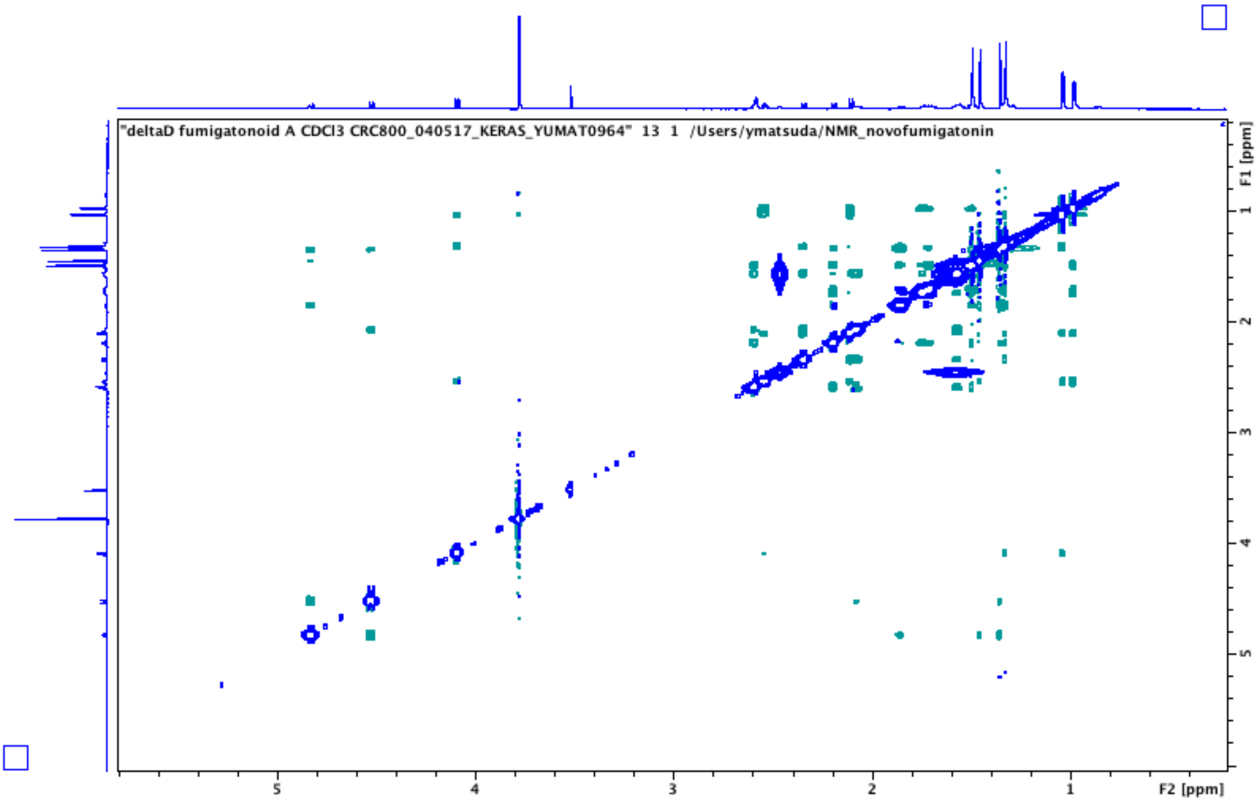


Supplementary Figure 64. NOESY spectrum of **14** in $CDCl_3$. See Supplementary Table 16 for the NMR data.

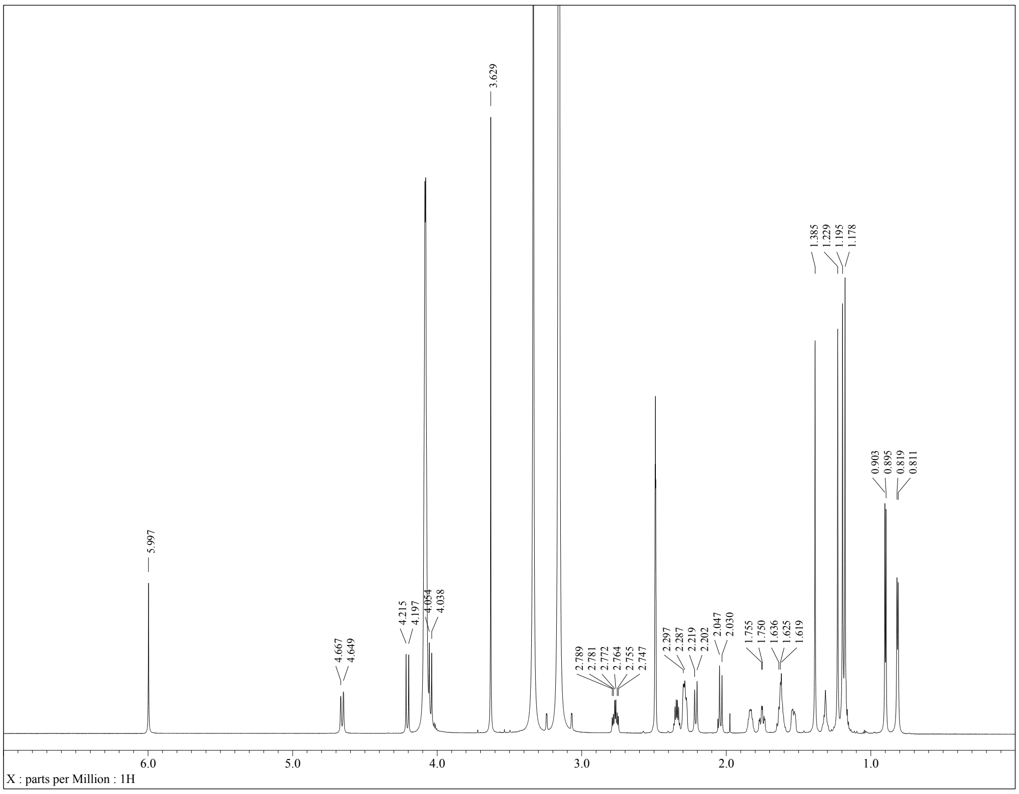


Supplementary Figure 65. $^{1}H$ NMR spectrum of **14** in DMSO-$d_6$. See Supplementary Table 17 for the NMR data.

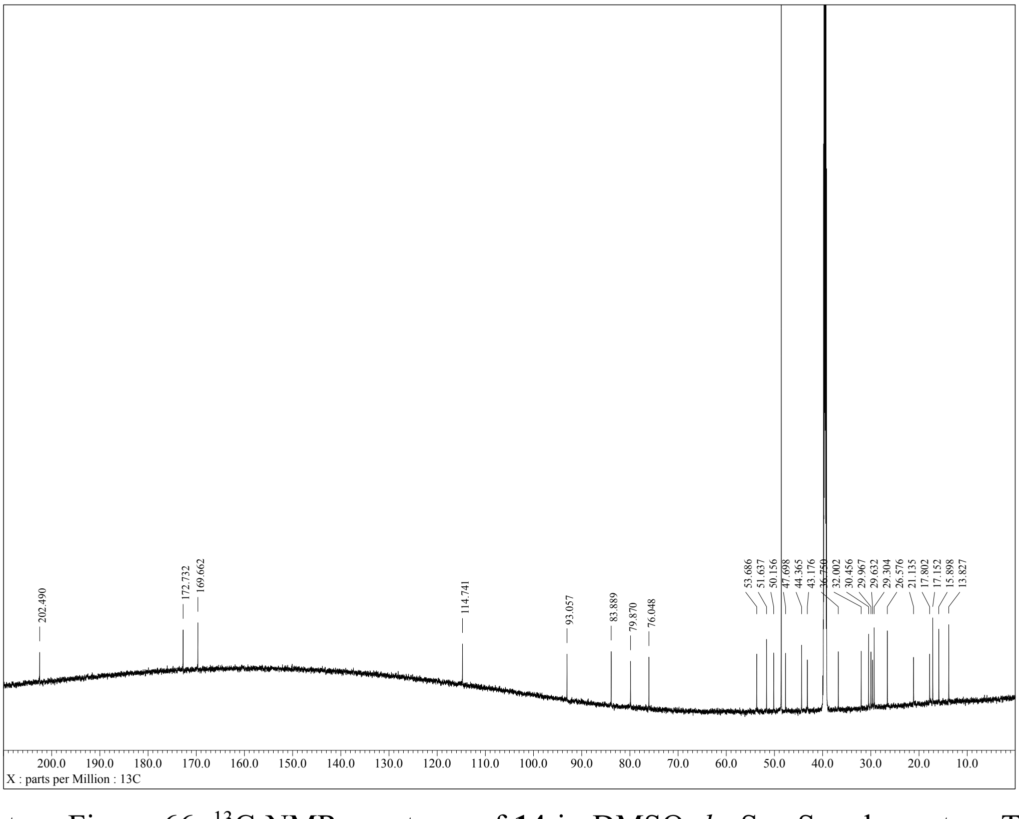


Supplementary Figure 66. $^{13}C$ NMR spectrum of **14** in DMSO-$d_6$. See Supplementary Table 17 for the NMR data.

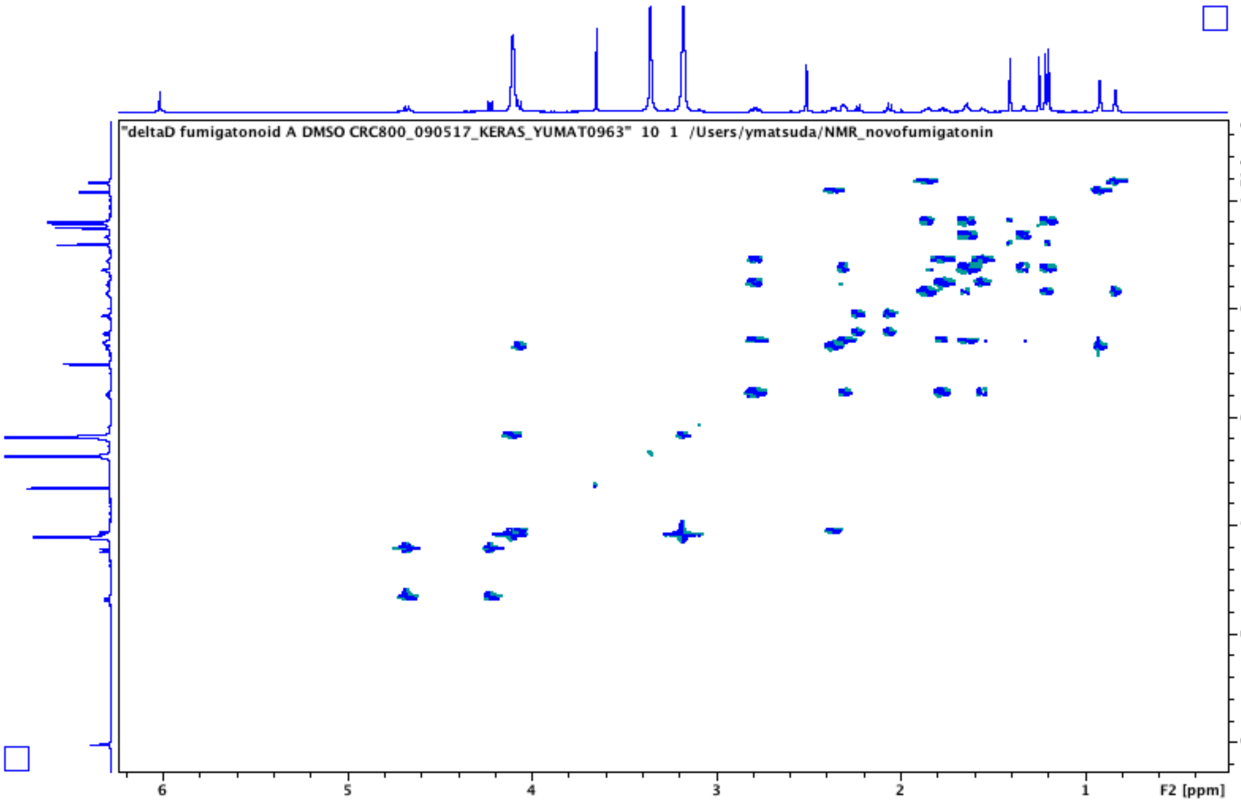


Supplementary Figure 67. $^{1}$H-$^{1}$H DQF-COSY spectrum of **14** in DMSO-$d_6$. See Supplementary Table 17 for the NMR data.

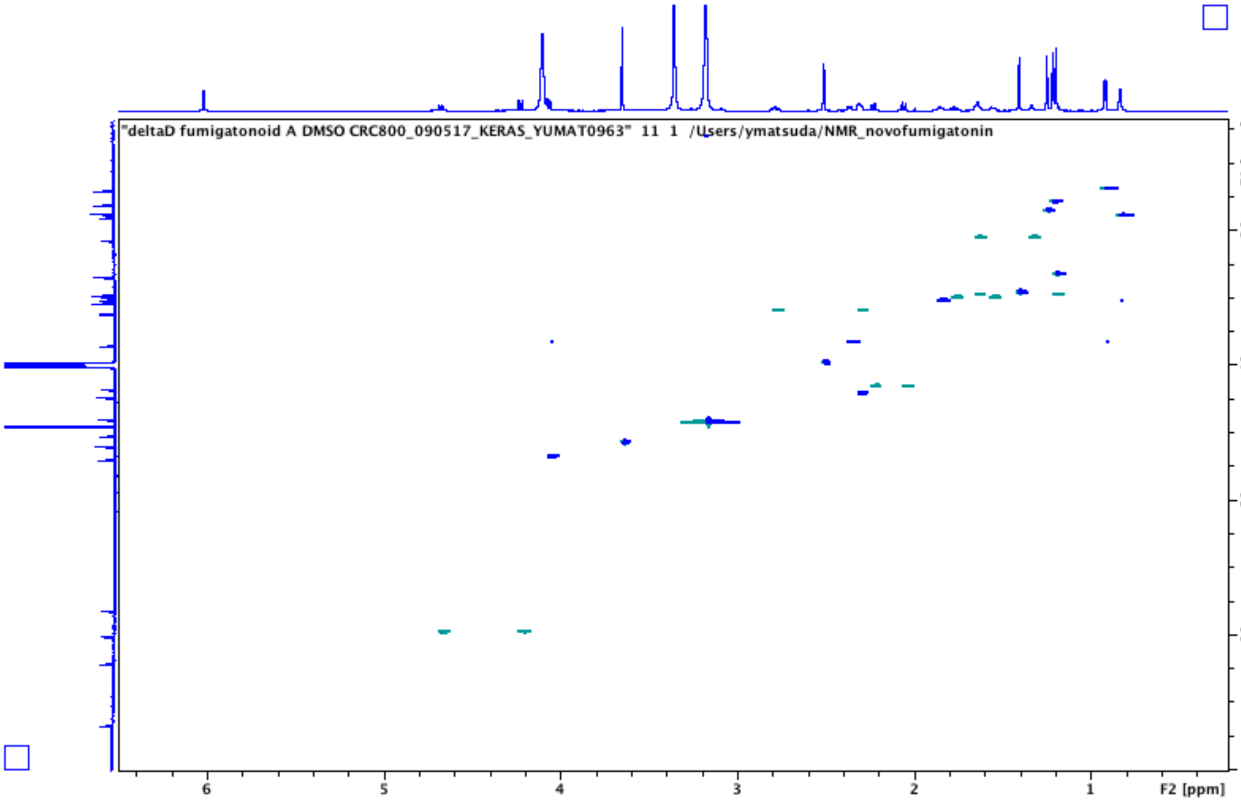


Supplementary Figure 68. HSQC spectrum of **14** in DMSO-$d_6$. See Supplementary Table 17 for the NMR data.

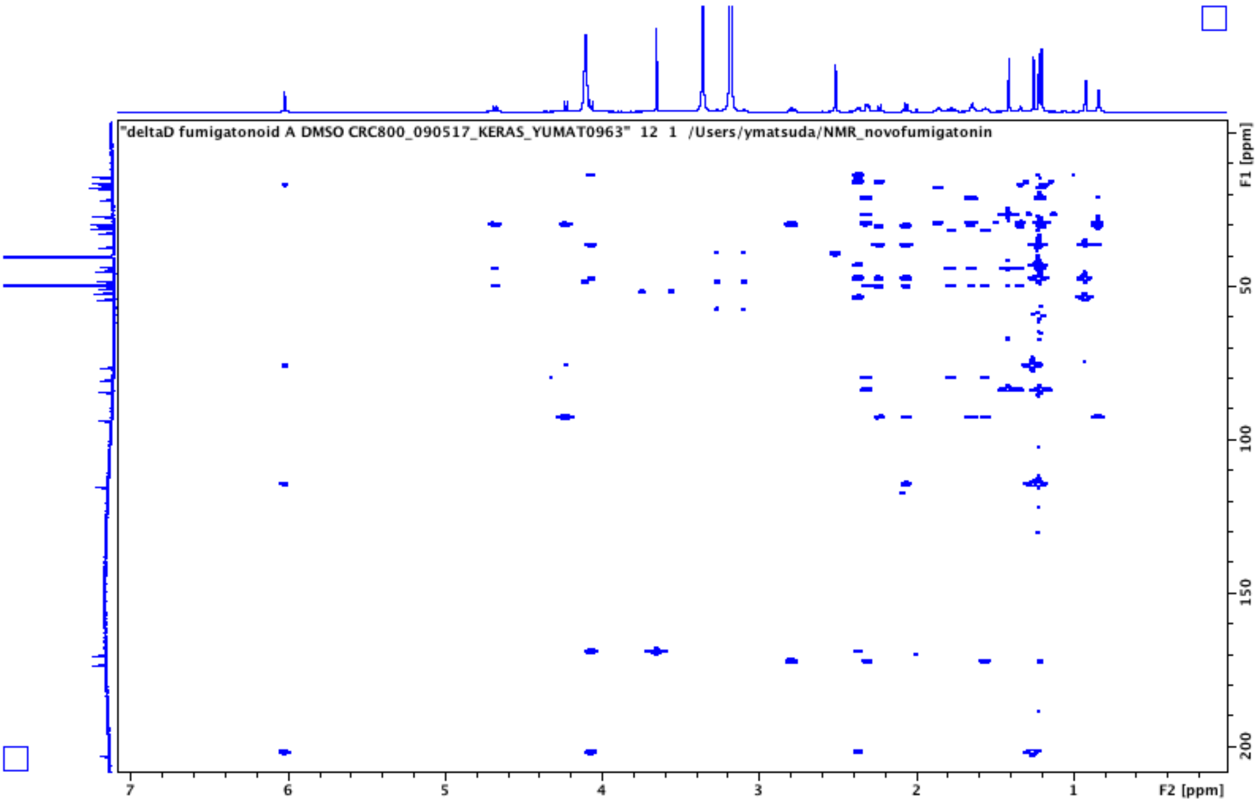


Supplementary Figure 69. HMBC spectrum of **14** in DMSO-$d_6$. See Supplementary Table 17 for the NMR data.

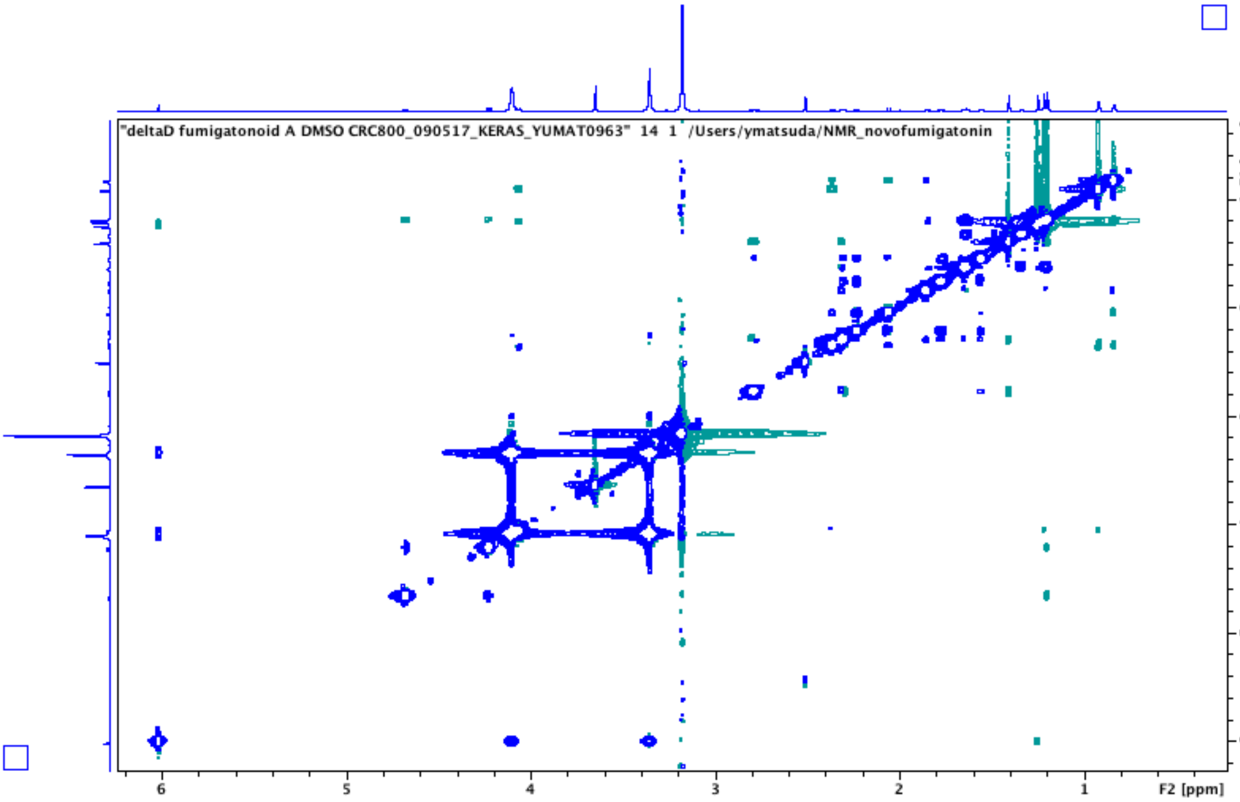


Supplementary Figure 70. NOESY spectrum of **14** in DMSO-$d_6$. See Supplementary Table 17 for the NMR data.

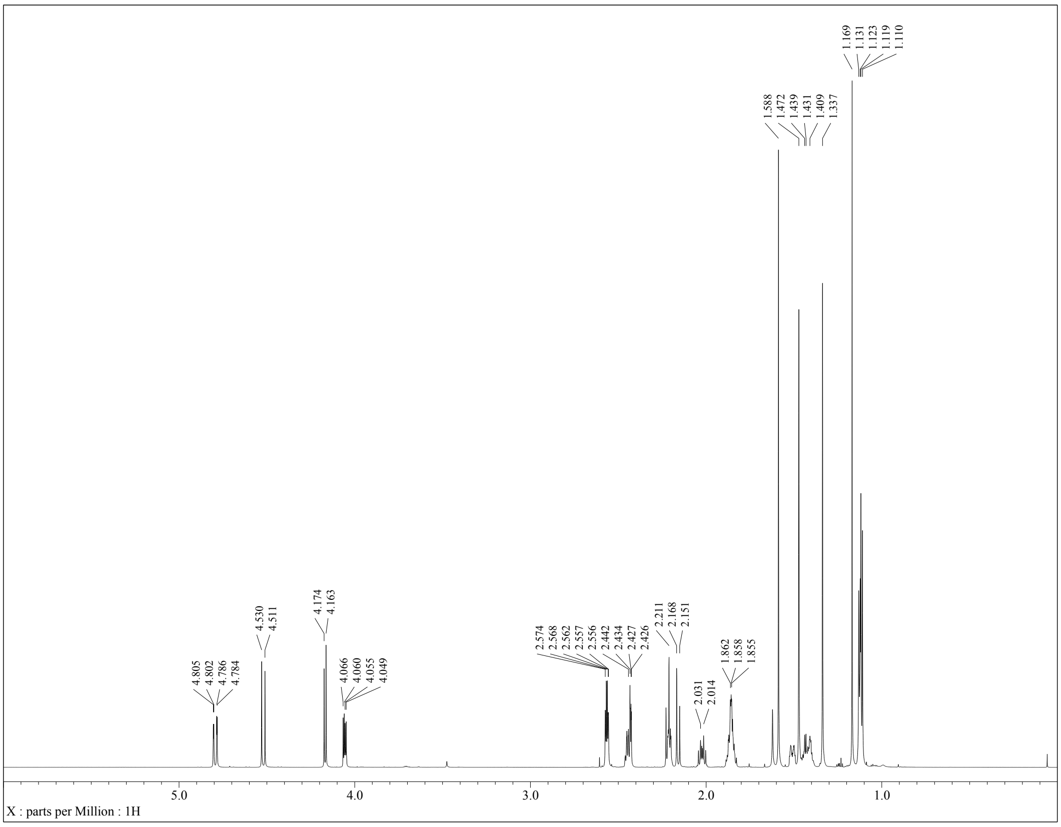


Supplementary Figure 71. $^{1}H$ NMR spectrum of **15**. See Supplementary Table 18 for the NMR data.

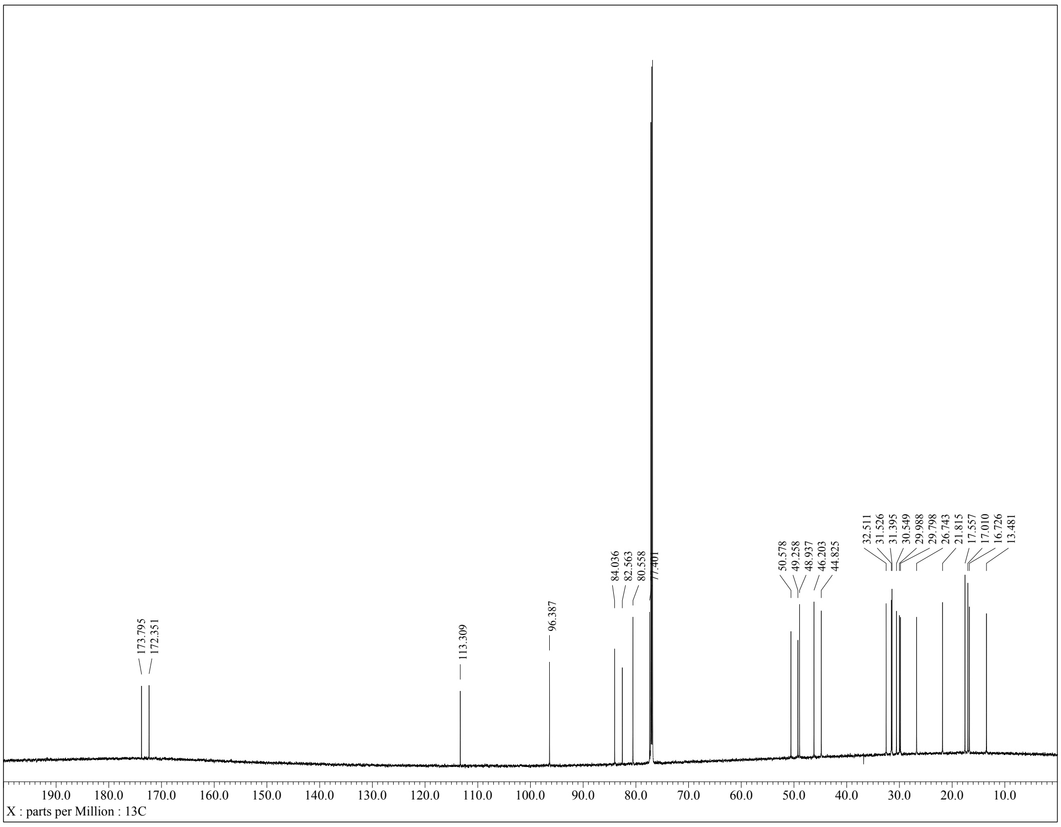


Supplementary Figure 72. $^{13}C$ NMR spectrum of **15**. See Supplementary Table 18 for the NMR data.

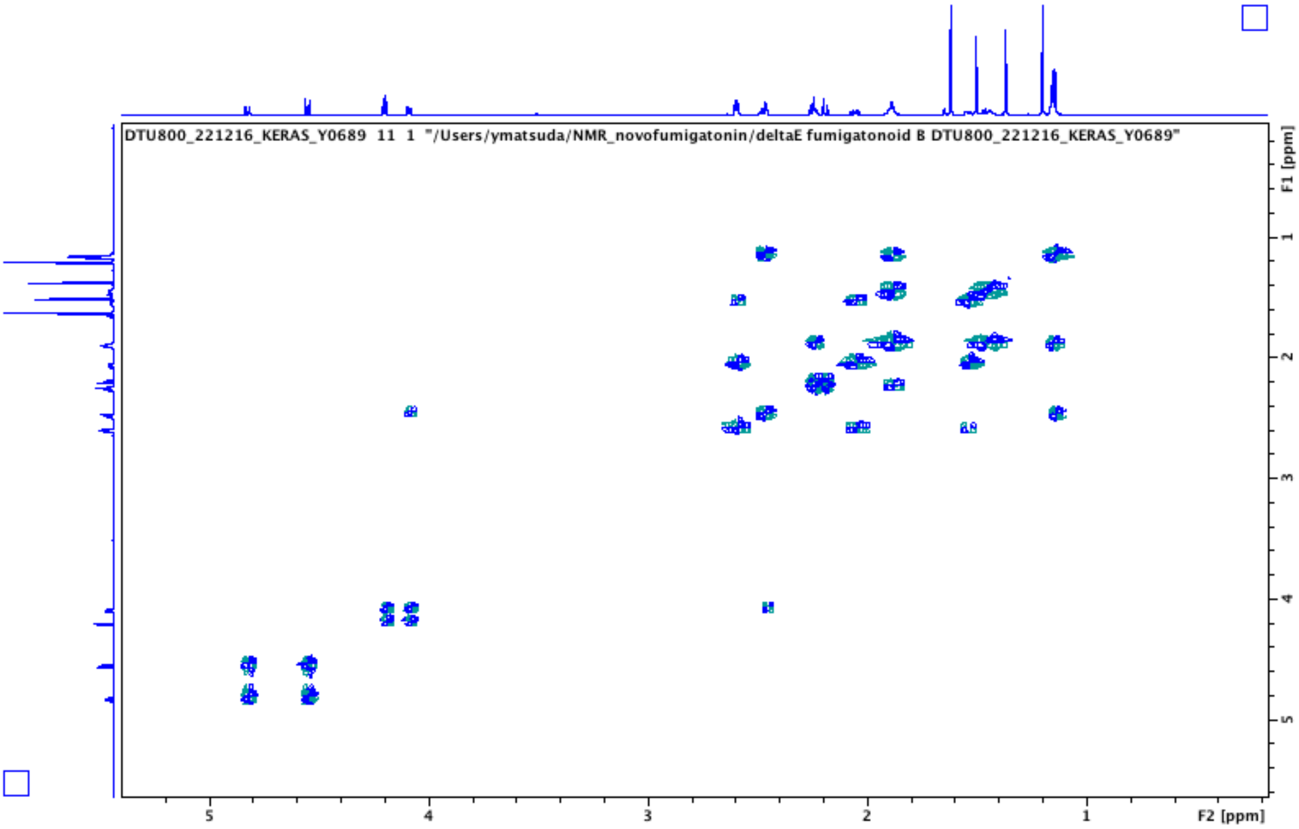


Supplementary Figure 73. $^{1}H$-$^{1}H$ DQF-COSY spectrum of **15**. See Supplementary Table 18 for the NMR data.

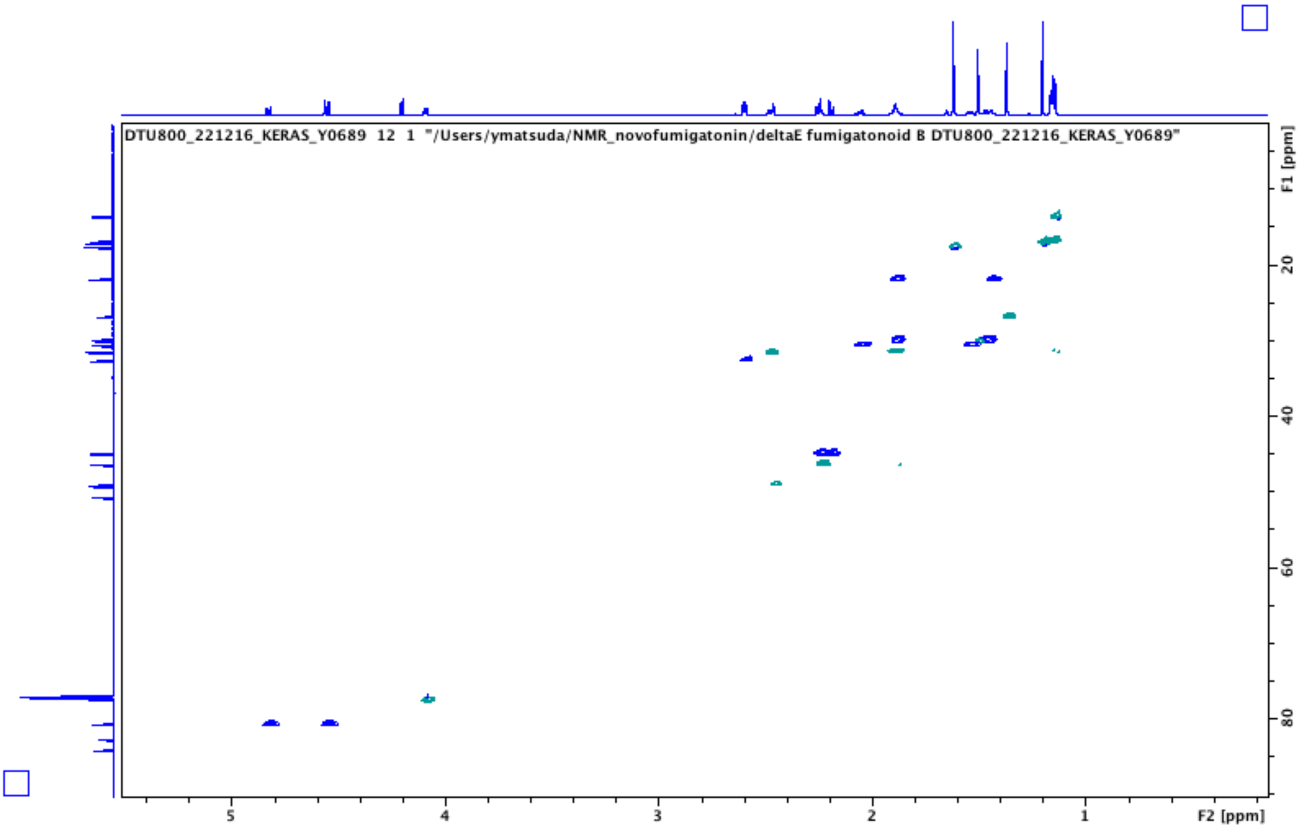


Supplementary Figure 74. HSQC spectrum of **15**. See Supplementary Table 18 for the NMR data.

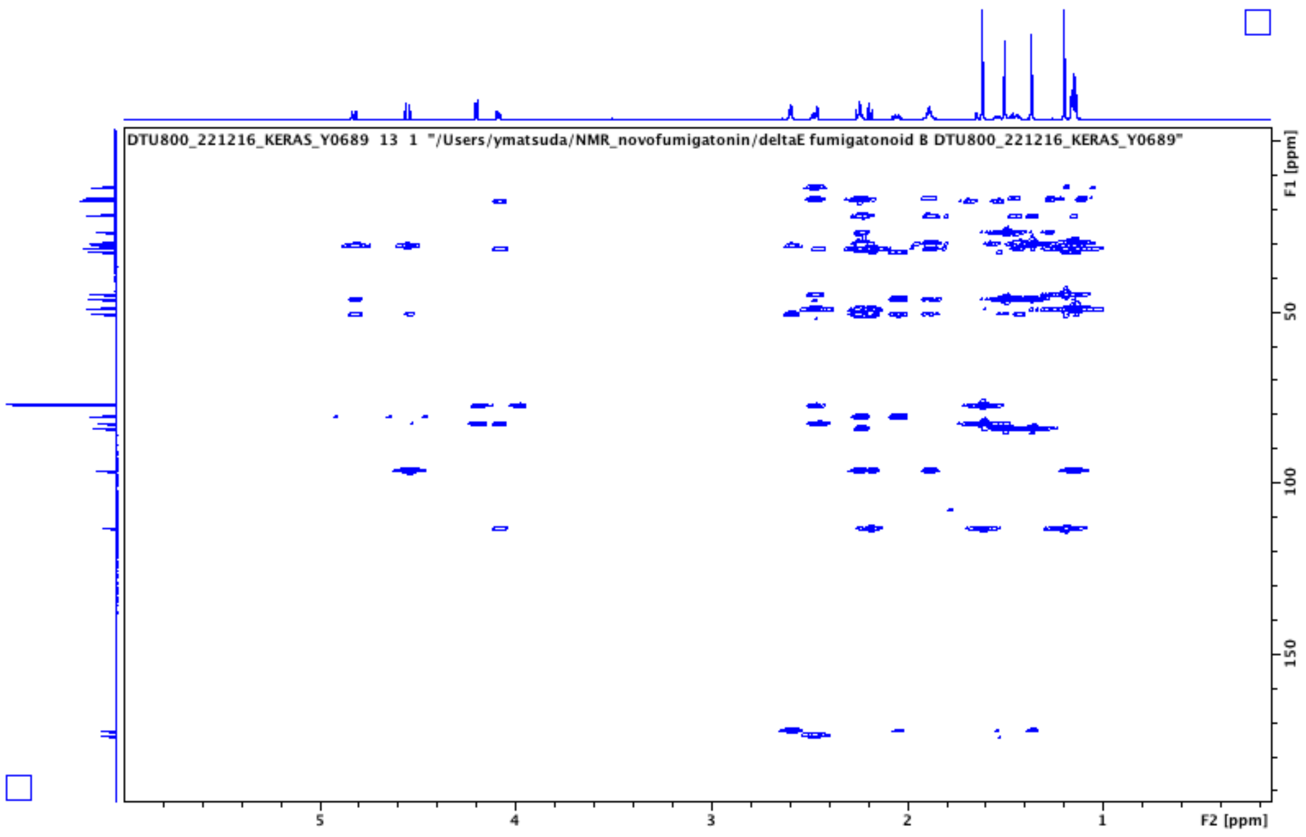


Supplementary Figure 75. HMBC spectrum of **15**. See Supplementary Table 18 for the NMR data.

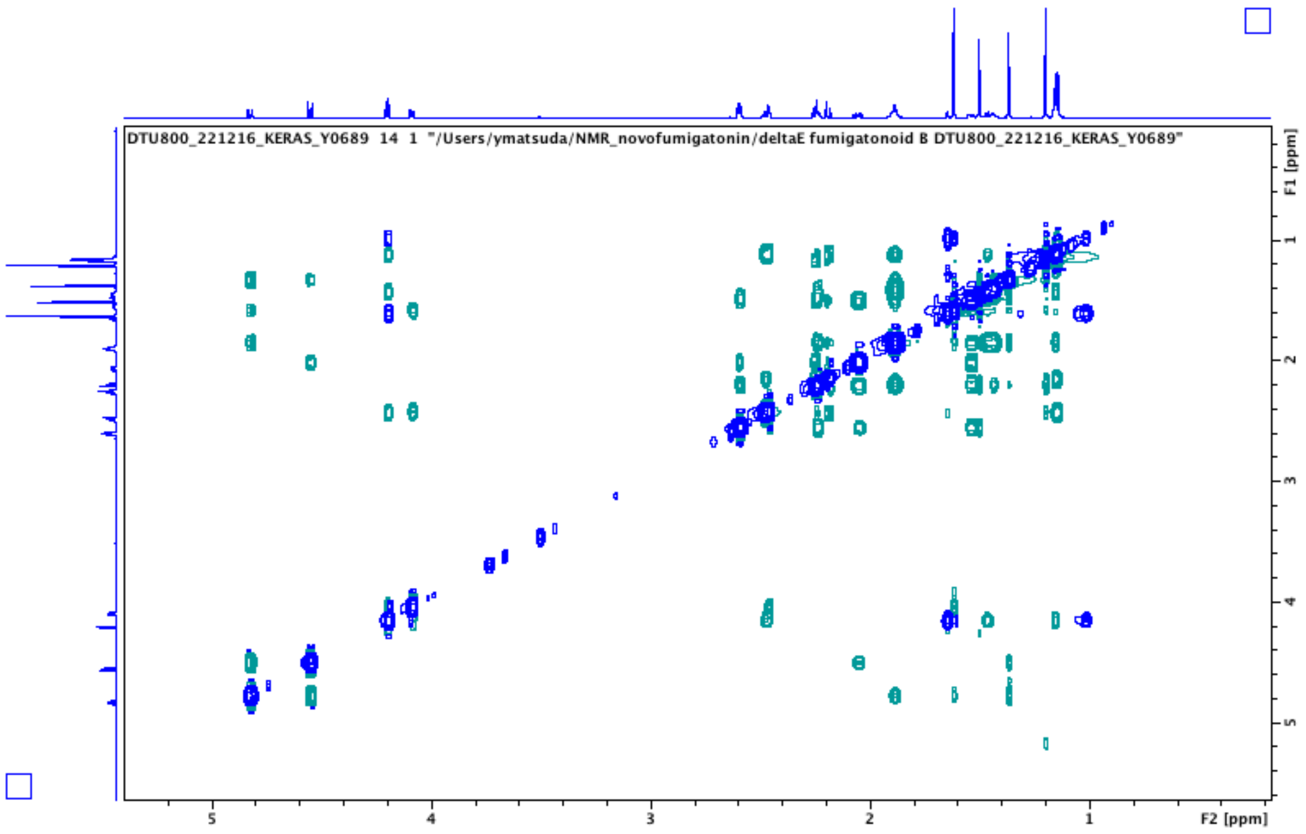


Supplementary Figure 76. NOESY spectrum of **15**. See Supplementary Table 18 for the NMR data.

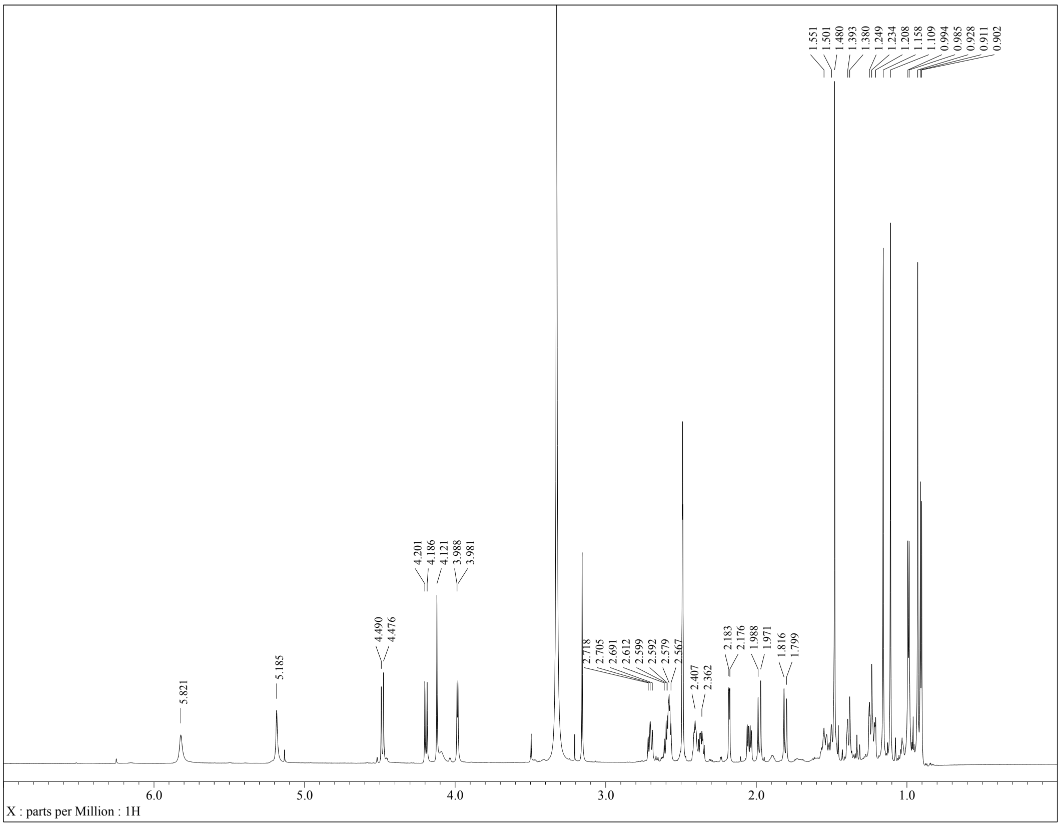


Supplementary Figure 77. $^{1}H$ NMR spectrum of **16**. See Supplementary Table 19 for the NMR data.

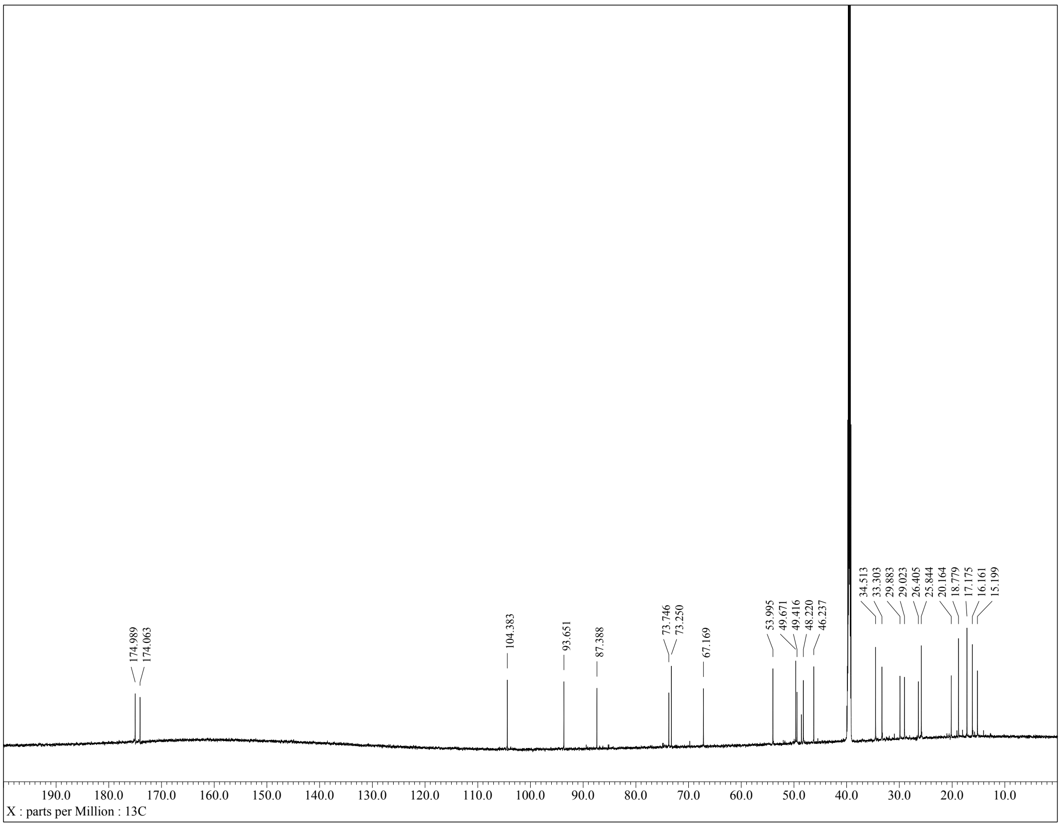


Supplementary Figure 78. $^{13}C$ NMR spectrum of **16**. See Supplementary Table 19 for the NMR data.

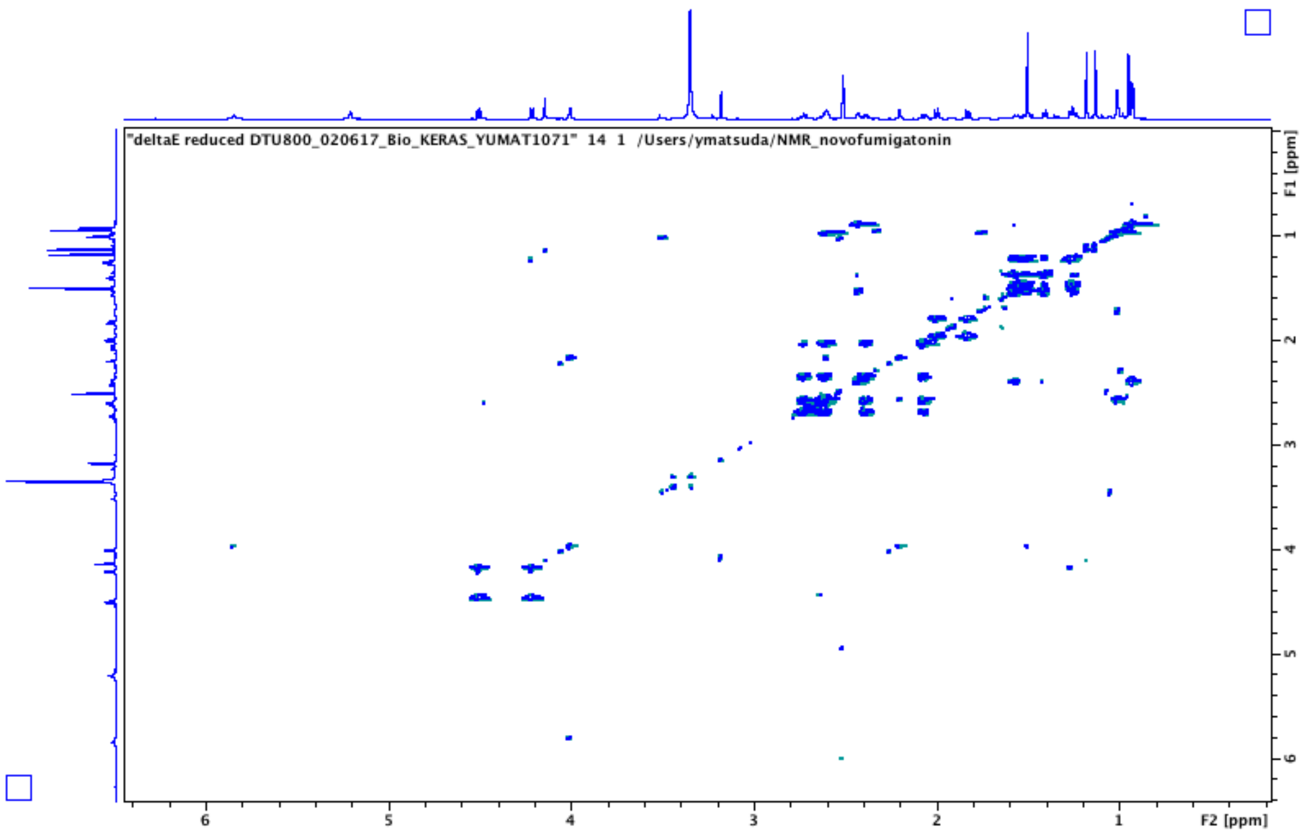


Supplementary Figure 79. $^{1}H$-$^{1}H$ DQF-COSY spectrum of **16**. See Supplementary Table 19 for the NMR data.

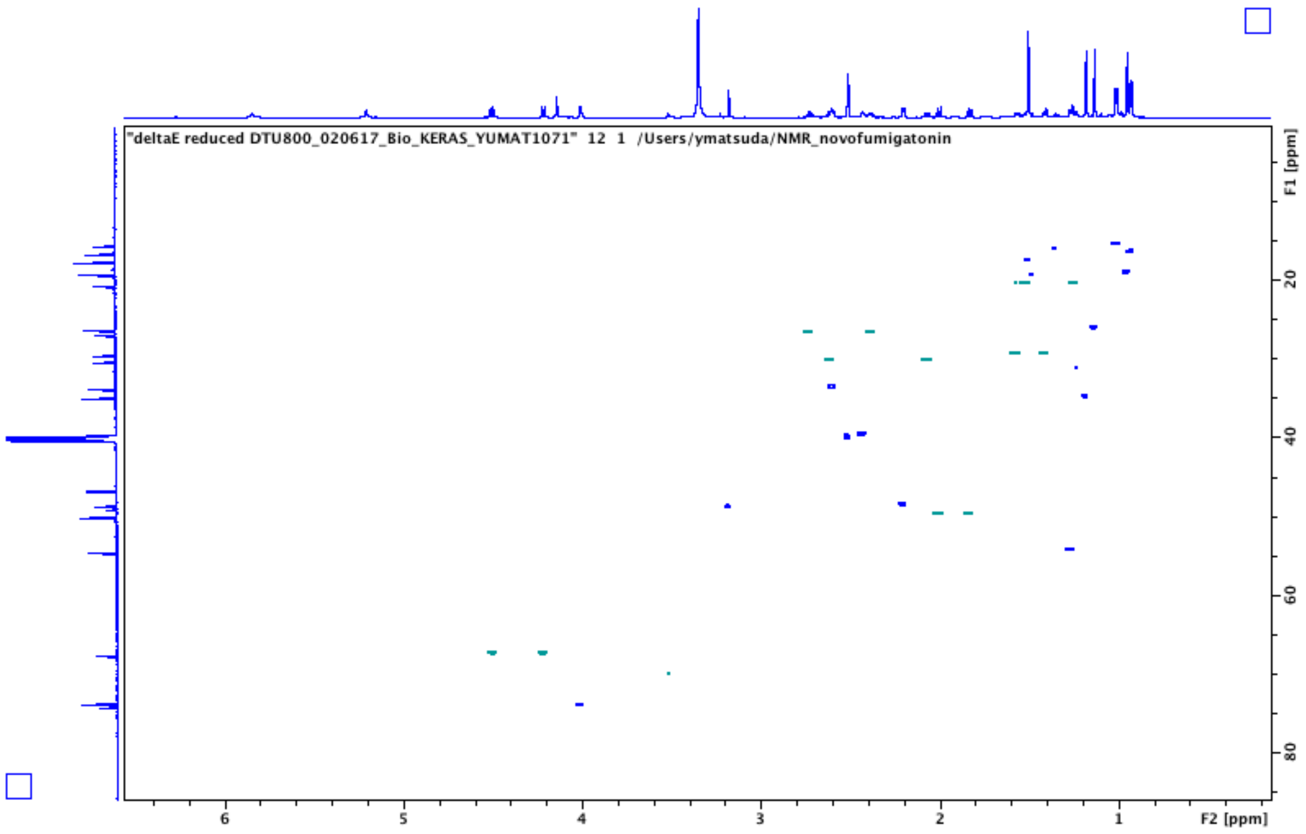


Supplementary Figure 80. HSQC spectrum of **16**. See Supplementary Table 19 for the NMR data.

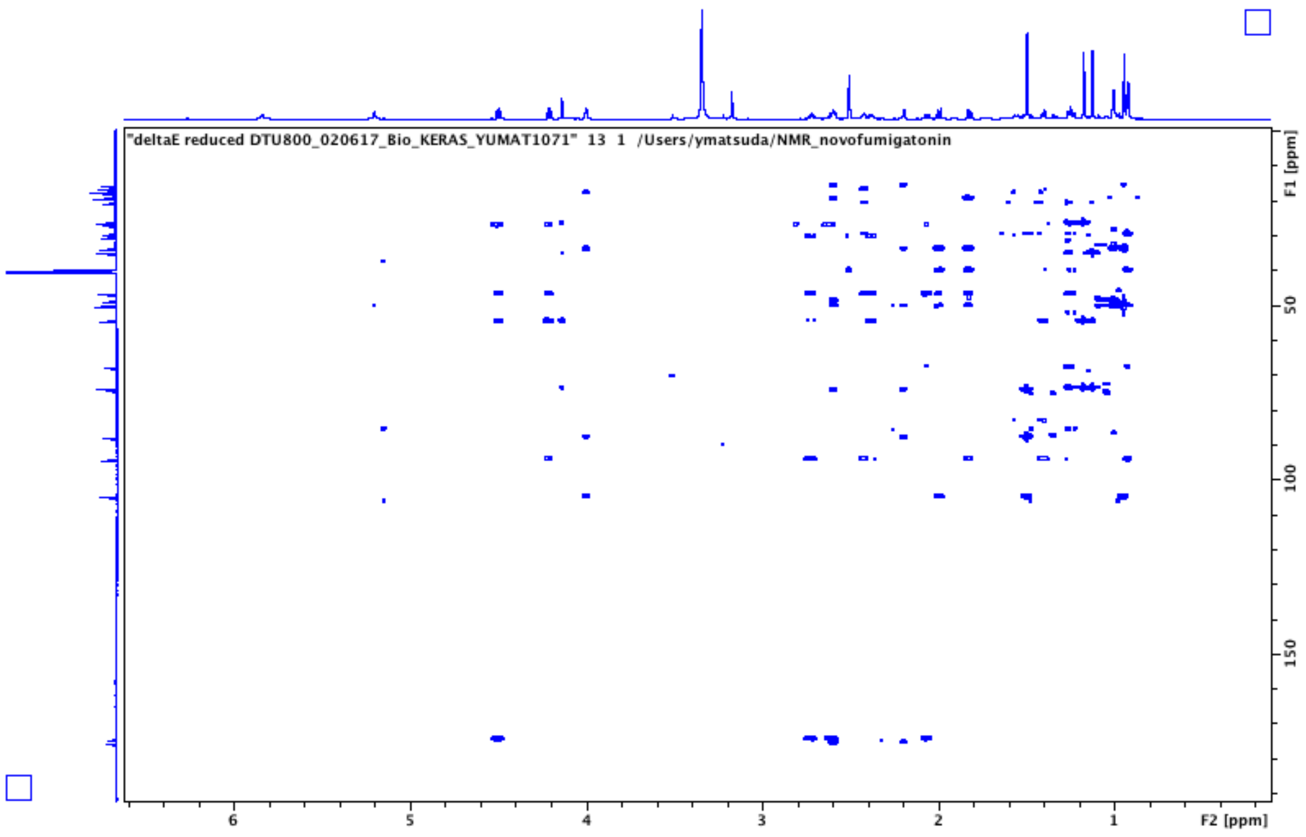


Supplementary Figure 81. HMBC spectrum of **16**. See Supplementary Table 19 for the NMR data.

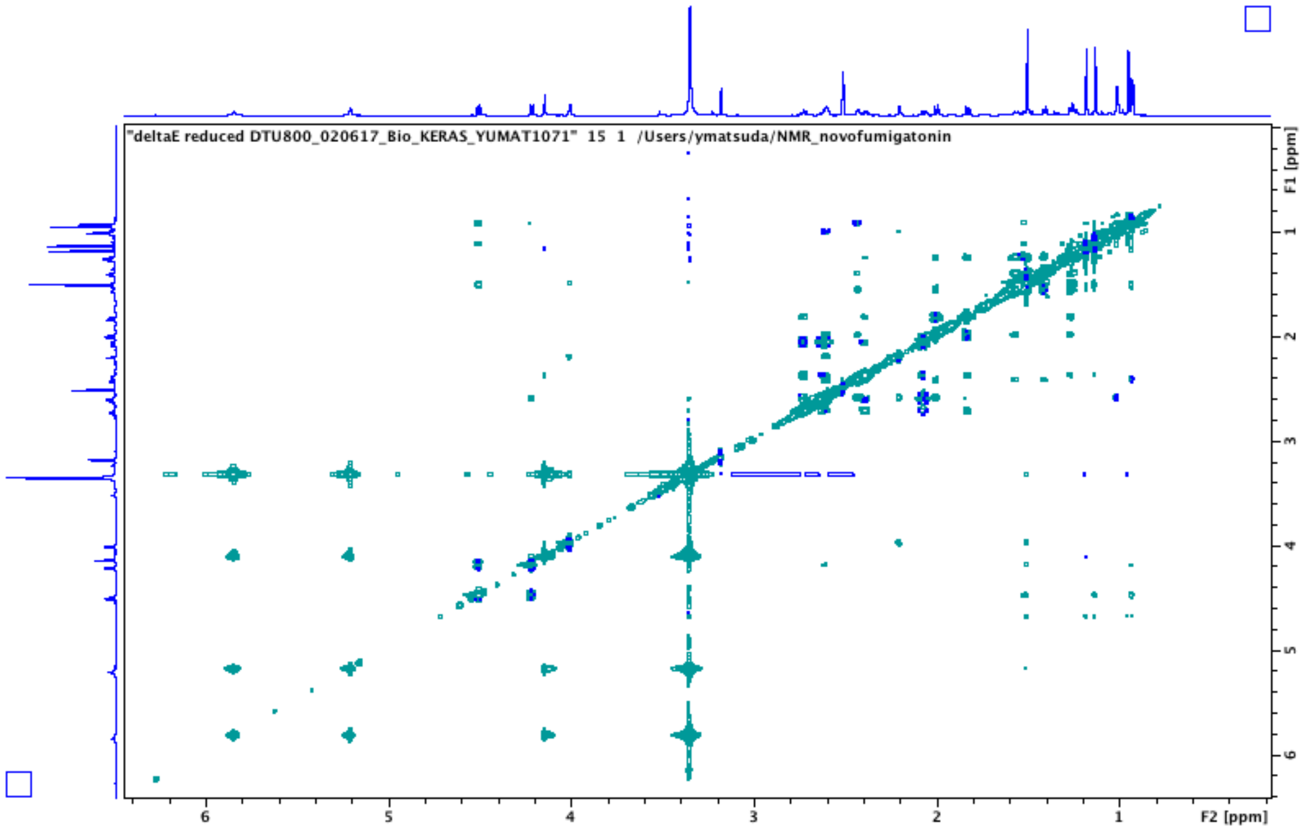


Supplementary Figure 82. NOESY spectrum of **16**. See Supplementary Table 19 for the NMR data.

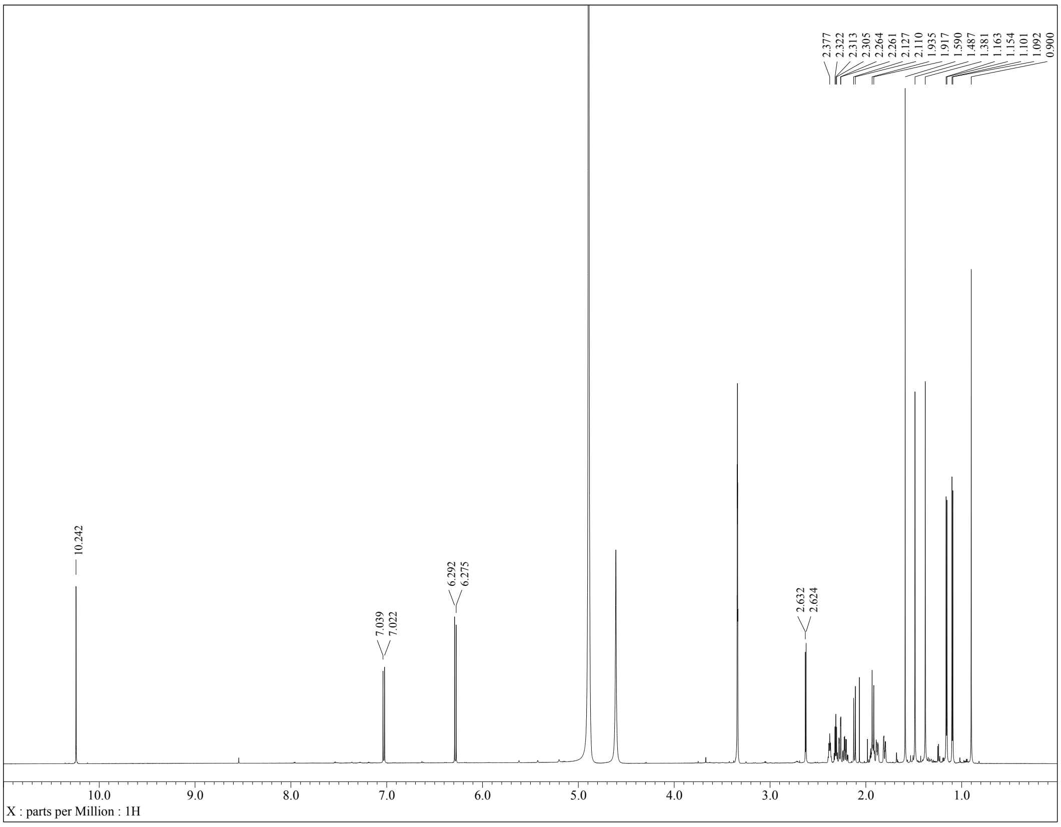


Supplementary Figure 83. $^{1}H$ NMR spectrum of **19**. See Supplementary Table 20 for the NMR data.

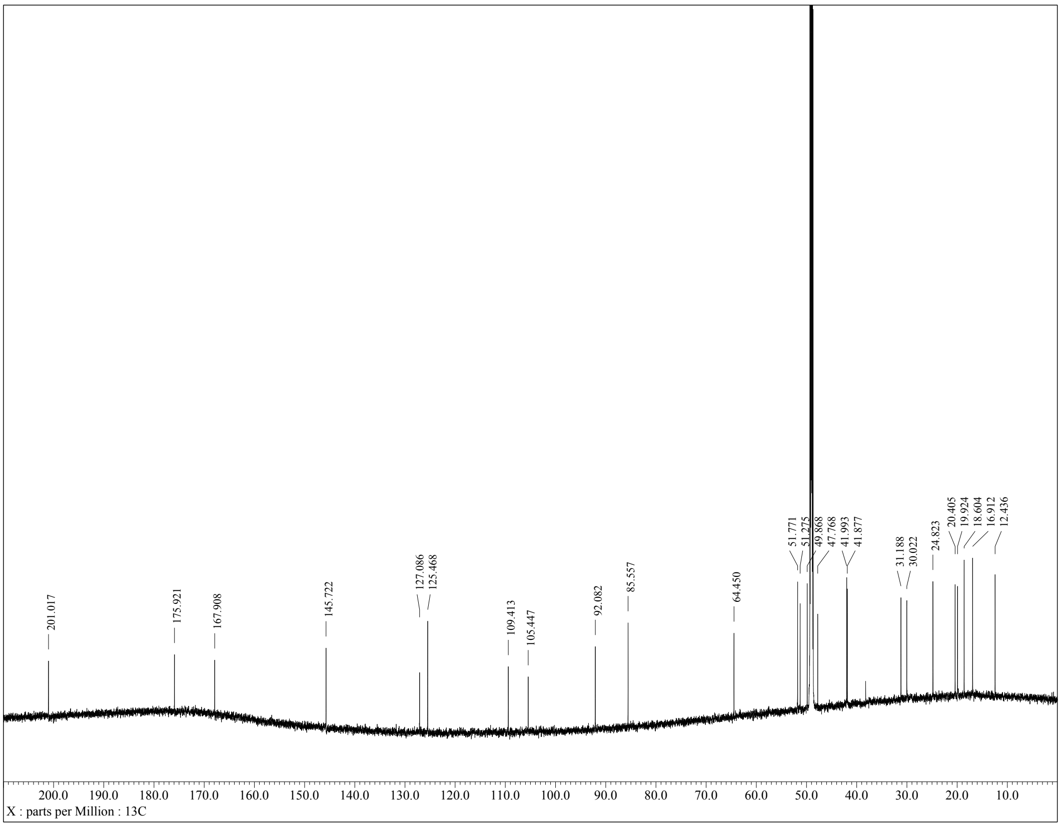


Supplementary Figure 84. $^{13}C$ NMR spectrum of **19**. See Supplementary Table 20 for the NMR data.

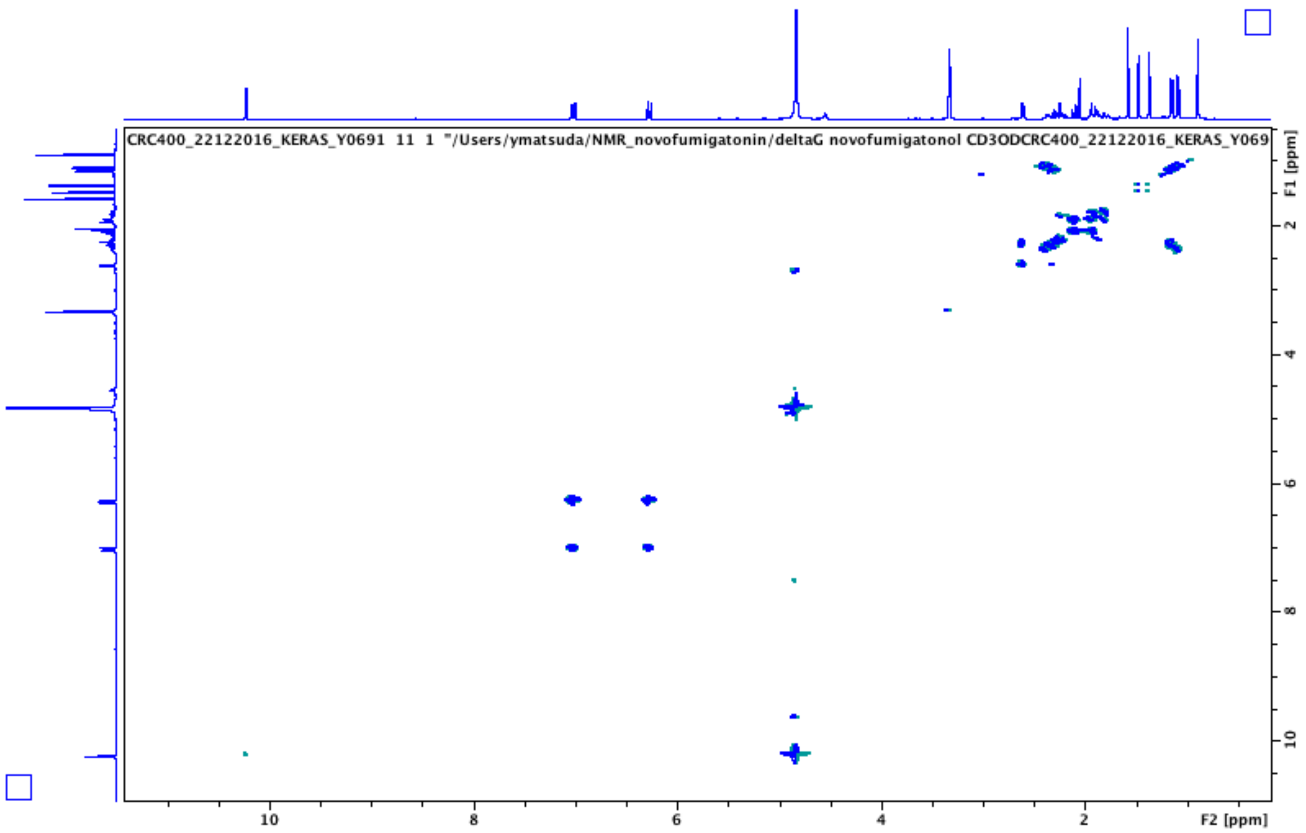


Supplementary Figure 85. $^{1}H$-$^{1}H$ DQF-COSY spectrum of **19**. See Supplementary Table 20 for the NMR data.

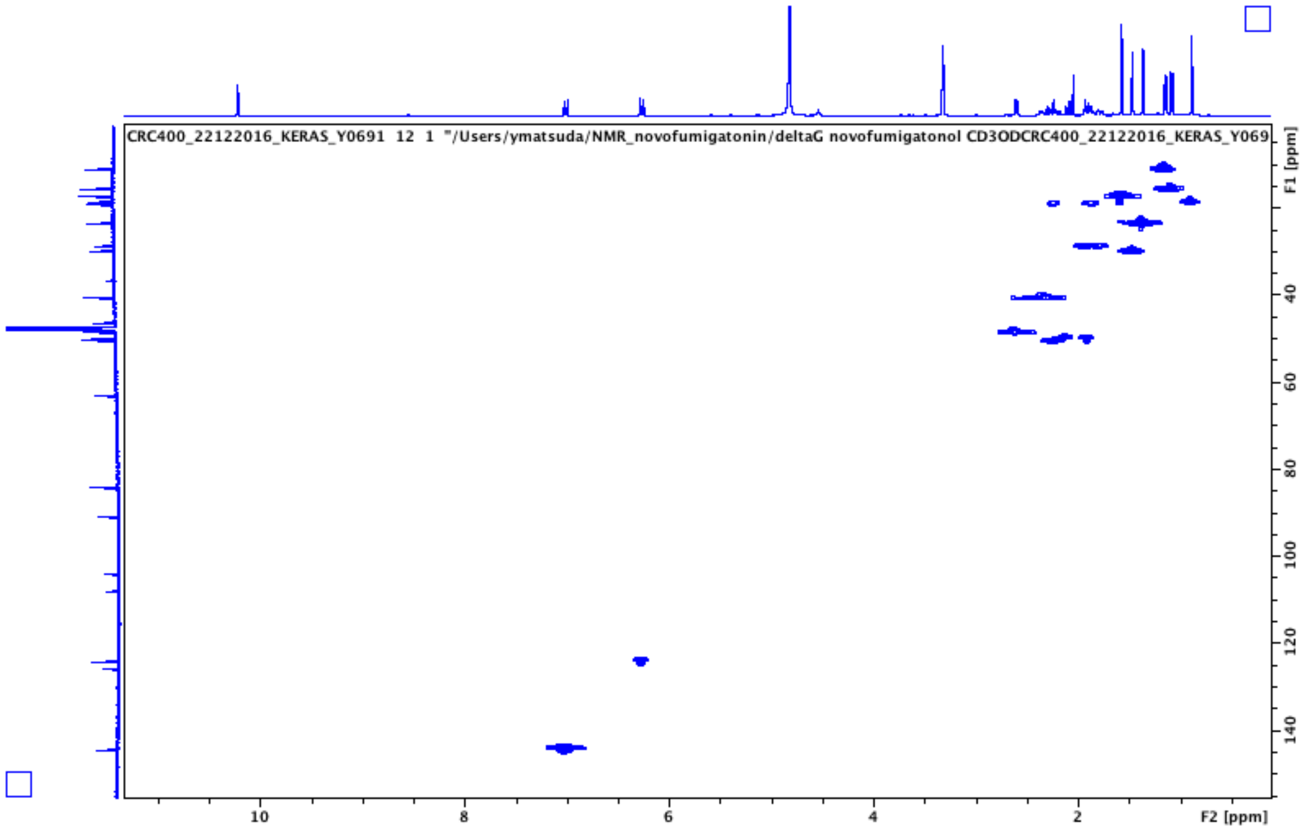


Supplementary Figure 86. HSQC spectrum of **19**. See Supplementary Table 20 for the NMR data.

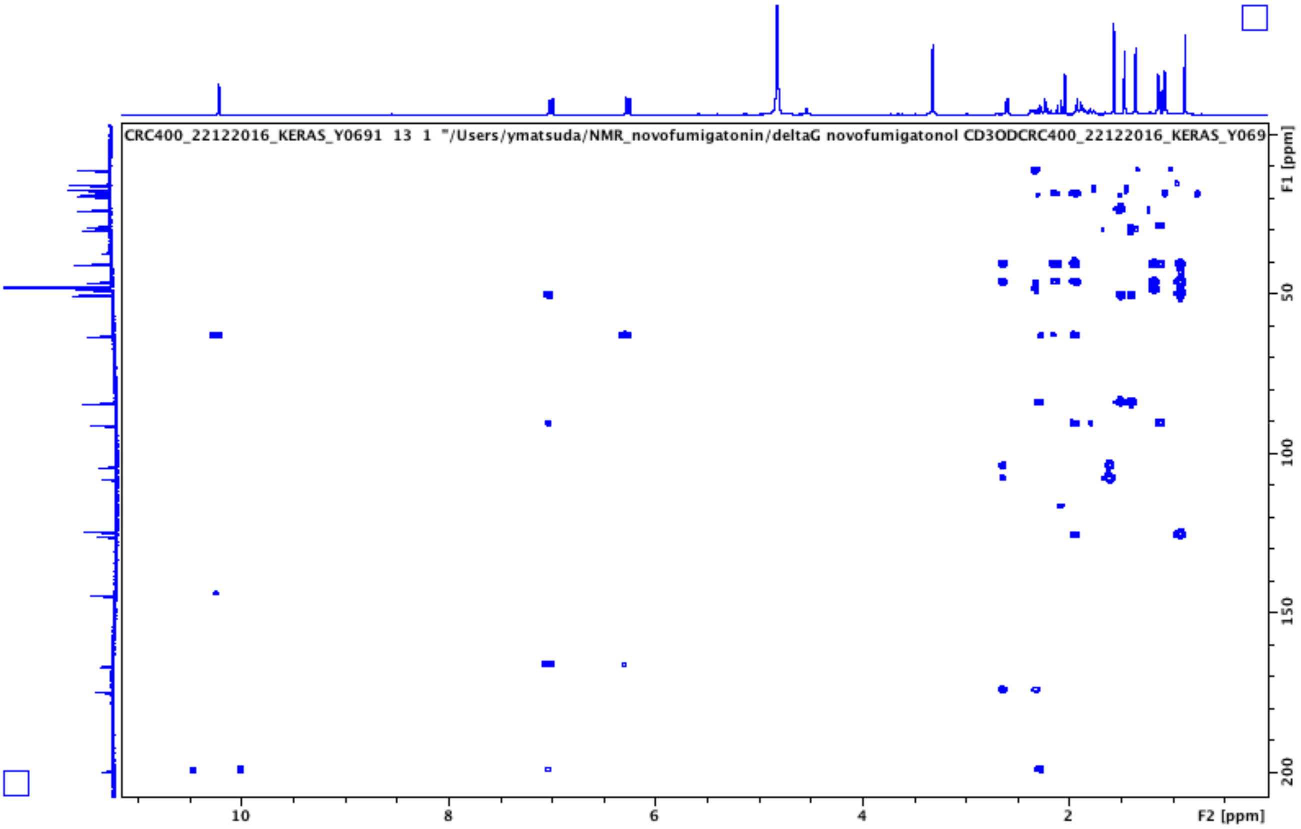


Supplementary Figure 87. HMBC spectrum of **19**. See Supplementary Table 20 for the NMR data.

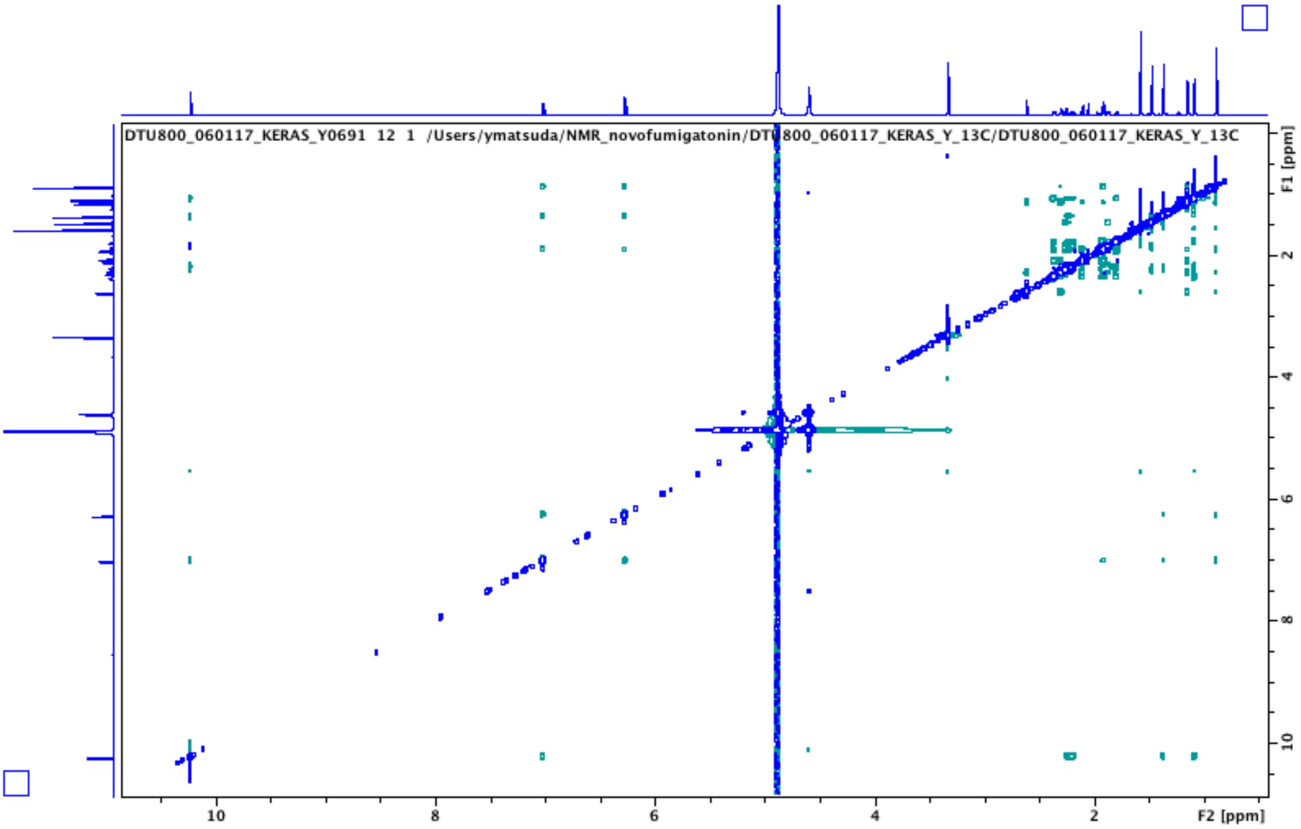


Supplementary Figure 88. NOESY spectrum of **19**. See Supplementary Table 20 for the NMR data.

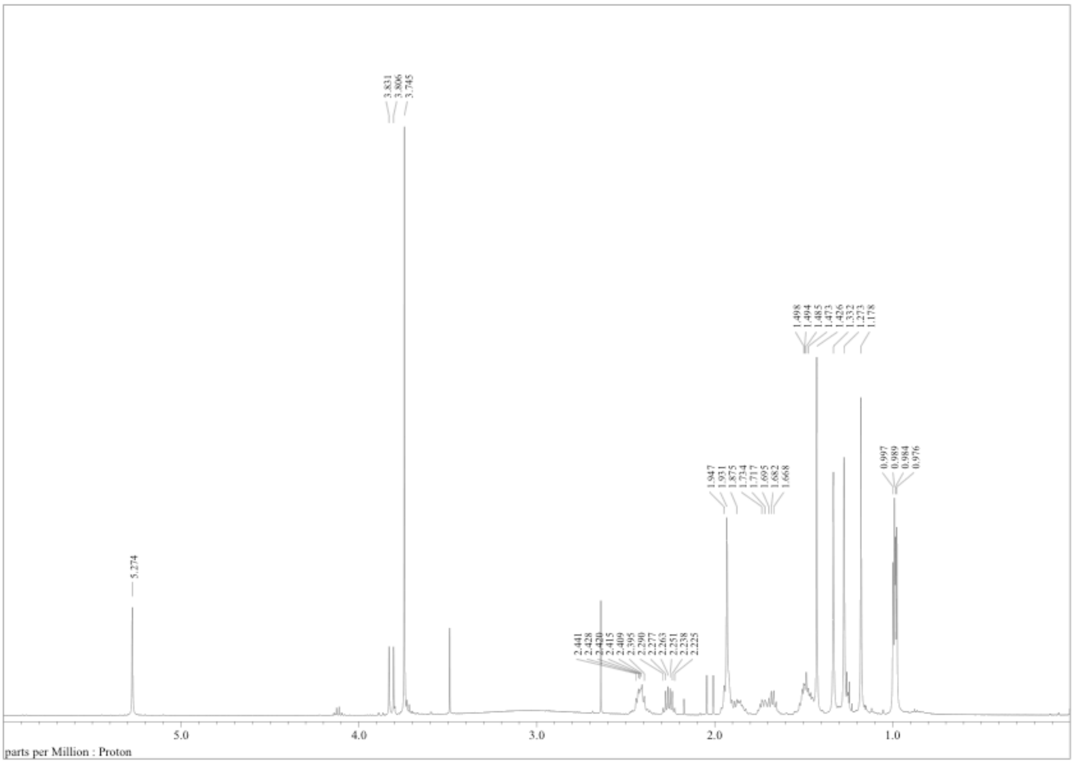


Supplementary Figure 89. $^{1}$H NMR spectrum of **21**. See Supplementary Table 21 for the NMR data.

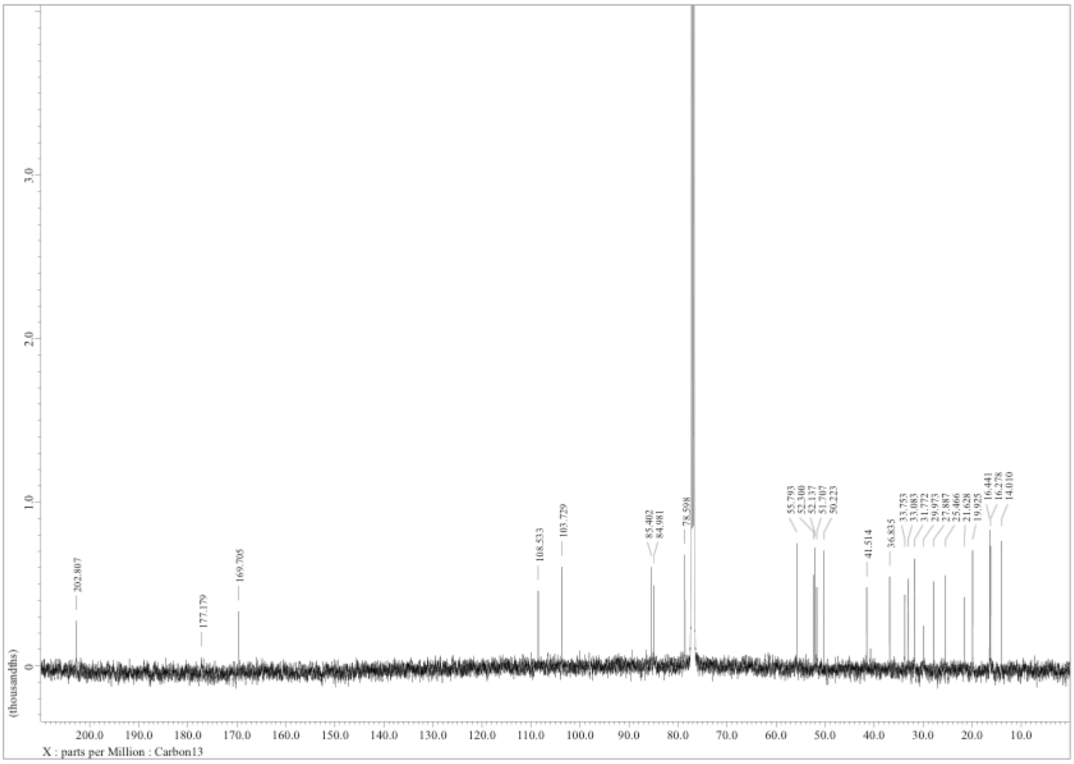


Supplementary Figure 90. $^{13}$C NMR spectrum of **21**. See Supplementary Table 21 for the NMR data.

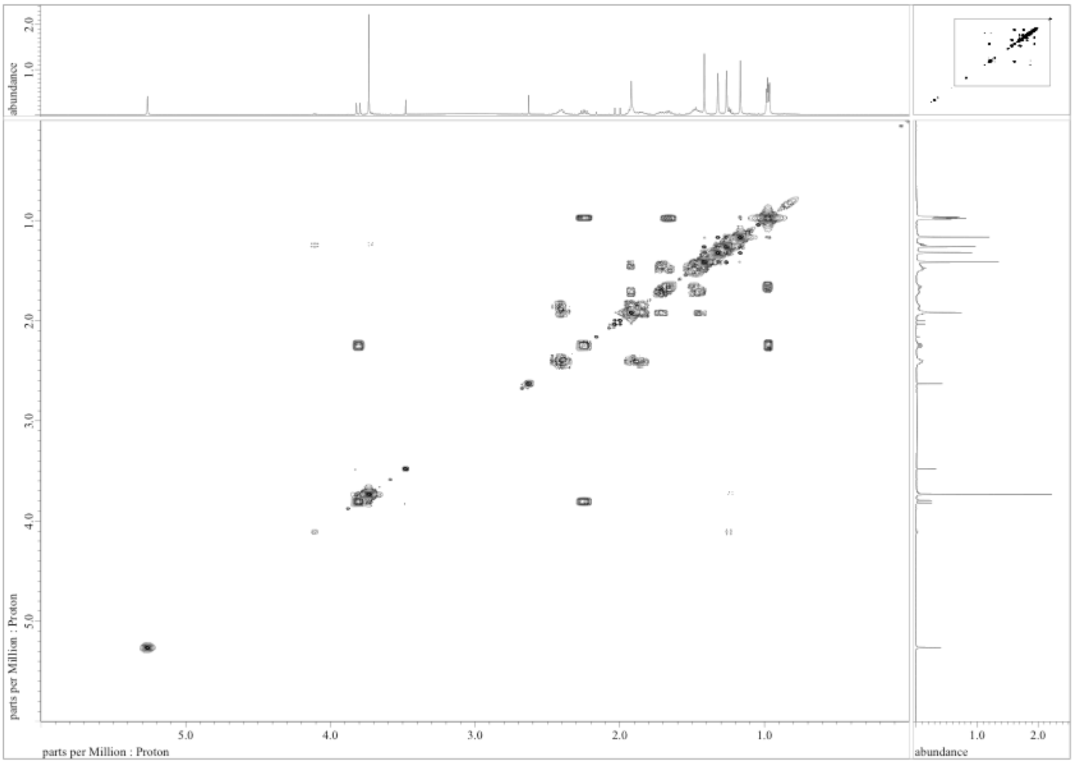


Supplementary Figure 91. $^{1}H$-$^{1}H$ COSY spectrum of **21**. See Supplementary Table 21 for the NMR data.

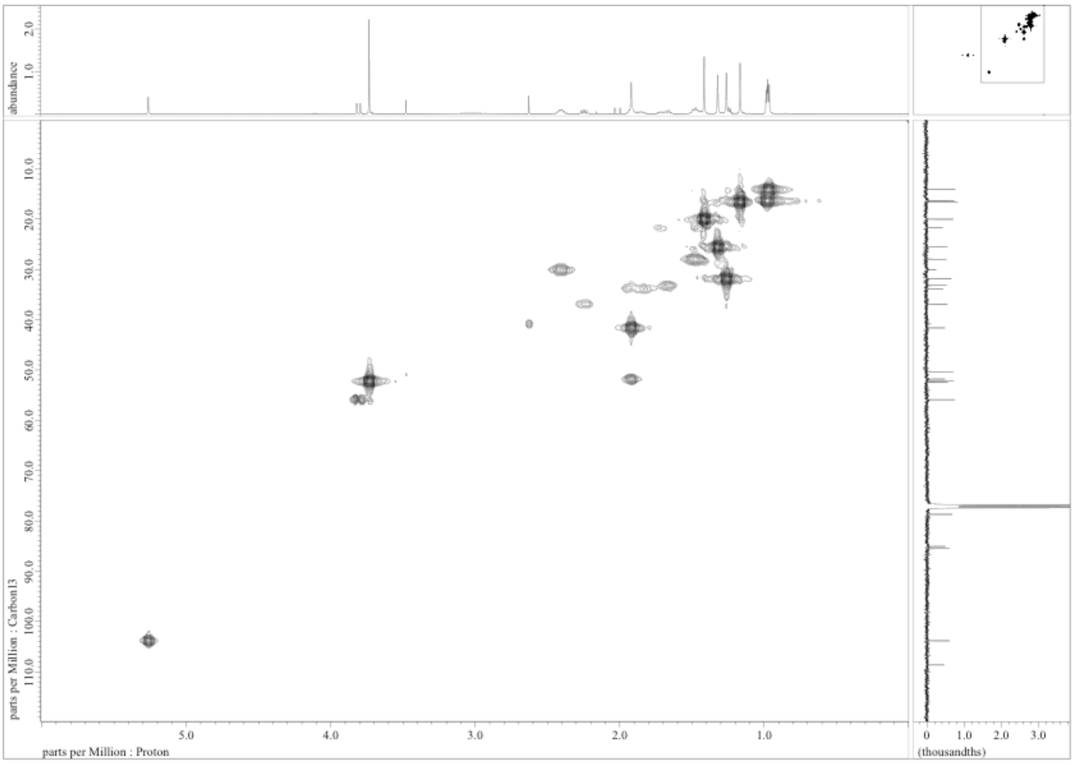


Supplementary Figure 92. HMQC spectrum of **21**. See Supplementary Table 21 for the NMR data.

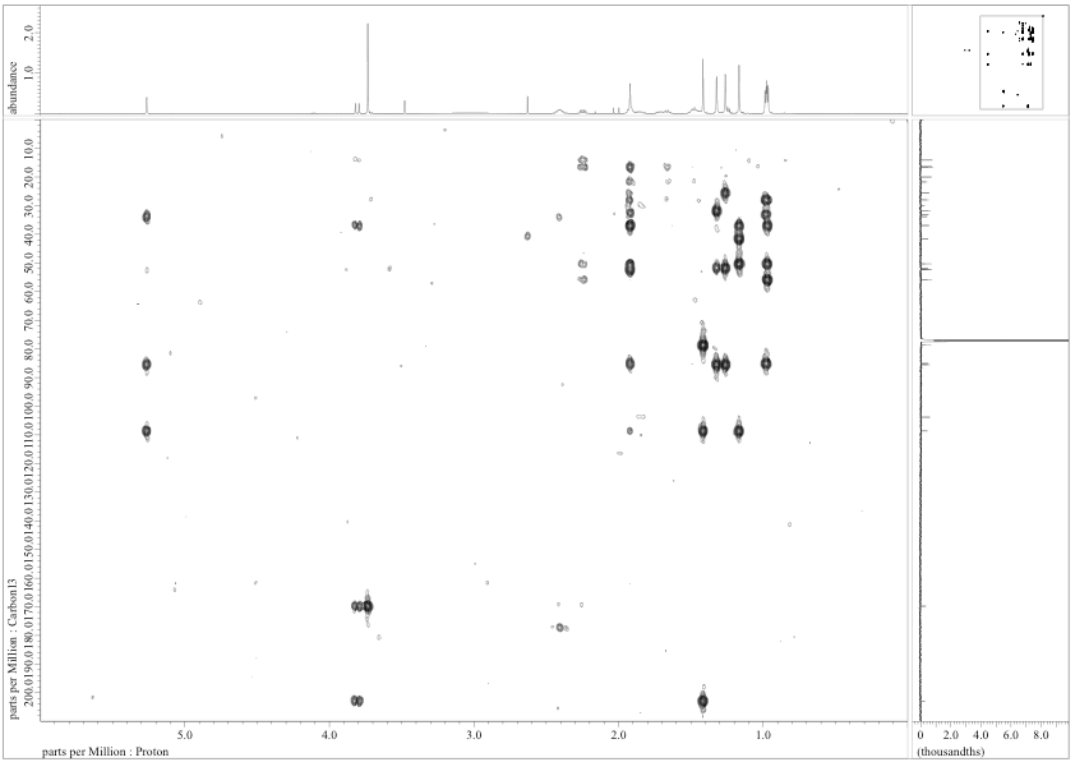


Supplementary Figure 93. HMBC spectrum of **21**. See Supplementary Table 21 for the NMR data.

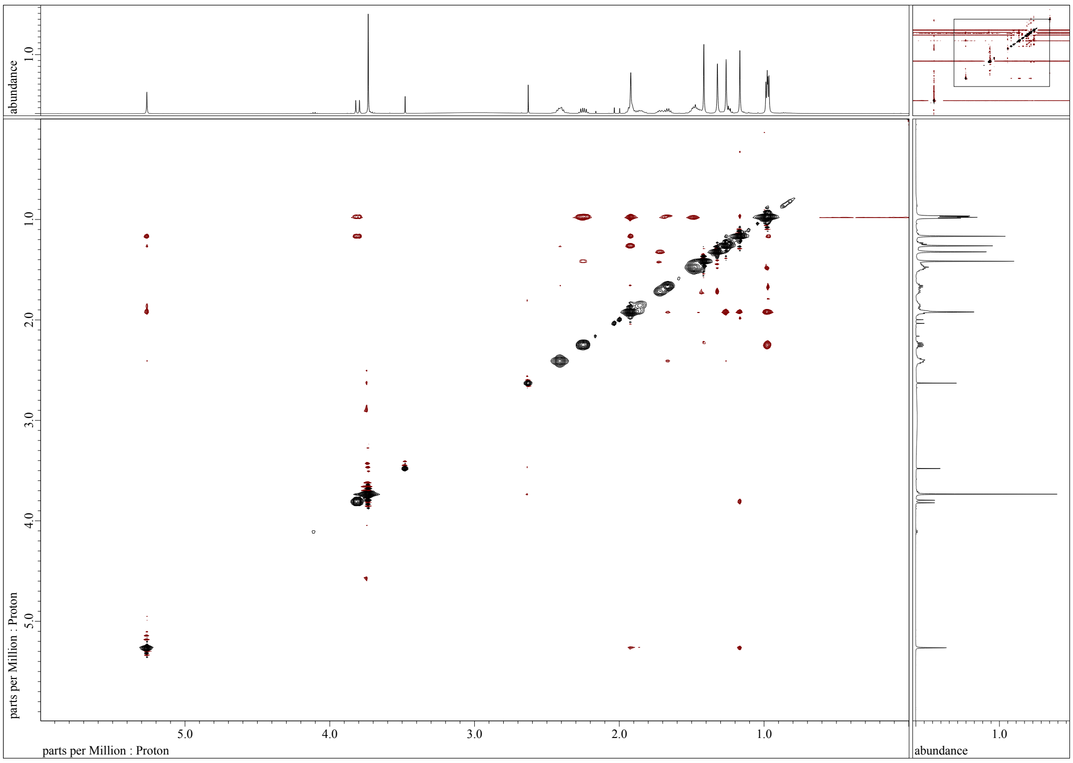


Supplementary Figure 94. NOESY spectrum of **21**. See Supplementary Table 21 for the NMR data.

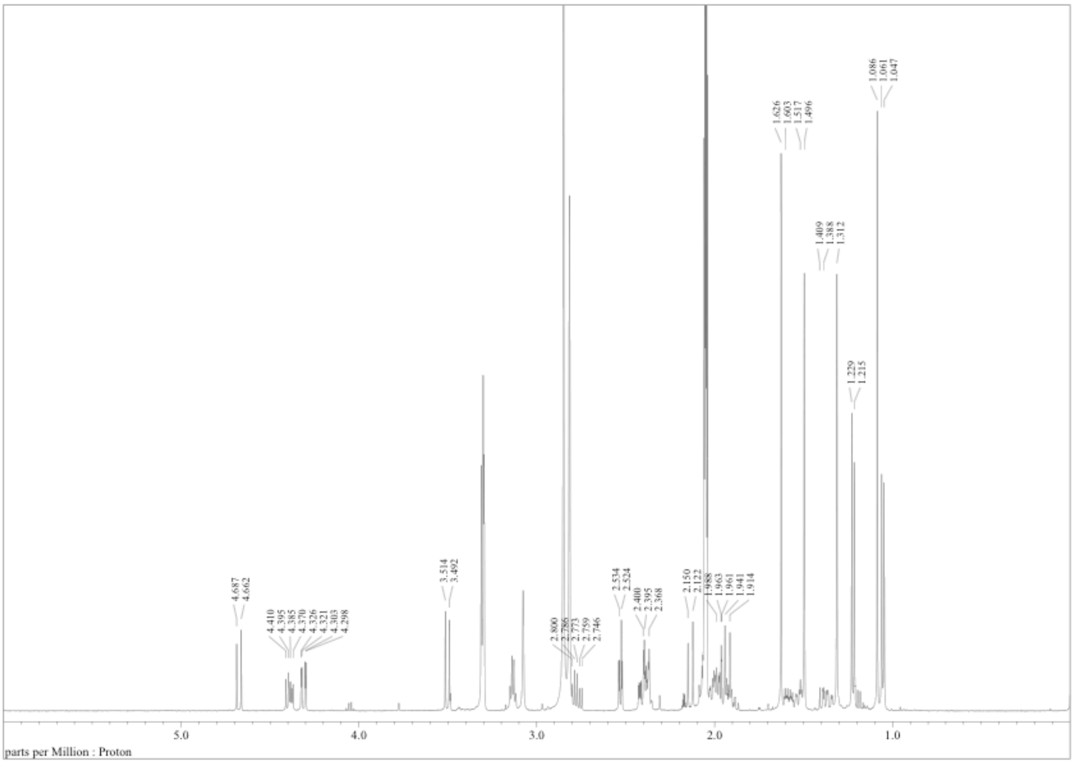

Supplementary Figure 95. $^{1}$H NMR spectrum of **26**. See Supplementary Table 22 for the NMR data.

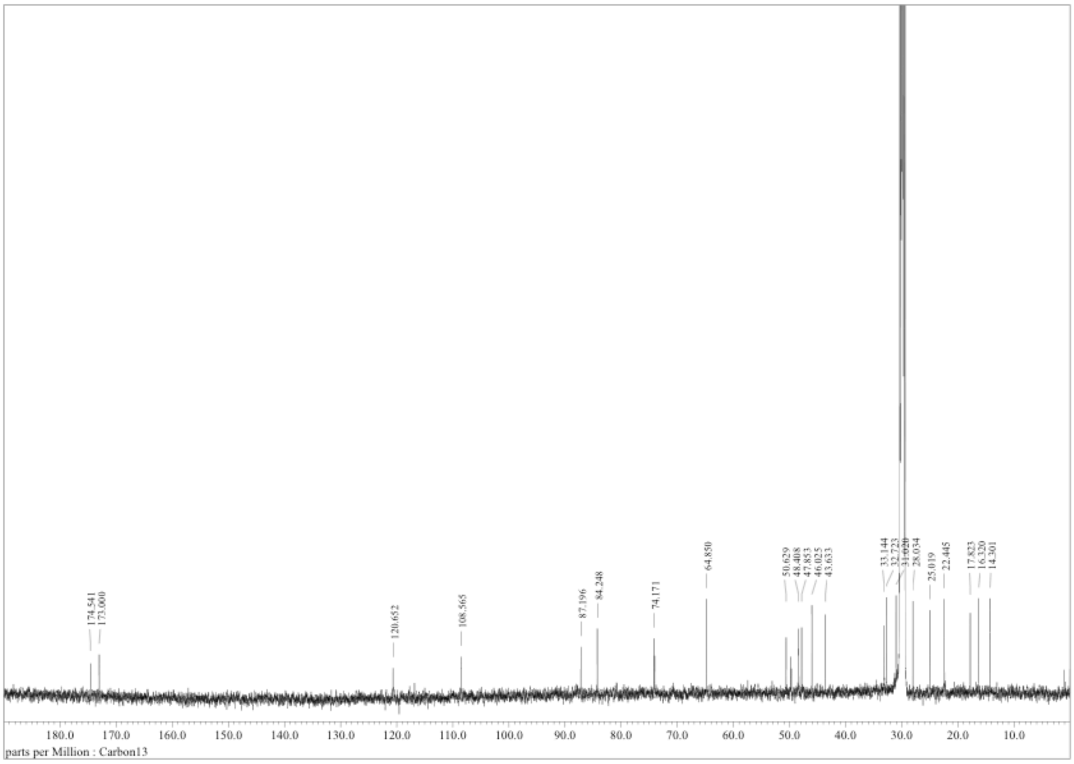

Supplementary Figure 96. $^{13}$C NMR spectrum of **26**. See Supplementary Table 22 for the NMR data.

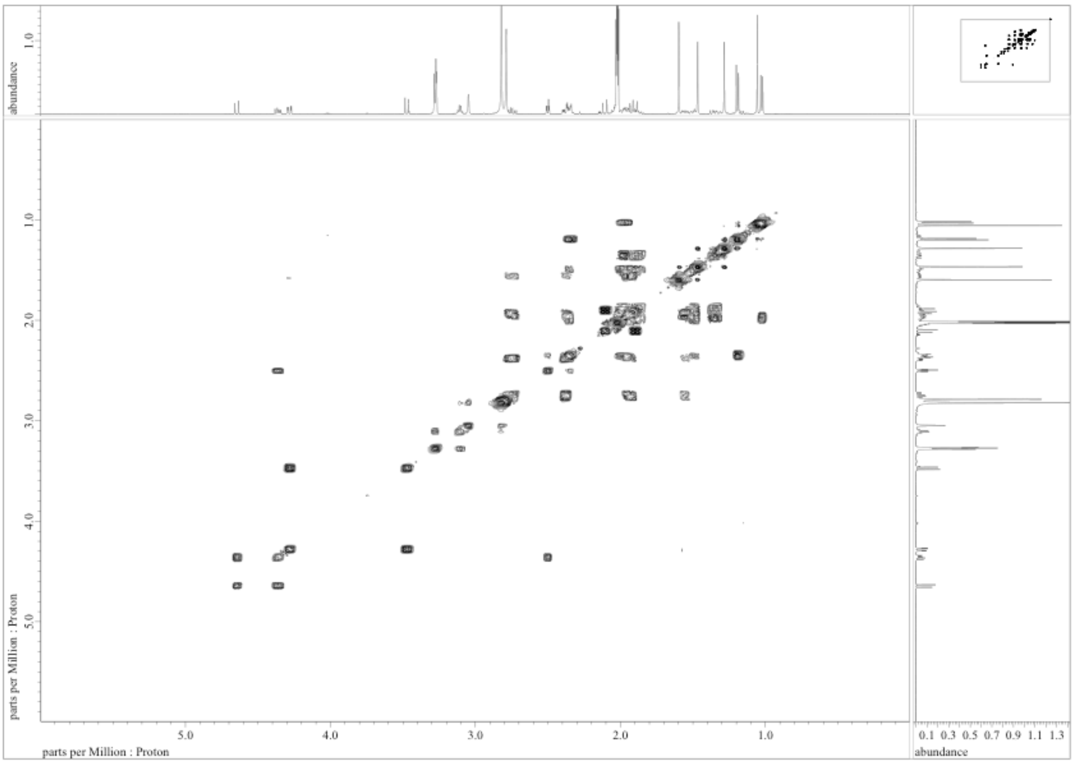


Supplementary Figure 97. ${}^{1}H$-${}^{1}H$ COSY spectrum of **26**. See Supplementary Table 22 for the NMR data.

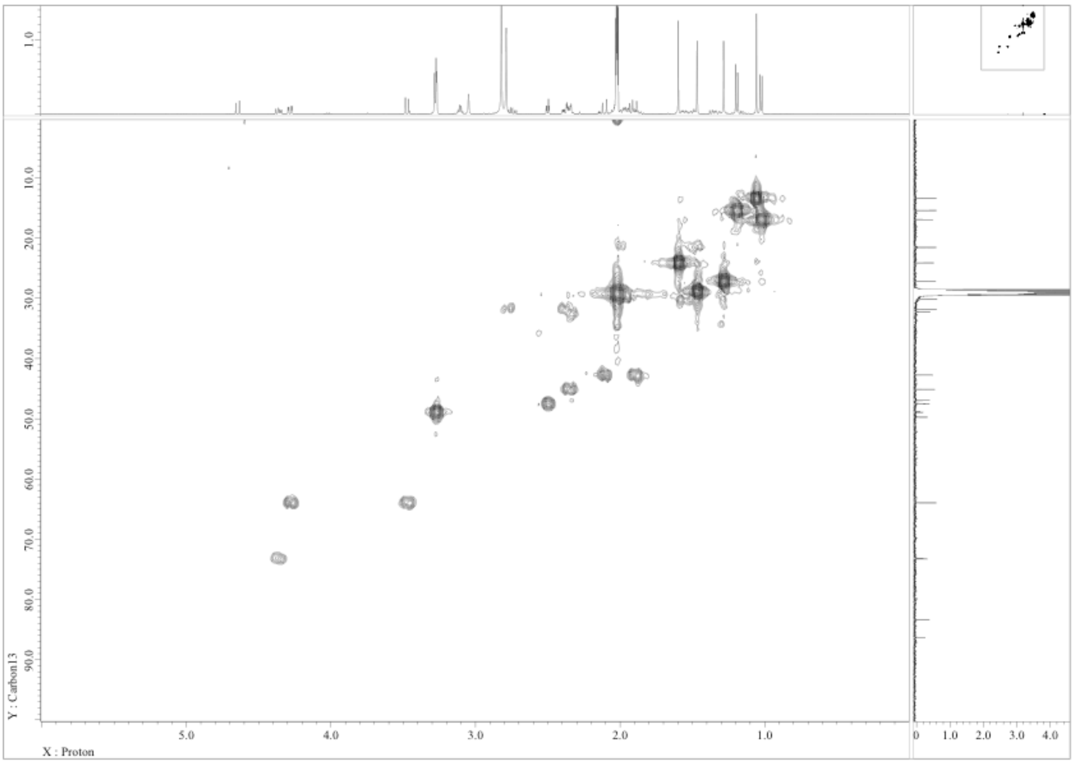


Supplementary Figure 98. HMQC spectrum of **26**. See Supplementary Table 22 for the NMR data.

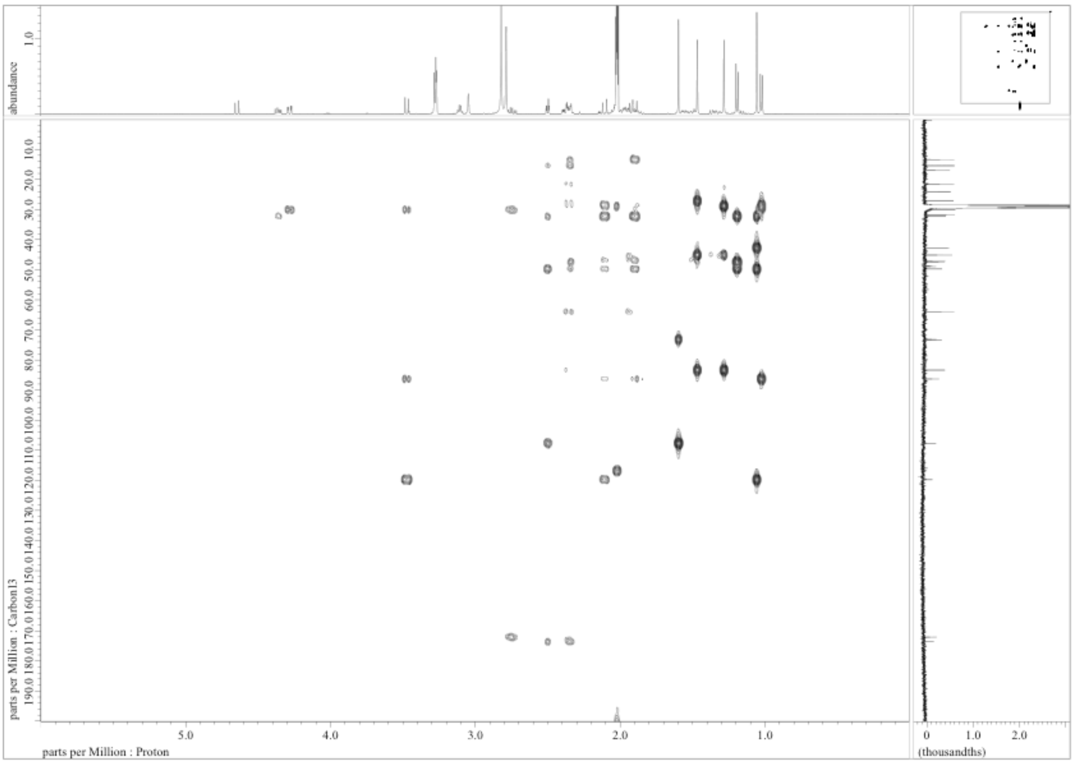


Supplementary Figure 99. HMBC spectrum of **26**. See Supplementary Table 22 for the NMR data.

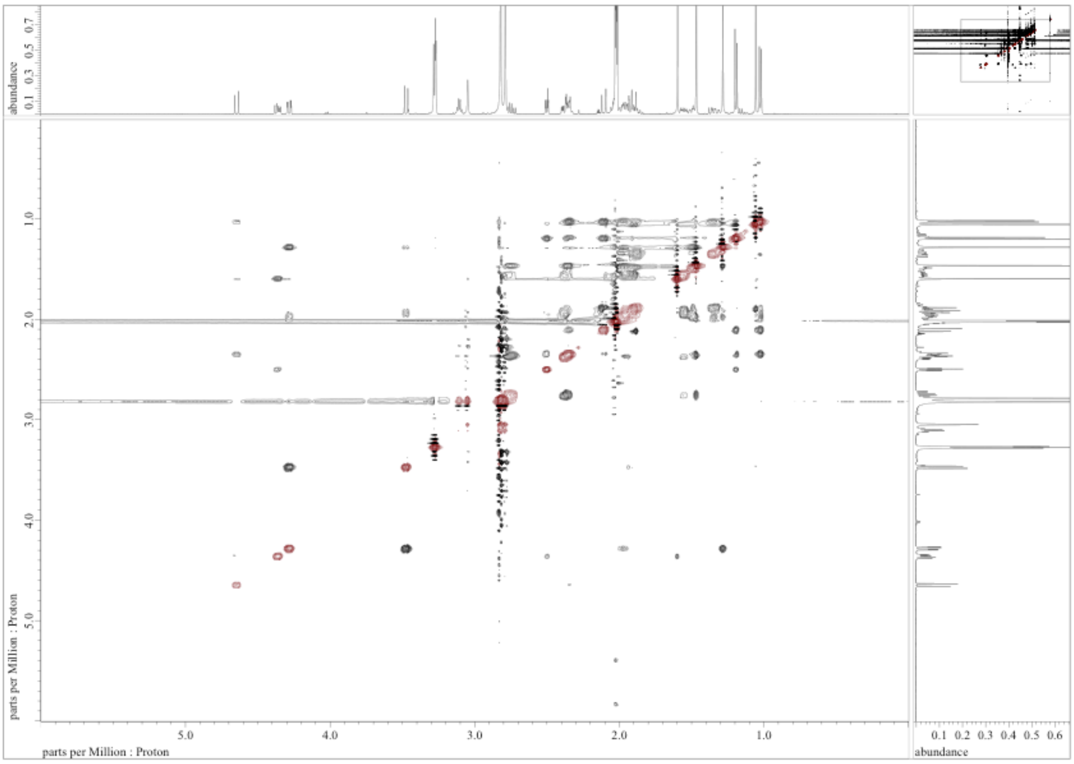


Supplementary Figure 100. NOESY spectrum of **26**. See Supplementary Table 22 for the NMR data.

## Supplementary Tables

Supplementary Table 1. Annotation of each protein of the *nvf* cluster.

| Gene | Protein ID | Amino acids (base pairs) | Protein homologue, origin | Similarity/identity (%) | Predicted function |
|---|---|---|---|---|---|
| *nvfA* | 455569 | 2255 (7131) | AndM, *Emericella variecolor* | 78/65 | polyketide synthase |
| | | | AdrD, *Penicillium chrysogenum* | 71/56 | |
| | | | AusA, *Aspergillus nidulans* | 70/56 | |
| *nvfB* | 425997 | 316 (951) | AdrG, *Penicillium chrysogenum* | 78/64 | prenyltransferase |
| | | | Trt2, *Aspergillus terreus* | 75/60 | |
| | | | PrhE, *Penicillium brasilianum* | 72/59 | |
| *nvfC* | 465152 | 257 (945) | Trt9, *Aspergillus terreus* | 84/70 | short chain dehydrogenase/reductase |
| | | | AdrF, *Penicillium chrysogenum* | 82/70 | |
| | | | PrhI, *Penicillium brasilianum* | 80/63 | |
| *nvfD* | 465151 | 250 (861) | PENSUB_6508, *Penicillium subrubescens* | 66/47 | α/β hydrolase |
| | | | PytH, *Sphingobium wenxiniae* | 38/25 | |
| *nvfE** | 334013 | 327 (1103) | PDE_00806, *Penicillium oxalicum* | 70/56 | Fe(II)/α-KG dependent dioxygenase |
| | | | Trt7, *Aspergillus terreus* | 47/27 | |
| | | | AusE, *Aspergillus nidulans* | 37/22 | |
| *nvfF* | 425993 | 298 (897) | EasH, *Claviceps purpurea* | 59/44 | Fe(II)/α-KG dependent dioxygenase |
| | | | Trt7, *Aspergillus terreus* | 53/35 | |
| | | | AusE, *Aspergillus nidulans* | 52/35 | |
| *nvfG* | 465148 | 342 (1288) | AdrE, *Penicillium chrysogenum* | 64/44 | short chain dehydrogenase/reductase |
| | | | TerH, *Aspergillus terreus* | 53/35 | |
| *nvfH* | 425991 | 661 (1986) | Trt3, *Aspergillus terreus* | 73/58 | FAD-dependent monooxygenase |
| | | | AusC, *Aspergillus nidulans* | 70/56 | |
| | | | AndJ, *Emericella variecolor* | 66/50 | |
| *nvfI** | 436154 | 278 (1079) | AFLA_064490, *Aspergillus flavus* | 58/44 | Fe(II)/α-KG dependent dioxygenase |
| | | | CmcJ, *Streptomyces clavuligerus* | 43/31 | |
| | | | $GA_4$ desaturase, *Fusarium fujikuroi* | 33/24 | |
| *nvfJ* | 425989 | 278 (1029) | AdrK, *Penicillium chrysogenum* | 71/53 | methyltransferase |
| | | | AusD, *Aspergillus nidulans* | 70/51 | |
| | | | Trt5, *Aspergillus terreus* | 69/47 | |
| *nvfK* | 473479 | 470 (1599) | AndE, *Emericella variecolor* | 78/62 | FAD-dependent monooxygenase |
| | | | PenE, *Penicillium thymicola* | 75/57 | |
| | | | PrhF, *Penicillium brasilianum* | 70/51 | |
| *nvfL* | 380360 | 243 (791) | AndB, *Emericella variecolor* | 63/49 | terpene cyclase |
| | | | AtmB, *Aspergillus flavus* | 61/43 | |
| | | | PrhH, *Penicillium brasilianum* | 55/33 | |
| *nvfM* | 436150 | 327 (1193) | ABOM_003114, *Aspergillus bombycis* | 76/60 | short chain dehydrogenase/reductase |
| | | | isoflavone reductase, *Medicago sativa* | 41/25 | |

Note: Protein IDs are as designated in JGI database. The *nvfM* gene was not originally included in the cluster in our previous work but was predicted to be a part of the *nvf* cluster in this study, since SDRs are widely utilized in natural products biosynthesis. The sequences of the two genes indicated by * were revised in this study (see Supplementary Figs. 1 and 2).

Supplementary Table 2. Annotation of each protein of the *e-ana* cluster.

| Gene | Protein ID | Amino acids (base pairs) | Protein homologue, origin | Similarity/identity (%) | Proposed function |
|---|---|---|---|---|---|
| *e-anaPS* | 404104 | 2353 (7114) | AnaPS, *Neosartorya fischeri* | 94/91 | non-ribosomal peptide synthase |
| | | | AnaPS, *Aspergillus terreus* | 86/77 | |
| *e-anaPT* | 404100 | 437 (1365) | AnaPT, *Neosartorya fischeri* | 97/94 | dimethylallyl tryptophan synthase |
| | | | AnaPT, *Aspergillus terreus* | 83/73 | |
| *e-anaAT* | 386801 | 509 (1530) | AnaAT, *Neosartorya fischeri* | 93/89 | acetyltransferase |
| | | | AnaAT, *Aspergillus terreus* | 87/79 | |

Note: Protein IDs are as designated in JGI database.

Supplementary Table 3. Mutant strains of *Aspergillus novofumigatus* IBT16806 constructed in this study.

| Strain | Genotype |
|---|---|
| IBT35032 | *pyrG-* |
| IBT35033 | *pyrG-, ligD*Δ |
| IBT35034 | *pyrG-, ligD*Δ*, nvfA*Δ*::AfpyrG* |
| IBT35035 | *pyrG-, ligD*Δ*, nvfB*Δ*::AfpyrG* |
| IBT35036 | *pyrG-, ligD*Δ*, nvfC*Δ*::AfpyrG* |
| IBT35037 | *pyrG-, ligD*Δ*, nvfD*Δ*::AfpyrG* |
| IBT35038 | *pyrG-, ligD*Δ*, nvfE*Δ*::AfpyrG* |
| IBT35039 | *pyrG-, ligD*Δ*, nvfF*Δ*::AfpyrG* |
| IBT35040 | *pyrG-, ligD*Δ*, nvfG*Δ*::AfpyrG* |
| IBT35041 | *pyrG-, ligD*Δ*, nvfH*Δ*::AfpyrG* |
| IBT35042 | *pyrG-, ligD*Δ*, nvfI*Δ*::AfpyrG* |
| IBT35043 | *pyrG-, ligD*Δ*, nvfJ*Δ*::AfpyrG* |
| IBT35044 | *pyrG-, ligD*Δ*, nvfK*Δ*::AfpyrG* |
| IBT35045 | *pyrG-, ligD*Δ*, nvfL*Δ*::AfpyrG* |
| IBT35046 | *pyrG-, ligD*Δ*, nvfM*Δ*::AfpyrG* |
| IBT35047 | *pyrG-, ligD*Δ*, nvfD-G*Δ*::AfpyrG* |
| IBT35048 | *pyrG-, ligD*Δ*, nvfD-G*Δ*, e-anaPS*Δ*::AfpyrG* |

Supplementary Table 4. Transformants of *Aspergillus oryzae* NSAR1 constructed in this study.

| Strain | Host strain | Plasmids used for transformation |
|---|---|---|
| *A. oryzae/andM+nvfB* | *A. oryzae* NSAR1 | pTAex3-andM+nvfB |
| *A. oryzae/andM+nvfBKL* | *A. oryzae* NSAR1 | pTAex3-andM+nvfB, pUSA-nvfK+L |
| *A. oryzae/andM+nvfBKLC* | *A. oryzae* NSAR1 | pTAex3-andM+nvfB, pUSA-nvfK+L+C |
| *A. oryzae/andM+nvfBKLCHJM* | *A. oryzae* NSAR1 | pTAex3-andM+nvfB, pUSA-nvfK+L+C, pAdeA-nvfH+J+M |
| *A. oryzae/andM+nvfBKLCHJMI* | *A. oryzae/andM+nvfBKLCHJM* | pUNA-nvfI |
| *A. oryzae/andM+nvfBKLCHJMID* | *A. oryzae/andM+nvfBKLCHJM* | pUNA-nvfI+D |
| *A. oryzae/andM+nvfBKLCHJMIDG* | *A. oryzae/andM+nvfBKLCHJM* | pUNA-nvfI+D, pPTRI-nvfG |
| *A. oryzae/andM+nvfBKLCHJMIDGE* | *A. oryzae/andM+nvfBKLCHJM* | pUNA-nvfI+D, pPTRI-nvfG+E |
| *A. oryzae/andM+nvfBKLCHJMIDGEF* | *A. oryzae/andM+nvfBKLCHJMIDGE* | pBARI-nvfF |

Supplementary Table 5. NMR data for novofumigatonin (**1**).

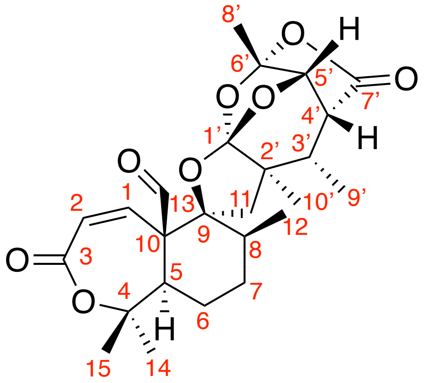


| | $^{13}C$ | $^{1}H$ | | |
|---|---|---|---|---|
| position | δ (ppm) | δ (ppm) | intensitiy | multiplicity |
| 1 | 144.5 | 6.83 | 1H | d (*J* = 13.2 Hz) |
| 2 | 123.7 | 6.20 | 1H | d (*J* = 13.2 Hz) |
| 3 | 164.6 | | | |
| 4 | 83.0 | | | |
| 5 | 49.8 | 2.18 | 1H | dd (*J* = 13.2, 2.5 Hz) |
| 6 | 18.7 | 1.73 (α) | 1H | m |
| | | 2.13 (β) | 1H | qd (*J* = 13.2, 3.9 Hz) |
| 7 | 28.1 | 1.85 (α) | 1H | m |
| | | 1.63 (β) | 1H | m |
| 8 | 39.7 | 2.21 | 1H | m |
| 9 | 90.1 | | | |
| 10 | 62.2 | | | |
| 11 | 49.4 | 1.72 (α) | 1H | d (*J* = 14.2 Hz) |
| | | 2.02 (β) | 1H | d (*J* = 13.7 Hz) |
| 12 | 16.4 | 0.94 | 3H | d (*J* = 7.3 Hz) |
| 13 | 201.3 | 10.25 | 1H | s |
| 14 | 30.4 | 1.34 | 3H | s |
| 15 | 23.7 | 1.24 | 3H | s |
| 1' | 128.2 | | | |
| 2' | 46.8 | | | |
| 3' | 38.2 | 2.04 | 1H | quin (*J* = 6.9 Hz) |
| 4' | 41.2 | 2.77 | 1H | dd (*J* = 8.8, 6.4 Hz) |
| 5' | 77.7 | 5.00 | 1H | d (*J* = 8.8 Hz) |
| 6' | 107.3 | | | |
| 7' | 175.1 | | | |
| 8' | 21.9 | 1.62 | 3H | s |
| 9' | 11.8 | 0.99 | 3H | d (*J* = 7.3 Hz) |
| 10' | 18.6 | 0.74 | 3H | s |

$^{1}H$ NMR: 800 MHz, $^{13}C$ NMR: 200 MHz (in DMSO-$d_6$)

Supplementary Table 6. NMR data for asnovolin A (**2**).

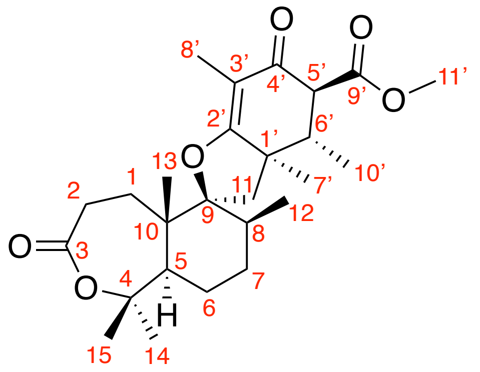


| | $^{13}C$ | $^{1}H$ | | |
|---|---|---|---|---|
| position | δ (ppm) | δ (ppm) | intensitiy | multiplicity |
| 1 | 31.8 | 1.80 | 2H | brt (*J* = 6.4 Hz) |
| 2 | 31.9 | 2.76 (α) | 1H | ddd (*J* = 15.7, 7.8, 4.9 Hz) |
| | | 2.68 (β) | 1H | ddd (*J* = 15.7, 7.3, 4.9 Hz) |
| 3 | 174.6 | | | |
| 4 | 85.4 | | | |
| 5 | 50.8 | 1.76 | 1H | brd (*J* = 11.3 Hz) |
| 6 | 21.2 | 1.72 (α) | 1H | m |
| | | 1.52 (β) | 1H | m |
| 7 | 29.2 | 1.61 (α) | 1H | m |
| | | 1.72 (β) | 1H | m |
| 8 | 39.8 | 1.82 | 1H | m |
| 9 | 98.3 | | | |
| 10 | 44.0 | | | |
| 11 | 43.4 | 2.09 (α) | 1H | d (*J* = 13.7 Hz) |
| | | 1.86 (β) | 1H | d (*J* = 13.7 Hz) |
| 12 | 17.2 | 1.09 | 3H | d (*J* = 7.3 Hz) |
| 13 | 16.9 | 1.35 | 3H | s |
| 14 | 24.3 | 1.46 | 3H | s |
| 15 | 33.1 | 1.47 | 3H | s |
| 1' | 44.7 | | | |
| 2' | 180.8 | | | |
| 3' | 108.8 | | | |
| 4' | 193.2 | | | |
| 5' | 58.0 | 3.25 | 1H | d (*J* = 12.2 Hz) |
| 6' | 41.7 | 2.43 | 1H | dq (*J* = 13.2, 6.9 Hz) |
| 7' | 22.0 | 1.21 | 3H | s |
| 8' | 8.0 | 1.72 | 3H | s |
| 9' | 171.5 | | | |
| 10' | 14.5 | 1.00 | 3H | d (*J* = 6.4 Hz) |
| 11' | 52.2 | 3.76 | 3H | s |

$^{1}$H NMR: 800 MHz, $^{13}$C NMR: 200 MHz (in $CDCl_3$)

Supplementary Table 7. NMR data for farnesyl-DMOA (**4**).

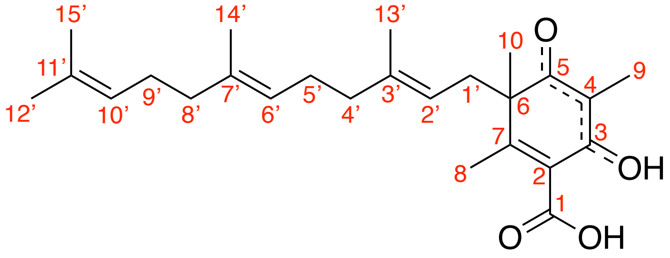


| | $^{13}C$ | $^{1}H$ | | |
|---|---|---|---|---|
| position | δ (ppm) | δ (ppm) | intensitiy | multiplicity |
| 1 | 170.5 | | | |
| 2 | 130.7 | | | |
| 3 | 185.2 | | | |
| 4 | 111.3 | | | |
| 5 | 178.8 | | | |
| 6 | 49.3 | | | |
| 7 | 162.2 | | | |
| 8 | 17.2 | 2.21 | 3H | s |
| 9 | 7.5 | 1.82 | 3H | s |
| 10 | 24.1 | 1.45 | 3H | s |
| 1' | 37.4 | 2.84 | 1H | dd (*J* = 14.4, 7.5 Hz) |
| | | 2.50 | 1H | dd (*J* = 14.5, 6.9 Hz) |
| 2' | 119.1 | 4.66 | 1H | brt (*J* = 6.9 Hz) |
| 3' | 139.8 | | | |
| 4' | 40.8 | 1.89 | 2H | m |
| 5' | 27.9 | 1.95 | 2H | m |
| 6' | 125.2 | 5.04 | 1H | brt (*J* = 6.9 Hz) |
| 7' | 136.0 | | | |
| 8' | 40.8 | 1.97 | 2H | m |
| 9' | 27.8 | 2.06 | 2H | m |
| 10' | 125.5 | 5.10 | 1H | tt (*J* = 6.9, 1.2 Hz) |
| 11' | 132.0 | | | |
| 12' | 25.9 | 1.69 | 3H | brs |
| 13' | 16.7 | 1.62 | 3H | brs |
| 14' | 16.1 | 1.57 | 3H | brs |
| 15' | 17.8 | 1.61 | 3H | brs |

$^{1}H$ NMR: 500 MHz, $^{13}C$ NMR: 125 MHz (in $CD_3OD$)

Supplementary Table 8. NMR data for asnovolin G (**18**).

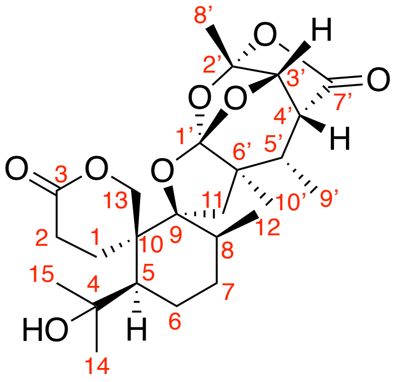


| | $^{13}C$ | $^{1}H$ | | |
|---|---|---|---|---|
| position | δ (ppm) | δ (ppm) | intensitiy | multiplicity |
| 1 | 27.6 | 2.07 (α) | 1H | m |
| | | 2.63 (β) | 1H | m |
| 2 | 29.5 | 2.57 (α) | 1H | m |
| | | 2.45 (β) | 1H | m |
| 3 | 174.8 | | | |
| 4 | 74.7 | | | |
| 5 | 52.8 | 1.43 | 1H | brd (*J* = 9.2 Hz) |
| 6 | 20.8 | 1.64 (α) | 1H | m |
| | | 1.38 (β) | 1H | m |
| 7 | 28.2 | 1.61 | 2H | m |
| 8 | 40.1 | 2.23 | 1H | brs |
| 9 | 92.7 | | | |
| 10 | 45.7 | | | |
| 11 | 50.0 | 2.11 (α) | 1H | m |
| | | 1.92 (β) | 1H | d (*J* = 13.9 Hz) |
| 12 | 15.7 | 1.06 | 3H | d (*J* = 7.3 Hz) |
| 13 | 67.2 | 4.65 (α) | 1H | d (*J* = 11.7 Hz) |
| | | 4.45 (β) | 1H | d (*J* = 11.4 Hz) |
| 14 | 27.0 | 1.30 | 3H | s |
| 15 | 35.1 | 1.32 | 3H | s |
| 1' | 128.5 | | | |
| 2' | 107.7 | | | |
| 3' | 78.1 | 4.78 | 1H | d (*J* = 8.8 Hz) |
| 4' | 42.1 | 2.68 | 1H | dd (*J* = 8.4, 6.2 Hz) |
| 5' | 40.0 | 2.13 | 1H | m |
| 6' | 48.0 | | | |
| 7' | 175.0 | | | |
| 8' | 22.5 | 1.76 | 3H | s |
| 9' | 12.1 | 1.18 | 3H | d (*J* = 7.3 Hz) |
| 10' | 19.1 | 1.04 | 3H | s |

$^{1}H$ NMR: 600 MHz, $^{13}C$ NMR: 150 MHz (in $CDCl_3$)

Supplementary Table 9. NMR data for asnovolin B (**23**).

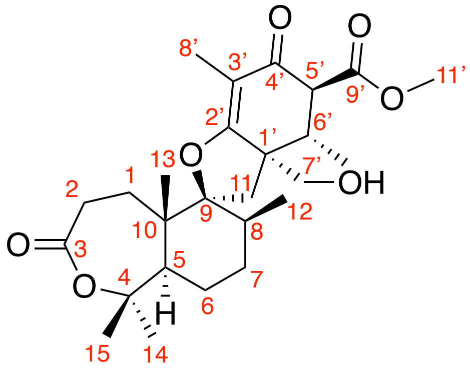


| | $^{13}C$ | $^{1}H$ | | |
|---|---|---|---|---|
| position | δ (ppm) | δ (ppm) | intensitiy | multiplicity |
| 1 | 31.4 | 1.78 | 2H | m |
| 2 | 31.8 | 2.76 (α) | 1H | ddd (*J* = 15.5, 9.8, 3.5 Hz) |
| | | 2.66 (β) | 1H | ddd (*J* = 15.5, 8.0, 3.5 Hz) |
| 3 | 174.8 | | | |
| 4 | 85.6 | | | |
| 5 | 50.8 | 1.79 | 1H | m |
| 6 | 21.2 | 1.73 (α) | 1H | m |
| | | 1.53 (β) | 1H | m |
| 7 | 29.2 | 1.63 (α) | 1H | m |
| | | 1.75 (β) | 1H | m |
| 8 | 39.8 | 1.89 | 1H | m |
| 9 | 97.8 | | | |
| 10 | 44.2 | | | |
| 11 | 38.0 | 2.29 (α) | 1H | d (*J* = 13.8 Hz) |
| | | 1.71(β) | 1H | d (*J* = 13.8 Hz) |
| 12 | 17.3 | 1.11 | 3H | d (*J* = 6.9 Hz) |
| 13 | 16.9 | 1.33 | 3H | s |
| 14 | 24.2 | 1.46 | 3H | s |
| 15 | 33.2 | 1.48 | 3H | s |
| 1' | 50.1 | | | |
| 2' | 175.4 | | | |
| 3' | 111.7 | | | |
| 4' | 193.9 | | | |
| 5' | 58.2 | 3.68 | 1H | d (*J* = 12.1 Hz) |
| 6' | 41.0 | 2.43 | 1H | dq (*J* = 12.1, 6.9 Hz) |
| 7' | 63.8 | 3.70 | 1H | d (*J* = 11.5 Hz) |
| | | 3.63 | 1H | d (*J* = 11.5 Hz) |
| 8' | 8.4 | 1.78 | 3H | s |
| 9' | 172.0 | | | |
| 10' | 15.4 | 1.12 | 3H | d (*J* = 6.9 Hz) |
| 11' | 52.3 | 3.76 | 3H | s |

$^{1}H$ NMR: 500 MHz, $^{13}C$ NMR: 125 MHz (in $CDCl_3$)

Supplementary Table 10. NMR data for asnovolin I (**5**).

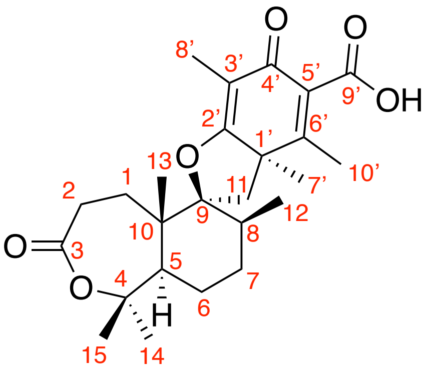


| | $^{13}C$ | $^{1}H$ | | | | | |
|---|---|---|---|---|---|---|---|
| position | δ (ppm) | δ (ppm) | intensitiy | multiplicity | HMBC correlation | COSY correlation | NOESY correlation |
| 1 | 34.0 | 1.92 (α) | 1H | m | 2, 9, 3 | H-2α, H-2β | H-2α, H-5 |
| | | 1.82 (β) | 1H | m | | H-2α, H-2β | H-2β, H-13 |
| 2 | 32.9 | 2.81 (α) | 1H | m | 1, 3 | H-1α, H-1β | H-1α, H-5, H-14 |
| | | 2.70 (β) | 1H | m | | H-1α, H-1β | H-1β, H-13, H-15 |
| 3 | 177.1 | | | | | | |
| 4 | 87.3 | | | | | | |
| 5 | 49.4 | 2.20 | 1H | brd (*J* = 14.2 Hz) | 4, 6, 7, 10 | H-6α, H-6β | H-1α, H-2α, H-8, H-14 |
| 6 | 22.9 | 1.54 (α) | 1H | m | | H-5 | |
| | | 1.87 (β) | 1H | m | | H-5 | H-13, H-15 |
| 7 | 30.3 | 1.85 (α) | 1H | m | | | |
| | | 1.50 (β) | 1H | m | 9, 12 | | H-12 |
| 8 | 37.9 | 1.90 | 1H | m | 7 | H-12 | H-5, H-12 |
| 9 | 102.8 | | | | | | |
| 10 | 44.5 | | | | | | |
| 11 | 37.0 | 2.30 | 2H | overlapped | 8, 9, 10, 1', 7' | | |
| 12 | 16.4 | 0.88 | 3H | d (*J* = 6.9 Hz) | 7, 8, 9 | H-8 | H-7β, H-8, H-13 |
| 13 | 17.8 | 1.41 | 3H | s | 1, 5, 9, 10 | | H-1β, H-2β, H-6β, H-12 |
| 14 | 31.5 | 1.54 | 3H | s | 4, 5, 15 | | H-2α, H-5 |
| 15 | 25.4 | 1.47 | 3H | s | 4, 5, 14 | | H-2β, H-6β |
| 1' | 51.1 | | | | | | |
| 2' | 183.6 | | | | | | |
| 3' | 108.8 | | | | | | |
| 4' | 188.0 | | | | | | |
| 5' | 129.7 | | | | | | |
| 6' | 164.5 | | | | | | |
| 7' | 36.0 | 1.65 | 3H | s | 11, 1', 2', 6' | | H-10' |
| 8' | 8.0 | 1.83 | 3H | s | 2', 3', 4' | | |
| 9' | 177.1 | | | | | | |
| 10' | 18.4 | 2.33 | 3H | s | 1', 5', 6' | | H-7' |

$^{1}H$ NMR: 800 MHz, $^{13}C$ NMR: 200 MHz (in $CD_3OD$)

Supplementary Table 11. NMR data for **6**.

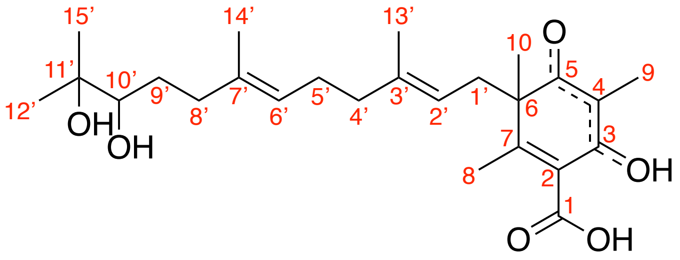


| | $^{13}$C | $^{1}$H | | | | |
|---|---|---|---|---|---|---|
| position | δ (ppm) | δ (ppm) | intensitiy | multiplicity | HMBC correlation | COSY correlation |
| 1 | 170.6 | | | | | |
| 2 | 130.9 | | | | | |
| 3 | 185.1* | | | | | |
| 4 | 111.3 | | | | | |
| 5 | 178.7* | | | | | |
| 6 | 49.3 | | | | | |
| 7 | 161.7 | | | | | |
| 8 | 17.1 | 2.20 | 3H | s | 2, 6, 7 | |
| 9 | 7.5 | 1.82 | 3H | s | 3, 4, 5 | |
| 10 | 24.1 | 1.45 | 3H | s | 5, 6, 7, 1' | |
| 1' | 37.4 | 2.84 | 1H | dd (*J* = 14.5, 7.6 Hz) | 6, 7, 10, 2', 3' | H-2' |
| | | 2.51 | 1H | dd (*J* = 14.5, 7.6 Hz) | 6, 7, 10, 2', 3' | H-2' |
| 2' | 119.1 | 4.67 | 1H | brt (*J* = 6.9 Hz) | 6, 1', 4', 13' | H-1', H-13' |
| 3' | 139.7 | | | | | |
| 4' | 40.8 | 1.90 | 2H | m | 2', 3', 5', 6', 13' | |
| 5' | 27.9 | 1.99 | 2H | m | 3', 4', 6', 7' | H-6' |
| 6' | 125.3 | 5.11 | 1H | brt (*J* = 6.9 Hz) | 4', 5', 8', 14' | H-5', H-14' |
| 7' | 136.2 | | | | | |
| 8' | 37.9 | 2.24 | 1H | m | 6', 7', 9' | H-9' |
| | | 2.00 | 1H | m | 6', 9', 14' | H-9' |
| 9' | 30.8 | 1.70 | 1H | m | 7', 8' | H-8', H-10' |
| | | 1.34 | 1H | m | 8', 10' | H-8', H-10' |
| 10' | 79.0 | 3.25 | 1H | dd (*J* = 10.5, 1.7 Hz) | 8', 9', 11', 12', 15' | H-9' |
| 11' | 73.8 | | | | | |
| 12' | 24.9 | 1.15 | 3H | brs | 10', 11', 15' | |
| 13' | 16.6 | 1.63 | 3H | brs | 2', 3', 4' | H-2' |
| 14' | 16.1 | 1.60 | 3H | s | 6', 7', 8' | H-6' |
| 15' | 25.6 | 1.19 | 3H | s | 10', 11', 12' | |

$^{1}$H NMR: 800 MHz, $^{13}$C NMR: 200 MHz (in $CD_3OD$)

Note: Two carbons indicated by * were identified in HMBC spectrum.

Supplementary Table 12. NMR data for asnovolin H (**8**).

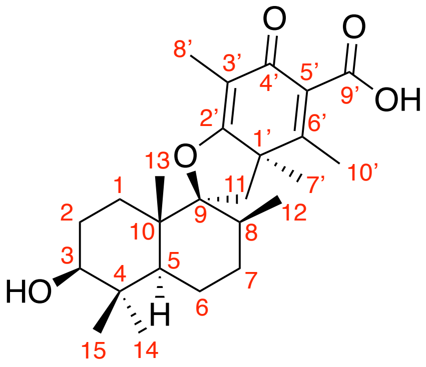


| | $^{13}C$ | $^{1}H$ | | | | | |
|---|---|---|---|---|---|---|---|
| position | δ (ppm) | δ (ppm) | intensitiy | multiplicity | HMBC correlation | COSY correlation | NOESY correlation |
| 1 | 32.9 | 1.52 (α) | 1H | m | | H-2α, H-2β | H-3 |
| | | 1.70 (β) | 1H | m | | | H-13 |
| 2 | 26.6 | 1.75 (α) | 1H | m | | H-1α | H-3 |
| | | 1.71 (β) | 1H | m | | H-1α | H-13 |
| 3 | 78.4 | 3.24 | 1H | dd (*J* = 10.9, 4.6 Hz) | | H-2α, H-2β | H-1α, H-2α, H-5, H-14 |
| 4 | 39.0 | | | | | | |
| 5 | 50.0 | 0.85 | 1H | m | 4, 6, 7 | H-6α, H-6β | H-3, H-6α, H-11α, H-14 |
| 6 | 16.7 | 1.52 (α) | 1H | m | | H-5 | H-5 |
| | | 1.57 (β) | 1H | m | | H-5 | |
| 7 | 29.0 | 1.52 (α) | 1H | m | | | |
| | | 1.61 (β) | 1H | m | | | H-12 |
| 8 | 43.1 | 1.52 | 1H | m | | H-12 | |
| 9 | 100.6 | | | | | | |
| 10 | 41.2 | | | | | | |
| 11 | 39.8 | 2.37 (α) | 1H | d (*J* = 13.2 Hz) | 8, 1', 7' | | H-5, H-7' |
| | | 1.89 (β) | 1H | d (*J* = 13.2 Hz) | 8, 1', 6', 7' | | H-12, H-10' |
| 12 | 16.8 | 1.12 | 3H | d (*J* = 6.3 Hz) | 7, 8, 9 | H-8 | H-7β, H-11β, H-13 |
| 13 | 17.3 | 1.24 | 3H | s | 1, 5, 9, 10 | | H-1β, H-2β, H-12, H-15 |
| 14 | 28.2 | 1.03 | 3H | s | 3, 4, 5, 15 | | H-3, H-5 |
| 15 | 15.1 | 0.86 | 3H | s | 3, 4, 5, 14 | | H-13 |
| 1' | 51.8 | | | | | | |
| 2' | 180.0 | | | | | | |
| 3' | 109.9 | | | | | | |
| 4' | 189.7 | | | | | | |
| 5' | 121.3 | | | | | | |
| 6' | 174.7 | | | | | | |
| 7' | 34.60 | 1.58 | 3H | s | 11, 1', 2', 6' | | H-11α, H-10' |
| 8' | 7.9 | 1.87 | 3H | s | 2', 3', 4' | | |
| 9' | 165.7 | | | | | | |
| 10' | 20.7 | 2.60 | 3H | s | 1', 5', 6' | | H-11β, H-7' |

$^{1}$H NMR: 500 MHz, $^{13}$C NMR: 125 MHz (in $CDCl_3$)

Supplementary Table 13. NMR data for chermesin D (**10**).

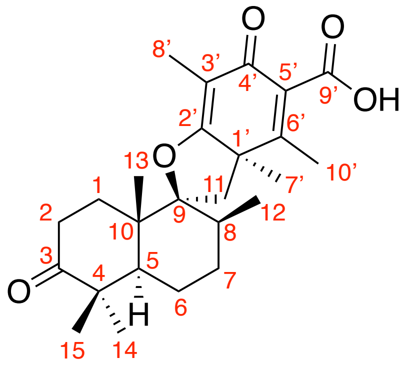


| | $^{13}C$ | $^{1}H$ | | | | | |
|---|---|---|---|---|---|---|---|
| position | δ (ppm) | δ (ppm) | intensitiy | multiplicity | HMBC correlation | COSY correlation | NOESY correlation |
| 1 | 34.0 | 1.86 (α) | 1H | td (*J* = 13.2, 4.6 Hz) | 13 | H-2α, H-2β | H-2α |
| | | 2.01 (β) | 1H | ddd (*J* = 13.2, 6.3, 2.9 Hz) | 3 | H-2α, H-2β | H-2α, H-2β, H-13, H-7' |
| 2 | 34.6 | 2.40 (α) | 1H | ddd (*J* = 15.5, 4.6, 2.9 Hz) | | H-1α, H-1β | H-1α, H-1β |
| | | 2.82 (β) | 1H | ddd (*J* = 15.5, 13.8, 6.3 Hz) | 1, 3 | H-1α, H-1β | H-1β, H-13 |
| 3 | 215.0 | | | | | | |
| 4 | 48.0 | | | | | | |
| 5 | 51.2 | 1.30 | 1H | dd (*J* = 12.1, 2.3 Hz) | 4, 7, 10, 13, 14, 15 | H-6α, H-6β | H-6α, H-7α, H-11α |
| 6 | 17.8 | 1.48 (α) | 1H | m | | H-5, H-7α, H-7β | H-5 |
| | | 1.72 (β) | 1H | qd (*J* = 12.6, 2.9 Hz) | 8 | H-5, H-7α, H-7β | H-13 |
| 7 | 28.7 | 1.56 (α) | 1H | m | 6, 9 | H-6α, H-6β | H-5 |
| | | 1.68 (β) | 1H | m | 5, 9 | H-6α, H-6β | H-12 |
| 8 | 42.9 | 1.58 | 1H | m | 12 | H-12 | H-11β |
| 9 | 99.9 | | | | | | |
| 10 | 40.9 | | | | | | |
| 11 | 39.7 | 2.32 (α) | 1H | d (*J* = 13.2 Hz) | 8, 9, 1', 2', 7' | | H-5, H-7' |
| | | 1.92 (β) | 1H | d (*J* = 13.2 Hz) | 8, 9, 1', 2', 6' | | H-8, H-12, H-10' |
| 12 | 16.7 | 1.17 | 3H | d (*J* = 7.5 Hz) | 7, 8, 9 | H-8 | H-7β, H-11β, H-13 |
| 13 | 16.6 | 1.47 | 3H | s | 1, 5, 9, 10 | | H-1β, H-2β, H-6β, H-12 |
| 14 | 25.3* | 1.12 | 3H | s | 3, 4, 5, 15 | | |
| 15 | 21.7* | 1.12 | 3H | s | 3, 4, 5, 14 | | |
| 1' | 51.6 | | | | | | |
| 2' | 179.3 | | | | | | |
| 3' | 110.2 | | | | | | |
| 4' | 189.8 | | | | | | |
| 5' | 121.4 | | | | | | |
| 6' | 174.5 | | | | | | |
| 7' | 34.6 | 1.57 | 3H | s | 11, 1', 2', 6' | | H-1β, H-11α, H-10' |
| 8' | 8.0 | 1.89 | 3H | s | 2', 3', 4' | | |
| 9' | 165.5 | | | | | | |
| 10' | 20.7 | 2.58 | 3H | s | 1', 5', 6' | | H-11β, H-7' |

$^{1}H$ NMR: 500 MHz, $^{13}C$ NMR: 125 MHz (in $CDCl_3$)

Note: Two carbons indicated by * are interchangeable.

Supplementary Table 14. NMR data for asnovolin J (**11**).

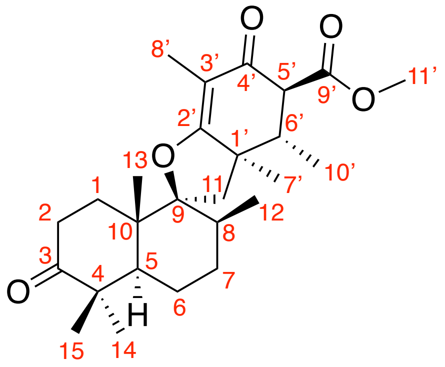


| | $^{13}C$ | $^{1}H$ | | | | | |
|---|---|---|---|---|---|---|---|
| position | δ (ppm) | δ (ppm) | intensitiy | multiplicity | HMBC correlation | COSY correlation | NOESY correlation |
| 1 | 33.8 | 1.82 (α) | 1H | td (*J* = 13.2, 4.9 Hz) | 2, 10, 13 | H-2α, H-2β | H-2α, H-11α |
| | | 1.90 (β) | 1H | ddd (*J* = 13.2, 5.9, 2.9 Hz) | 5 | H-2α, H-2β | H-2α, H-2β, H-13, H-7' |
| 2 | 34.1 | 2.35 (α) | 1H | dt (*J* = 15.2, 3.9 Hz) | | H-1α, H-1β | H-1α, H-1β |
| | | 2.77 (β) | 1H | td (*J* = 14.2, 5.9 Hz) | 1 | H-1α, H-1β | H-1β, H-13 |
| 3 | 215.5 | | | | | | |
| 4 | 48.0 | | | | | | |
| 5 | 51.1 | 1.28 | 1H | dd (*J* = 12.2, 2.0 Hz) | 4, 7, 10, 13, 14, 15 | H-6α, H-6β | H-6α, H-7α, H-11α |
| 6 | 17.1 | 1.45 (α) | 1H | m | | H-5, H-7β | H-5 |
| | | 1.69 (β) | 1H | m | 8 | H-5, H-7α, H-7β | |
| 7 | 29.0 | 1.61 (α) | 1H | m | | H-6β | H-5 |
| | | 1.68 (β) | 1H | m | | H-6α, H-6β | H-12 |
| 8 | 42.8 | 1.78 | 1H | m | 9, 12 | H-12 | H-12, H-6' |
| 9 | 97.9 | | | | | | |
| 10 | 41.0 | | | | | | |
| 11 | 45.2 | 2.14 (α) | 1H | d (*J* = 13.7 Hz) | 8, 9, 10, 1', 2', 6', 7' | | H-1α, H-5, H-7' |
| | | 1.73 (β) | 1H | d (*J* = 13.7 Hz) | 8, 1', 6', 7' | | H-6', H-10' |
| 12 | 17.8 | 1.19 | 3H | d (*J* = 7.6 Hz) | 7, 8, 9 | H-8 | H-7β, H-8, H-13 |
| 13 | 16.6 | 1.43 | 3H | s | 1, 5, 9, 10 | | H-1β, H-2β, H-12 |
| 14 | 25.3* | 1.10 | 3H | s | 3, 4, 5, 15 | | |
| 15 | 21.63* | 1.10 | 3H | s | 3, 4, 5, 14 | | |
| 1' | 44.9 | | | | | | |
| 2' | 180.3 | | | | | | |
| 3' | 109.4 | | | | | | |
| 4' | 193.3 | | | | | | |
| 5' | 58.0 | 3.25 | 1H | d (*J* = 12.5 Hz) | 4', 6', 9', 10' | H-6' | H-7', H-10' |
| 6' | 42.5 | 2.37 | 1H | m | 1', 5', 7', 9', 10' | H-5', H-10' | H-8, H-11β, H-10' |
| 7' | 21.60 | 1.18 | 3H | s | 11, 1', 2', 6' | | H-1β, H-11α, H-5', H-10' |
| 8' | 8.0 | 1.74 | 3H | s | 2', 3', 4' | | |
| 9' | 171.5 | | | | | | |
| 10' | 14.5 | 0.99 | 3H | d (*J* = 6.9 Hz) | 1', 5', 6' | H-6' | H-11β, H-5', H-6', H-7', H-11' |
| 11' | 52.2 | 3.77 | 3H | s | 9' | | H-10' |

$^{1}H$ NMR: 800 MHz, $^{13}C$ NMR: 200 MHz (in $CDCl_3$)

Note: Two carbons indicated by * are interchangeable.

Supplementary Table 15. NMR data for asnovolin K (**12**).

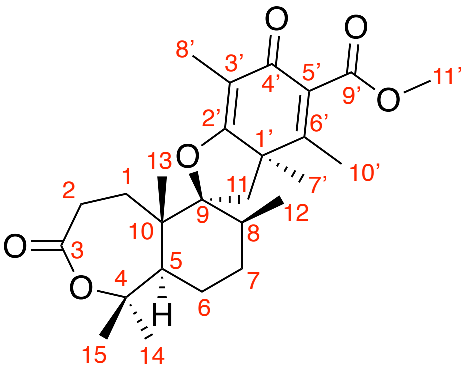


| position | $^{13}C$ | $^{1}H$ | | | | | |
|---|---|---|---|---|---|---|---|
| | δ (ppm) | δ (ppm) | intensitiy | multiplicity | HMBC correlation | COSY correlation | NOESY correlation |
| 1 | 32.0 | 1.81 (α) | 1H | m | 10, 13 | H-2α, H-2β | H-5 |
| | | 1.90 (β) | 1H | m | 2, 3, 5, 10, 13 | H-2α, H-2β | H-13, H-7' |
| 2 | 31.9 | 2.70 (α) | 1H | m | 1, 3, 10 | H-1α, H-1β | H-14 |
| | | 2.78 (β) | 1H | m | 1, 3, 10 | H-1α, H-1β | H-13, H-15 |
| 3 | 174.6 | | | | | | |
| 4 | 85.5 | | | | | | |
| 5 | 50.5 | 1.80 | 1H | overlapped | 4, 6, 10, 14, 15 | H-6α, H-6β | H-1α, H-6α, H-7α, H-11α, H-14 |
| 6 | 21.3 | 1.51 (α) | 1H | m | 7, 8, 10 | H-5, H-7β | H-5 |
| | | 1.74 (β) | 1H | m | 5, 7, 10 | H-5, H-7α | H-13 |
| 7 | 29.0 | 1.57 (α) | 1H | m | 6, 9, 12 | H-6β | H-5, H-12 |
| | | 1.66 (β) | 1H | m | 6, 8, 9, 12 | H-6α | H-12 |
| 8 | 38.9 | 1.69 | 1H | m | 6, 7, 9, 10, 12 | H-12 | H-11β, H-12 |
| 9 | 98.8 | | | | | | |
| 10 | 43.8 | | | | | | |
| 11 | 38.0 | 2.11 (α) | 1H | d (*J* = 13.2 Hz) | 8, 9, 10, 1', 2', 7' | | H-5, H-7' |
| | | 1.95 (β) | 1H | d (*J* = 13.2 Hz) | 8, 9, 10, 1', 7' | | H-8 |
| 12 | 16.4 | 0.97 | 3H | d (*J* = 7.3 Hz) | 7, 8, 9 | H-8 | H-7α, H-7β, H-8, H-13 |
| 13 | 16.9 | 1.38 | 3H | s | 1, 5, 9, 10 | | H-1β, H-2β, H-6β, H-12 |
| 14 | 32.9* | 1.48 | 3H | s | 4, 5, 15 | | H-2α, H-5 |
| 15 | 24.3* | 1.47 | 3H | s | 4, 5, 14 | | H-2β |
| 1' | 48.1 | | | | | | |
| 2' | 177.8 | | | | | | |
| 3' | 109.9 | | | | | | |
| 4' | 184.3 | | | | | | |
| 5' | 131.8 | | | | | | |
| 6' | 155.3 | | | | | | |
| 7' | 34.3 | 1.56 | 3H | s | 11, 1', 2', 6' | | H-1β, H-11α, H-10' |
| 8' | 7.9 | 1.81 | 3H | s | 2', 3', 4' | | |
| 9' | 167.6 | | | | | | |
| 10' | 16.9 | 2.03 | 3H | s | 1', 5', 6' | | H-7' |
| 11' | 52.3 | 3.85 | 3H | s | 9' | | |

$^{1}H$ NMR: 800 MHz, $^{13}C$ NMR: 200 MHz (in $CDCl_3$)

Note: Two carbons indicated by * are interchangeable.

Supplementary Table 16. NMR data for fumigatonoid A (**14**) in $CDCl_3$.

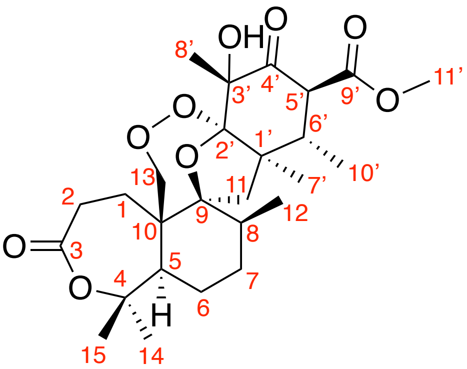


| | $^{13}C$ | $^{1}H$ | | | | | |
|---|---|---|---|---|---|---|---|
| position | δ (ppm) | δ (ppm) | intensitiy | multiplicity | HMBC correlation | COSY correlation | NOESY correlation |
| 1 | 30.6 | 1.54 (α) | 1H | m | 2, 9, 10, 13 | H-2, H-13β | H-11α |
| | | 2.04 (β) | 1H | m | 2, 5, 10, 13 | H-2 | H-11α, H-13α |
| 2 | 32.6 | 2.56 | 2H | m | 1, 3, 10 | H-1α, H-1β | |
| 3 | 172.7 | | | | | | |
| 4 | 84.4 | | | | | | |
| 5 | 46.4 | 2.16 | 1H | dd (*J* = 13.2, 2.0 Hz) | 4, 6, 10, 13, 14, 15 | H-6α, H-6β | H-6α, H-8, H-14 |
| 6 | 21.8 | 1.31 (α) | 1H | m | 8, 10 | H-5, H-7α, H-7β | H-5, H-7α |
| | | 1.82 (β) | 1H | m | 5, 7, 10 | H-5, H-7α, H-7β | H-7β, H-13β |
| 7 | 29.8 | 1.68 (α) | 1H | m | 6, 8, 9 | H-6α, H-6β | H-6α, H-12 |
| | | 1.47 (β) | 1H | m | 6, 8, 12 | H-6α, H-6β, H-8 | H-6β, H-12 |
| 8 | 31.6 | 1.70 | 1H | m | 7, 10, 12 | H-7β, H-12 | H-5, H-12 |
| 9 | 93.8 | | | | | | |
| 10 | 50.7 | | | | | | |
| 11 | 43.3 | 2.31 (α) | 1H | d (*J* = 13.2 Hz) | 8, 9, 10, 1', 6', 7' | | H-1α, H-1β, H-7' |
| | | 2.08 (β) | 1H | d (*J* = 13.2 Hz) | 8, 9, 10, 1', 2', 6' | | H-12, H-6', H-10' |
| 12 | 17.8 | 0.95 | 3H | d (*J* = 6.4 Hz) | 7, 8, 9 | H-8 | H-7α, H-7β, H-8, H-11β, H-6' |
| 13 | 80.6 | 4.49 (α) | 1H | d (*J* = 15.2 Hz) | 1, 9 | | H-1β, H-15 |
| | | 4.80 (β) | 1H | brd (*J* = 14.7 Hz) | 1, 5, 10 | H-1α | H-6β, H-15, H-8' |
| 14 | 30.0 | 1.46 | 3H | s | 4, 5, 15 | | H-5 |
| 15 | 26.8 | 1.32 | 3H | s | 3, 4, 5, 14 | | H-13α, H-13β |
| 1' | 48.6 | | | | | | |
| 2' | 115.1 | | | | | | |
| 3' | 77.4 | | | | | | |
| 4' | 201.2 | | | | | | |
| 5' | 54.5 | 4.06 | 1H | d (*J* = 13.2 Hz) | 1', 4', 6', 9', 10' | H-6' | H-7', H-10' |
| 6' | 37.3 | 2.51 | 1H | m | 11, 1', 4', 5', 7', 9', 10' | H-5', H-10' | H-11β, H-12, H-10' |
| 7' | 16.0 | 1.30 | 3H | s | 11, 1', 2', 6' | | H-11α, H-5', H-10' |
| 8' | 18.3 | 1.42 | 3H | s | 2', 3', 4' | | H-13β |
| 9' | 169.8 | | | | | | |
| 10' | 14.1 | 1.01 | 3H | d (*J* = 6.9 Hz) | 1', 5', 6' | H-6' | H-11β, H-5', H-6', H-7' |
| 11' | 52.0 | 3.75 | 3H | s | 9' | | |
| 3'-OH | | 2.44 | 1H | brs | | | |

$^{1}H$ NMR: 800 MHz, $^{13}C$ NMR: 200 MHz (in $CDCl_3$)

Supplementary Table 17. NMR data for fumigatonoid A (**14**) in DMSO-$d_6$.

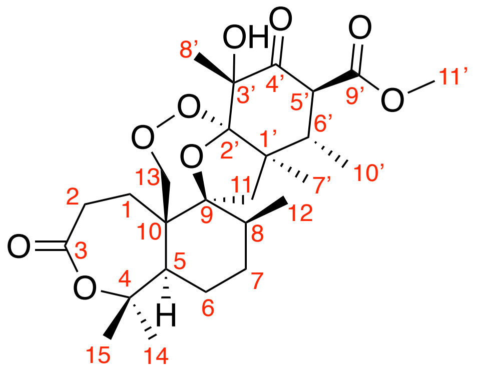

| | $^{13}C$ | $^{1}H$ | | | | | |
|---|---|---|---|---|---|---|---|
| position | δ (ppm) | δ (ppm) | intensitiy | multiplicity | HMBC correlation | COSY correlation | NOESY correlation |
| 1 | 30.0 | 1.53 (α) | 1H | m | 2, 3, 9, 10, 13 | H-2α, H-2β, H-13β | |
| | | 1.75 (β) | 1H | td (*J* = 13.8, 4.4 Hz) | 2, 5, 10, 13 | H-2α, H-2β | H-13α |
| 2 | 32.0 | 2.77 (α) | 1H | td (*J* = 13.5, 6.5 Hz) | 1, 3, 10 | H-1α, H-1β | H-14 |
| | | 2.28 (β) | 1H | m | 3 | H-1α, H-1β | |
| 3 | 172.7 | | | | | | |
| 4 | 83.9 | | | | | | |
| 5 | 44.4 | 2.29 | 1H | m | 4, 6, 7, 13, 14, 15 | H-6α, H-6β | H-6α, H-14 |
| 6 | 21.1 | 1.31 (α) | 1H | m | 7, 8, 10 | H-5, H-7α, H-7β | H-5 |
| | | 1.62 (β) | 1H | m | 5, 8, 10 | H-5, H-7β | H-13β |
| 7 | 29.6 | 1.64 (α) | 1H | m | 6, 9 | H-6α, H-8 | |
| | | 1.18 (β) | 1H | m | | H-6α, H-6β, H-8 | H-12 |
| 8 | 30.5 | 1.83 | 1H | m | 7, 12 | H-7α, H-7β, H-12 | |
| 9 | 93.1 | | | | | | |
| 10 | 50.2 | | | | | | |
| 11 | 43.2 | 2.21 (α) | 1H | d (*J* = 13.5 Hz) | 8, 9, 10, 1', 6', 7' | | |
| | | 2.04 (β) | 1H | d (*J* = 13.5 Hz) | 8, 9, 10, 1', 2', 6' | | H-12, H-10' |
| 12 | 17.8 | 0.82 | 3H | d (*J* = 6.8 Hz) | 7, 8, 9 | H-8 | H-7β, H-11β, H-6' |
| 13 | 79.9 | 4.21 (α) | 1H | d (*J* = 14.7 Hz) | 1, 9 | | H-1β, H-15 |
| | | 4.66 (β) | 1H | brd (*J* = 14.7 Hz) | 1, 5, 10 | H-1α | H-6β, H-15 |
| 14 | 29.3 | 1.39 | 3H | s | 4, 5, 15 | | H-1α, H-5 |
| 15 | 26.6 | 1.18 | 3H | s | 3, 4, 5, 14 | | H-13α, H-13β |
| 1' | 47.7 | | | | | | |
| 2' | 114.7 | | | | | | |
| 3' | 76.0 | | | | | | |
| 4' | 202.5 | | | | | | |
| 5' | 53.7 | 4.05 | 1H | d (*J* = 12.9 Hz) | 1', 4', 6', 9', 10' | H-6' | H-7', H-10' |
| 6' | 36.8 | 2.34 | 1H | dq (*J* = 12.7, 6.4 Hz) | 11, 1', 4', 5', 7', 9', 10' | H-5', H-10' | H-12, H-10' |
| 7' | 15.9 | 1.20 | 3H | s | 11, 1', 2', 6' | | H-5', H-10', 3'-OH |
| 8' | 17.2 | 1.23 | 3H | s | 2', 3', 4' | | |
| 9' | 169.7 | | | | | | |
| 10' | 13.8 | 0.90 | 3H | d (*J* = 6.5 Hz) | 1', 5', 6' | H-6' | H-11β, H-5', H-6', H-7' |
| 11' | 51.6 | 3.63 | 3H | s | 9' | | |
| 3'-OH | | 6.00 | 1H | s | 2', 3', 4', 8' | | H-7' |

$^{1}H$ NMR: 800 MHz, $^{13}C$ NMR: 200 MHz (in DMSO-$d_6$)

Supplementary Table 18. NMR data for fumigatonoid B (**15**).

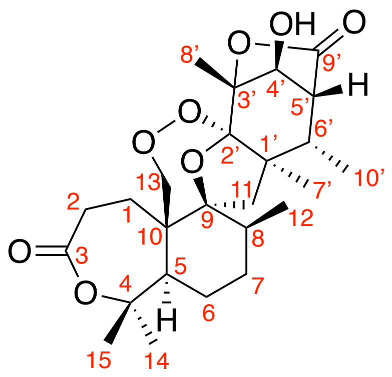

| | $^{13}$C | $^{1}$H | | | | | |
|---|---|---|---|---|---|---|---|
| position | δ (ppm) | δ (ppm) | intensitiy | multiplicity | HMBC correlation | COSY correlation | NOESY correlation |
| 1 | 30.5 | 1.51 (α) | 1H | m | 2, 10 | H-2, H-13β | H-11α |
| | | 2.02 (β) | 1H | dt (*J* = 13.7, 9.3 Hz) | 2, 5, 10, 13 | H-2 | H-11α, H-13α |
| 2 | 32.5 | 2.56 | 2H | m | 1, 3, 10 | H-1α, H-1β | |
| 3 | 172.4 | | | | | | |
| 4 | 84.0 | | | | | | |
| 5 | 46.2 | 2.21 | 1H | dd (*J* = 12.2, 2.5 Hz) | 4, 6, 13, 14, 15 | H-6α | H-6α, H-14 |
| 6 | 21.8 | 1.41 (α) | 1H | m | 8, 10 | H-5 | H-5 |
| | | 1.86 (β) | 1H | m | | | H-13β |
| 7 | 29.8 | 1.86 (α) | 1H | m | | | |
| | | 1.43 (β) | 1H | m | 6, 12 | | H-12, 4'-OH |
| 8 | 31.4 | 1.86 | 1H | m | | | |
| 9 | 96.4 | | | | | | |
| 10 | 50.6 | | | | | | |
| 11 | 44.8 | 2.22 (α) | 1H | d (*J* = 13.7 Hz) | 8, 9, 10, 1', 6', 7' | | H-1α, H-1β, H-7' |
| | | 2.16 (β) | 1H | d (*J* = 13.7 Hz) | 8, 9, 10, 1', 2', 6' | | H-12, H-6' |
| 12 | 16.7 | 1.13 | 3H | d (*J* = 6.4 Hz) | 7, 8, 9 | | H-7β, H-11β,4'-OH |
| 13 | 80.6 | 4.52 (α) | 1H | d (*J* = 15.2 Hz) | 1, 9, 10 | | H-1β, H-15 |
| | | 4.79 (β) | 1H | dd (*J* = 15.2, 2.0 Hz) | 1, 5, 10 | H-1α | H-6β, H-15, H-8' |
| 14 | 30.0 | 1.47 | 3H | s | 4, 5, 15 | | H-5 |
| 15 | 26.7 | 1.34 | 3H | s | 3, 4, 5, 14 | | H-13α, H-13β |
| 1' | 49.3 | | | | | | |
| 2' | 113.3 | | | | | | |
| 3' | 82.6 | | | | | | |
| 4' | 77.4 | 4.06 | 1H | dd (*J* = 8.8, 4.9 Hz) | 2', 3', 6', 8' | H-5', 4'-OH | H-5', H-8' |
| 5' | 48.9 | 2.43 | 1H | dd (*J* = 4.9, 1.5 Hz) | 1', 4', 6', 9', 10' | H-4' | H-4' |
| 6' | 31.5 | 2.45 | 1H | qd (*J* = 6.9, 1.5 Hz) | 11, 1', 4', 5', 7', 9', 10' | H-10' | H-11β, 4'-OH |
| 7' | 17.0 | 1.17 | 3H | s | 11, 1', 2', 6' | | H-11α |
| 8' | 17.6 | 1.59 | 3H | s | 2', 3', 4' | | H-13β, H-4' |
| 9' | 173.8 | | | | | | |
| 10' | 13.5 | 1.11 | 3H | d (*J* = 7.3 Hz) | 1', 5', 6' | H-6' | |
| 4'-OH | | 4.17 | 1H | d (*J* = 8.8 Hz) | 3' | H-4' | H-6', H-7β, H-12 |

$^{1}$H NMR: 800 MHz, $^{13}$C NMR: 200 MHz (in $CDCl_3$)

Supplementary Table 19. NMR data for **16**.

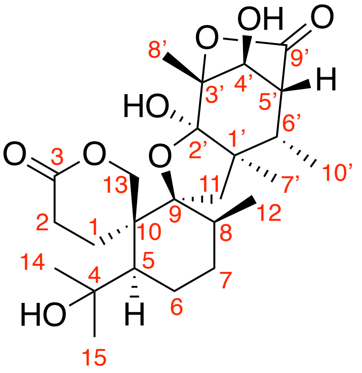


| | $^{13}C$ | $^{1}H$ | | | | | |
|---|---|---|---|---|---|---|---|
| position | δ (ppm) | δ (ppm) | intensitiy | multiplicity | HMBC correlation | COSY correlation | NOESY correlation |
| 1 | 26.4 | 2.71 (α) | 1H | m | 2, 3, 5, 9, 10 | H-2α,H-2β | H-2α, H-11α |
| | | 2.36 (β) | 1H | m | 2, 5, 9, 10 | H-2α,H-2β | H-5, H-11α, H-15, 4-OH |
| 2 | 29.9 | 2.05 (α) | 1H | dd (*J* = 15.7, 6.9 Hz) | 1, 3, 10, 13 | H-1α, H-1β | H-1α |
| | | 2.60 (β) | 1H | dd (*J* = 15.7, 9.8 Hz) | 1, 3 | H-1α, H-1β | H-13β |
| 3 | 174.1 | | | | | | |
| 4 | 73.3 | | | | | | |
| 5 | 54.0 | 1.24 | 1H | overlapped | 4, 6, 7, 9, 10, 13, 14, 15 | H-6β | H-1β, H-7α, H-11α, H-11β |
| 6 | 20.2 | 1.22 (α) | 1H | m | | H-7α, H-7β | H-7α |
| | | 1.49 (β) | 1H | m | 7 | H-5, H-7α, H-7β | H-12, H-13α, H-15 |
| 7 | 29.0 | 1.55 (α) | 1H | m | 5 | H-6α, H-6β, H-8 | H-5, H-6α, H-8, H-11β |
| | | 1.39 (β) | 1H | m | 5, 9 | H-6α, H-6β, H-8 | H-8 |
| 8 | 39.3* | 2.41 | 1H | m | 6, 7, 9, 10, 12 | H-7α, H-7β, H-12 | H-7α, H-7β,H-11β, H-12, H-6' |
| 9 | 93.7 | | | | | | |
| 10 | 46.2 | | | | | | |
| 11 | 49.4 | 1.81 (α) | 1H | d (*J* = 13.7 Hz) | 8, 9, 10, 1', 6', 7' | | H-1α, H-1β, H-5 |
| | | 1.98 (β) | 1H | d (*J* = 13.7 Hz) | 8, 10, 1', 2', 6' | | H-5, H-7α, H-8, H-6', H-10' |
| 12 | 16.2 | 0.91 | 3H | d (*J* = 7.3 Hz) | 7, 8, 9 | H-8 | H-6β, H-8, H-13α |
| 13 | 67.2 | 4.48 (α) | 1H | d (*J* = 11.7 Hz) | 1, 3, 5, 10 | | H-6β, H-12, H-15 |
| | | 4.19 (β) | 1H | d (*J* = 12.2 Hz) | 1, 3, 5, 10 | | H-2β |
| 14 | 34.5 | 1.16 | 3H | s | 4, 5, 15 | | |
| 15 | 25.8 | 1.11 | 3H | s | 4, 5, 14 | | H-1β, H-6β, H-13α |
| 1' | 49.7 | | | | | | |
| 2' | 104.4 | | | | | | |
| 3' | 87.4 | | | | | | |
| 4' | 73.7 | 3.99 | 1H | d (*J* = 5.4 Hz) | 2', 3',5', 6', 8' | H-5', 4'-OH | H-5', H-8' |
| 5' | 48.2 | 2.18 | 1H | dd (*J* = 4.9, 1.5 Hz) | 1', 3', 4', 6', 9', 10' | H-4', H-6' | H-4', H-6' |
| 6' | 33.3 | 2.58 | 1H | m | 11, 1', 4', 5', 7', 9', 10' | H-5', H-10' | H-8, H-11β, H-5', H-10' |
| 7' | 18.8 | 0.93 | 3H | s | 11, 1', 2', 6' | | |
| 8' | 17.2 | 1.48 | 3H | s | 2', 3', 4' | | H-4' |
| 9' | 175.0 | | | | | | |
| 10' | 15.2 | 0.99 | 3H | d (*J* = 6.9 Hz) | 1', 5', 6' | H-6' | H-11β, H-6' |
| 4-OH | | 4.12 | 1H | s | 4, 5, 14, 15 | | H-1β |
| 2'-OH | | 5.19 | 1H | brs | 1', 2' | | |
| 4'-OH | | 5.82 | 1H | brs | | H-4' | |

$^{1}H$ NMR: 800 MHz, $^{13}C$ NMR: 200 MHz (in DMSO-$d_6$)

Note: The carbon indicated by * was identified from HSQC spectrum.

Supplementary Table 20. NMR data for novofumigatonol (**19**).

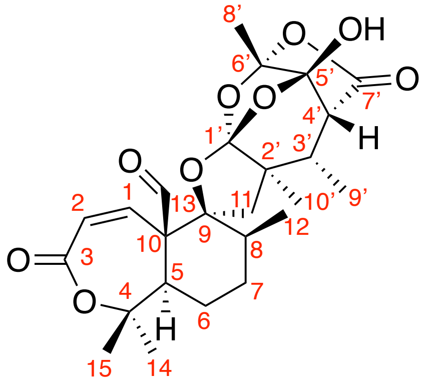

| position | $^{13}C$ | $^{1}H$ | | | | | |
|---|---|---|---|---|---|---|---|
| | δ (ppm) | δ (ppm) | intensitiy | multiplicity | HMBC correlation | COSY correlation | NOESY correlation |
| 1 | 145.7 | 7.03 | 1H | d (*J* = 13.2 Hz) | 3, 5, 9, 13 | H-2 | H-2, H-11α, H-13, H-15, H-10' |
| 2 | 125.5 | 6.28 | 1H | d (*J* = 13.2 Hz) | 3, 10 | H-1 | H-1, H-15, H-10' |
| 3 | 167.9 | | | | | | |
| 4 | 85.6 | | | | | | |
| 5 | 51.8 | 2.27 | 1H | dd (*J* = 13.2, 2.5 Hz) | 4, 6, 10, 13 | H-6α | H-6α, H-11α, H-14 |
| 6 | 20.4 | 1.88 (α) | 1H | m | | H-5, H-7α, H-7β | H-5, H-14 |
| | | 2.21 (β) | 1H | qd (*J* = 13.2, 3.9 Hz) | | H-7α, H-7β | H-7β, H-12, H-13, H-15 |
| 7 | 30.0 | 1.93 (α) | 1H | m | | H-6α, H-6β, H-8 | H-8 |
| | | 1.80 (β) | 1H | m | 5, 9 | H-6α, H-6β, H-8 | H-6β, H-8, H-12 |
| 8 | 42.0 | 2.38 | 1H | m | | H-7α, H-7β, H-12 | H-7α, H-7β, H-11β, H-12 |
| 9 | 92.1 | | | | | | |
| 10 | 64.5 | | | | | | |
| 11 | 51.3 | 1.93 (α) | 1H | d (*J* = 14.2 Hz) | 8, 9, 10, 1', 2', 3', 10' | | H-1, H-5, H-10' |
| | | 2.12 (β) | 1H | d (*J* = 13.7 Hz) | 8, 9, 10, 1', 2', 3', 10' | | H-8, H-3', H-9' |
| 12 | 16.9 | 1.10 | 3H | d (*J* = 7.3 Hz) | 7, 8, 9 | H-8 | H-6β, H-7β, H-8, H-13 |
| 13 | 201.0 | 10.24 | 1H | s | 1, 10 | | H-1, H-6β, H-12, H-15 |
| 14 | 31.2 | 1.38 | 3H | s | 4, 5, 15 | | H-5, H-6α |
| 15 | 24.8 | 1.49 | 3H | s | 4, 5, 14 | | H-1, H-2, H-6β, H-13 |
| 1' | 127.1 | | | | | | |
| 2' | 47.8 | | | | | | |
| 3' | 41.9 | 2.31 | 1H | quin (*J* = 6.9 Hz) | 2', 4', 7', 9' | H-4', H-9' | H-11β, H-4', H-9' |
| 4' | 49.9 | 2.63 | 1H | d (*J* = 6.4 Hz) | 2', 3', 5', 6', 7' | H-3' | H-3', H-8', H-9' |
| 5' | 105.4* | | | | | | |
| 6' | 109.4* | | | | | | |
| 7' | 175.9 | | | | | | |
| 8' | 18.6 | 1.59 | 3H | s | 5', 6' | | H-4' |
| 9' | 12.4 | 1.16 | 3H | d (*J* = 7.3 Hz) | 2', 3', 4' | H-3' | H-11β, H-3', H-4', H-10' |
| 10' | 19.9 | 0.90 | 3H | s | 11, 1', 2', 3' | | H-1, H-2, H-11α, H-9' |

$^{1}H$ NMR: 800 MHz, $^{13}C$ NMR: 200 MHz (in $CD_3OD$)

Note: Two carbons indicated by * are interchangeable.

Supplementary Table 21. NMR data for **21**.

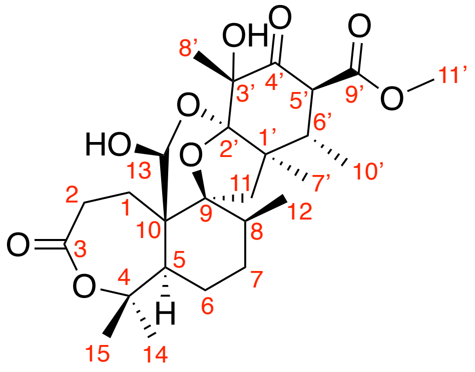

| position | $^{13}C$ | $^{1}H$ | | | | | |
|---|---|---|---|---|---|---|---|
| | δ (ppm) | δ (ppm) | intensitiy | multiplicity | HMBC correlation | COSY correlation | NOESY correlation |
| 1 | 33.8 | 1.94 (α) | 1H | m | | H-2 | |
| | | 1.86 (β) | 1H | m | 2, 13 | H-2 | H-13 |
| 2 | 30.0 | 2.42 | 2H | m | 1, 3 | H-1α, H-1β | |
| 3 | 177.2 | | | | | | |
| 4 | 85.4 | | | | | | |
| 5 | 51.7 | 1.93 | 1H | m | 6, 7, 15 | H-6α, H-6β | H-6α, H-8, H-14 |
| 6 | 21.6 | 1.46 (α) | 1H | m | | H-5, H-7 | H-5 |
| | | 1.72 (β) | 1H | m | | H-5, H-7 | H-15, H-8' |
| 7 | 27.9 | 1.49 | 2H | m | | H-6α, H-6β | |
| 8 | 33.1 | 1.68 | 1H | m | 6, 7, 12 | H-7, H-12 | H-5, H-12 |
| 9 | 85.0 | | | | | | |
| 10 | 52.3 | | | | | | |
| 11 | 41.5 | 1.93 | 2H | s | 8, 9, 10, 12, 2', 6', 7' | | |
| 12 | 16.3 | 0.99 | 3H | d (*J* = 6.3 Hz) | 7, 8, 9 | H-8 | H-8, H-6' |
| 13 | 103.7 | 5.27 | 1H | s | 1, 9, 10, 2' | | H-1β, H-7' |
| 14 | 31.8 | 1.27 | 3H | s | 4, 5, 15 | | H-5 |
| 15 | 25.5 | 1.33 | 3H | s | 4, 5, 14 | | H-6β |
| 1' | 50.2 | | | | | | |
| 2' | 108.5 | | | | | | |
| 3' | 78.6 | | | | | | |
| 4' | 202.8 | | | | | | |
| 5' | 55.8 | 3.82 | 1H | d (*J* = 12.6 Hz) | 4', 6', 9', 10' | H-6' | H-7', H-10' |
| 6' | 36.8 | 2.26 | 1H | dq (*J* = 12.6, 6.3 Hz) | 1', 5', 7', 9', 10' | H-5', H-10' | H-12, H-8', H-10' |
| 7' | 16.4 | 1.18 | 3H | s | 11, 1', 2', 6' | | H-13, H-10' |
| 8' | 19.9 | 1.43 | 3H | s | 2', 3', 4' | | H-6β, H-6' |
| 9' | 169.7 | | | | | | |
| 10' | 14.0 | 0.98 | 3H | d (*J* = 6.3 Hz) | 1', 5', 6' | H-6' | H-5', H-6', H-7' |
| 11' | 52.1 | 3.75 | 3H | s | 9' | | |

$^{1}H$ NMR: 500 MHz, $^{13}C$ NMR: 125 MHz (in $CDCl_3$)

Supplementary Table 22. NMR data for fumigatonoid C (**26**).

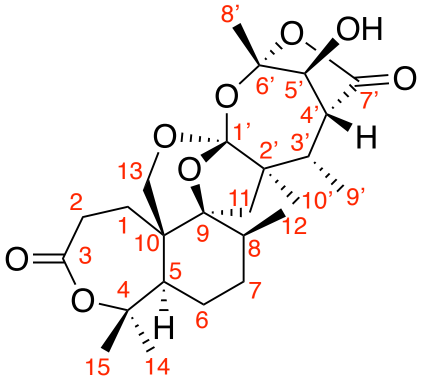


| | $^{13}C$ | $^{1}H$ | | | | | |
|---|---|---|---|---|---|---|---|
| position | δ (ppm) | δ (ppm) | intensitiy | multiplicity | HMBC correlation | COSY correlation | NOESY correlation |
| 1 | 31.0 | 1.58 (α) | 1H | ddt (*J* = 13.8, 6.3, 2.3 Hz) | 2, 3, 9, 13 | H-2α, H-2β, H-13β | H-2α, H-2β |
| | | 1.97 (β) | 1H | m | 13 | H-2α, H-2β | H-2β, H-13 |
| 2 | 32.7 | 2.78 (α) | 1H | td (*J* = 13.8, 6.3 Hz) | 1, 3 | H-1α, H-1β | H-1α, H-14 |
| | | 2.41 (β) | 1H | ddd (*J* = 13.8, 5.2, 2.3 Hz) | | H-1α, H-1β | H-1α, H-1β |
| 3 | 173.0 | | | | | | |
| 4 | 84.2 | | | | | | |
| 5 | 46.0 | 2.38 | 1H | dd (*J* = 13.2, 2.9 Hz) | 6, 9, 13, 15 | H-6α, H-6β | H-14 |
| 6 | 22.4 | 1.53 (α) | 1H | dt (*J* = 13.2, 3.5 Hz) | 5 | H-5, H-7α | H-7α |
| | | 2.01 (β) | 1H | m | 5 | H-5, H-7β | H-7β, H-13β, H-15 |
| 7 | 30.1* | 1.91 (α) | 1H | m | | H-6α, H-8 | H-6α |
| | | 1.37 (β) | 1H | m | 5, 12 | H-6β, H-8 | H-6β, H-12 |
| 8 | 30.0* | 2.00 | 1H | m | | H-7α, H-7β, H-12 | |
| 9 | 87.2 | | | | | | |
| 10 | 47.9 | | | | | | |
| 11 | 43.6 | 1.93 (α) | 1H | d (*J* = 13.8 Hz) | 8, 9, 10, 3', 12' | | H-10' |
| | | 2.14 (β) | 1H | d (*J* = 13.8 Hz) | 8, 9, 10, 1', 3', 12' | | H-12, H-3', H-9' |
| 12 | 17.8 | 1.05 | 3H | d (*J* = 6.9 Hz) | 7, 8, 9 | H-8 | H-7β, H-11β, H-1', 5'-OH |
| 13 | 64.8 | 3.50 (α) | 1H | d (*J* = 11.5 Hz) | 1, 9, 10, 1' | | H-1β, H-15 |
| | | 4.31 (β) | 1H | dd (*J* = 11.5, 2.3 Hz) | 1, 5, 1' | H-1α | H-6β, H-15 |
| 14 | 29.8* | 1.50 | 3H | s | 4, 5, 15 | | H-2α, H-5, |
| 15 | 28.0 | 1.31 | 3H | s | 3, 4, 5, 14 | | H-6β, H-13α, H-13β |
| 1' | 120.7 | | | | | | |
| 2' | 50.6 | | | | | | |
| 3' | 33.1 | 2.37 | 1H | m | 1', 2', 4', 5', 7', 9', 10' | H-4', H-9' | H-11β, H-12, H-4', H-9', 5'-OH |
| 4' | 48.4 | 2.53 | 1H | dd (*J* = 7.5, 2.3 Hz) | 2', 3', 5', 6', 7', 9' | H-3', H-5' | H-3', H-5', H-9' |
| 5' | 74.2 | 4.39 | 1H | dd (*J* = 12.6, 7.5 Hz) | 4' | H-4', 5'-OH | H-4', H-8' |
| 6' | 108.6 | | | | | | |
| 7' | 174.5 | | | | | | |
| 8' | 25.0 | 1.63 | 3H | s | 5', 6' | | H-5', 5'-OH |
| 9' | 16.3 | 1.22 | 3H | d (*J* = 6.9 Hz) | 2', 3', 4' | H-3' | H-11β, H-3', H-4' |
| 10' | 14.3 | 1.09 | 3H | s | 11, 1', 2', 3' | | H-11α |
| 5'-OH | | 4.68 | 1H | d (*J* = 12.6 Hz) | 5' | H-5' | H-12, H-3', H-8' |

$^{1}$H NMR: 500 MHz, $^{13}$C NMR: 125 MHz (in acetone-$d_6$)

Note: The carbons indicated by * were identified from HMQC spectrum.

## Supplementary Notes

### Supplementary Note 1. Absolute configurations of chemical compounds.

The absolute configurations of all the compounds were deduced based on that of novofumigatonin (**1**), which was established by both X-ray diffraction experiments and vibrational circular dichroism in combination with density functional calculations[1].

### Supplementary Note 2. Purification of novofumigatonin (1).

The extract from *A. novofumigatus* IBT 16806 cultivated on 200 YES agar plates was subjected to flash chromatography with Biotage® SNAP cartridge containing KP-C18-HS (120 g) and eluted stepwise using a water:methanol gradient. Fractions that contained **1** were further purified by reverse-phase preparative HPLC (55% aqueous acetonitrile, 5.0 mL $min^{-1}$) using Luna II C18 column (250 × 10 mm, 5 µm, Phenomenex) to yield 54.3 mg of a white amorphous solid. Fractions that contained **2** were further purified by reverse-phase preparative HPLC (65% aqueous acetonitrile, 5.0 mL $min^{-1}$) using Luna II C18 column (250 × 10 mm, 5 µm, Phenomenex) to yield 6.3 mg of a white amorphous solid.

$[\alpha]^{20}_{D}$ -30.8 (*c* 1.00, MeOH); HR-ESI-MS found *m/z* 461.2171 $[M + H]^{+}$ (calcd 461.2170 for $C_{25}H_{33}O_{8}$). The NMR data are in good agreement with the reported data[1].

### Supplementary Note 3. Purification of asnovolin A (2).

The extract from *A. novofumigatus* IBT 16806 cultivated on 200 YES agar plates was subjected to flash chromatography with Biotage® SNAP cartridge containing KP-C18-HS (120 g) and eluted stepwise using a water:methanol gradient. Fractions that contained **2** were further purified by reverse-phase preparative HPLC (65% aqueous acetonitrile, 5.0 mL $min^{-1}$) using Luna II C18 column (250 × 10 mm, 5 µm, Phenomenex) to yield 6.3 mg of a white amorphous solid.

$[\alpha]^{20}_{D}$ -114.6 (*c* 0.54, MeOH); HR-ESI-MS found *m/z* 447.2744 $[M + H]^{+}$ (calcd 447.2741 for $C_{26}H_{39}O_{6}$). The NMR data are in good agreement with the reported data[2].

### Supplementary Note 4. Purification conditions for farnesyl-DMOA (4).

The extract from *A. oryzae* NSAR1 with *andM* and *nvfB* cultivated in one liter of DPY was subjected to open column chromatography with Wakogel® Diol and eluted stepwise using a chloroform:methanol gradient (100:0 to 90:10). Fractions that contained **4** were further purified by reverse-phase preparative HPLC (85% aqueous acetonitrile containing 0.5% acetic acid, 4.0 mL $min^{-1}$) using COSMOSIL $5C_{18}$-AR-II column (10 i.d. x 250 mm; Nacalai Tesque, Inc.) to yield 49.52 mg of a yellowish oil.

$[\alpha]^{27}_{D}$ +2.9 (*c* 1.00, MeOH); HR-ESI-MS found *m/z* 401.2680 $[M + H]^{+}$ (calcd 401.2686 for $C_{25}H_{37}O_{4}$). The NMR data are in good agreement with the reported data[3].

**Supplementary Note 5. Purification of asnovolin G (18).**

The extract from *A. novofumigatus* mutant (*pyrG*$^-$, *ligD*Δ, *nvfF*Δ*::AfpyrG*) cultivated on 100 YES agar plates was subjected to flash chromatography with Biotage® SNAP cartridge containing ISOLUTE® DIOL (100 g) and eluted stepwise using a dichloromethane:ethyl acetate gradient. Fractions that contained **18** were further purified by reverse-phase preparative HPLC (45% aqueous acetonitrile, 5.0 mL min$^{-1}$) using Luna II C18 column (250 × 10 mm, 5 μm, Phenomenex) to yield 2.8 mg of a white amorphous solid.

$[\alpha]^{20}_{D}$ -38.5 (*c* 0.24, MeOH); HR-ESI-MS found *m/z* 465.2485 [M + H]$^+$ (calcd 465.2483 for $C_{25}H_{37}O_8$). The NMR data are in good agreement with the reported data[2].

**Supplementary Note 6. Purification of asnovolin B (23).**

The extract from *A. oryzae* NSAR1 with *andM*, *nvfB*, *nvfK*, *nvfL*, *nvfC*, *nvfH*, *nvfJ*, *nvfM*, *nvfI*, and *nvfD* cultivated in four liter of DPY was subjected to open column chromatography with Silica Gel 60 N and eluted stepwise using a hexane:chloroform (100:0 to 50:50) and then chloroform:methanol gradient (100:0 to 95:5). Fractions that contained **23** were further purified by reverse-phase preparative HPLC (60% aqueous acetonitrile, 3.0 mL min$^{-1}$) using YMC-Triart C18 column (10 i.d. x 250 mm; YMC CO., LTD.) to yield 7.30 mg of a white amorphous solid.

$[\alpha]^{25}_{D}$ -34.1 (*c* 0.31, $CHCl_3$); HR-ESI-MS found *m/z* 463.2686 [M + H]$^+$ (calcd 463.2690 for $C_{26}H_{39}O_7$). The NMR data are in good agreement with the reported data[2].

**Supplementary Note 7. Purification and structural determination of asnovolin I (5).**

The extract from *A. novofumigatus* mutant (*pyrG*$^-$, *ligD*Δ, *nvfJ*Δ*::AfpyrG*) cultivated on 100 YES agar plates was subjected to flash chromatography with Biotage® SNAP cartridge containing ISOLUTE® DIOL (100 g) and eluted stepwise using a dichloromethane:ethyl acetate gradient. Fractions that contained **5** were further purified by reverse-phase preparative HPLC (50% aqueous acetonitrile containing 0.5% acetic acid, 5.0 mL min$^{-1}$) using Kinetex C18 column (250 × 10 mm, 5 μm, Phenomenex) to yield 8.7 mg of a white amorphous solid.

The molecular formula of **5** was established as $C_{25}H_{34}O_6$ by HR-MS, indicating nine degrees of unsaturation. The $^{13}C$ NMR spectrum revealed 25 signals consisting of seven methyls, five methylenes, two methines, two *sp*$^3$ quaternary carbons, two oxygenated tertiary carbons (C-4, and C-9), four olefinic carbons (C-2', C-3', C-5', and C-6'), two carboxylate or ester groups (C-3 and C-9'), and one ketone (C-4'), which indicated the tetracyclic nature of **5**.

Interpretation of the $^1H$-$^1H$ DQF-COSY spectrum then revealed the spin systems of H-1/H-2, H-5/H-6, and H-8/H-12. Additionally, HMBC correlations from the methyl signals were observed as follows: $H_3$-12 (δ 0.88) to C-7, C-8, and C-9; $H_3$-13 (δ 1.41) to C-1, C-5, C-9, and C-10; $H_3$-14 (δ 1.54) to C-4, C-5, and C-15; $H_3$-15 (δ 1.47) to C-4, C-5, and C-14; $H_3$-7' (δ 1.65) to C-11, C-1', C-2', and C-6'; $H_3$-8' (δ 1.83) to C-2', C-3', and

C-4'; and $H_3$-10' (δ 2.33) to C-1', C-5', and C-6'. These cross peaks confirmed the connections of C-4 to C-5, C-14, and C-15, C-8 to C-7 and C-9, C-10 to C-1, C-5, C-9 and C-13, C-1' to C-11, C-2', C-6', and C-7', C-3' to C-2', C-4', and C-8', and C-6' to C-5' and C-10'. Furthermore, the HMBC correlations of H-1α (δ 1.92) to C-3, H-5 (δ 2.20) to C-7, and $H_2$-11 (δ 2.30) to C-8 revealed the connections of C-2 to C-3, C-6 to C-7, and C-9 to C-11, respectively. Then, the connection of C-3 to C-4 *via* one oxygen atom to form the seven-membered lactone ring was established based on the chemical shift of C-4 (δ 87.3). The connection of C-9 to C-2' was deduced based on the observation that novofumigatonin (**1**) and their analogues known to date all possess the tetrahydrofuran ring. Finally, the connections of C-5' to C-4' and C-9' were established as this is the only one possibility to connect the remaining carbon atoms.

The relative configuration of **5** at C-5, C-8, and C-10 was determined by NOSEY correlations: H-2β and H-13/H-15, H-5 and H-2α/H-14, and H-12 and H-13. The stereochemistry at C-9 and C-1' was deduced to be the same as that of asnovolin A (**2**).

$[\alpha]^{20}_D$ +8.4 (*c* 0.28, MeOH); HR-ESI-MS found *m/z* 431.2425 $[M + H]^+$ (calcd 431.2415 for $C_{25}H_{35}O_6$). Note that the structure of **5** was determined using NMR spectra containing some impurity.

## Supplementary Note 8. Purification and structural determination of compound 6.

The extract from *A. novofumigatus* mutant (*pyrG*$^-$, *ligD*Δ, *nvfL*Δ*::AfpyrG*) cultivated on 100 YES agar plates was subjected to flash chromatography with Biotage® SNAP cartridge containing ISOLUTE® DIOL (100 g) and eluted stepwise using a dichloromethane:ethyl acetate gradient. Fractions that contained **6** were further purified by reverse-phase preparative HPLC (45% aqueous acetonitrile containing 0.5% acetic acid, 5.0 mL $min^{-1}$) using Kinetex C18 column (250 × 10 mm, 5 µm, Phenomenex) to yield 7.1 mg of a yellowish oil.

The molecular formula of **6** was established as $C_{25}H_{38}O_6$ by HR-MS. The structure of **6** was determined by utilizing the NMR data for dihydroxyfarnesyl-DMOA obtained from the biosynthetic study on terretonin[3]. Since **6** is predicted to be the C-10' epimer of dihydroxyfarnesyl-DMOA from the terretonin pathway due to the different stereochemistry of the epoxide whose hydrolysis gave these diastereomers, the NMR data of **6** did not completely match with those for dihydroxyfarnesyl-DMOA for the terretonin biosynthesis[3]. Yet, these NMR data are highly similar, and therefore the structure of **6** was readily determined as shown in the next page.

$[\alpha]^{20}_D$ +13.1 (*c* 0.48, MeOH); HR-ESI-MS found *m/z* 457.2570 $[M + Na]^+$ (calcd 457.2558 for $C_{25}H_{38}O_6Na$).

## Supplementary Note 9. Purification and structural determination of asnovolin H (8).

The extract from *A. oryzae* NSAR1 with *andM*, *nvfB*, *nvfK*, *nvfL*, and *nvfC* cultivated in one liter of DPY was subjected to open column chromatography with Silica Gel 60 N and eluted stepwise using a chloroform:methanol gradient (100:0 to 90:10). Fractions that contained **8** were further purified by reverse-phase preparative HPLC (70% aqueous acetonitrile containing 0.01% trifluoroacetic acid, 4.0 mL

min$^{-1}$) using COSMOSIL 5C$_{18}$-AR-II column (10 i.d. x 250 mm; Nacalai Tesque, Inc.) to yield 2.20 mg of a white amorphous solid.

The molecular formula of **8** was established as $C_{25}H_{36}O_5$ by HR-MS, indicating eight degrees of unsaturation. The $^{13}$C NMR spectrum revealed 25 signals consisting of seven methyls, five methylenes, three methines including one oxymethine (C-3), three *sp*$^3$ quaternary carbons, one oxygenated tertiary carbons (C-9), four olefinic carbons (C-2', C-3', C-5', and C-6'), one carboxylate (C-9'), and one ketone (C-4'), which indicated the tetracyclic nature of **8**.

Interpretation of the $^1$H-$^1$H COSY spectrum then revealed the spin systems of H-1/H-2/H-3, H-5/H-6, and H-8/H-12. Additionally, HMBC correlations from the methyl signals were observed as follows: $H_3$-12 (δ 1.12) to C-7, C-8, and C-9; $H_3$-13 (δ 1.24) to C-1, C-5, C-9, and C-10; $H_3$-14 (δ 1.03) to C-4, C-5, and C-15; $H_3$-15 (δ 0.86) to C-4, C-5, and C-14; $H_3$-7' (δ 1.58) to C-11, C-1', C-2', and C-6'; $H_3$-8' (δ 1.87) to C-2', C-3', and C-4'; and $H_3$-10' (δ 2.60) to C-1', C-5', and C-6'. These cross peaks confirmed the connections of C-4 to C-3, C-5, C-14, and C-15, C-8 to C-7 and C-9, C-10 to C-1, C-5, C-9 and C-13, C-1' to C-11, C-2', C-6', and C-7', C-3' to C-2', C-4', and C-8', and C-6' to C-5' and C-10'. Furthermore, the HMBC correlations of H-5 (δ 0.85) to C-7 and $H_2$-11 (δ 2.37 and 1.89) to C-8 revealed the connections of C-6 to C-7 and C-9 to C-11, respectively. The connections of C-9 to C-2' and C-5' to C-4' and C-9' were deduced in a similar way as described for the structural characterization of **5**.

The relative configuration of **8** was determined by NOSEY correlations: H-5 and H-3/H-11α/H-14, H-11α and H-7', and H-13 and H-12/H-15.

$[\alpha]^{25}_D$ -63.5 (*c* 0.15, $CHCl_3$); HR-ESI-MS found *m/z* 417.2631 $[M + H]^+$ (calcd 417.2636 for $C_{25}H_{37}O_5$).

**Supplementary Note 10. Purification and structural determination of chermesin D (10).**

The extract from *A. oryzae* NSAR1 with *andM*, *nvfB*, *nvfK*, *nvfL*, and *nvfC* cultivated in one liter of DPY was subjected to open column chromatography with Silica Gel 60 N and eluted stepwise using a chloroform:methanol gradient (100:0 to 90:10). Fractions that contained **10** were further purified by reverse-phase preparative HPLC (70% aqueous acetonitrile containing 0.01% trifluoroacetic acid, 4.0 mL min$^{-1}$) using COSMOSIL 5C$_{18}$-AR-II column (10 i.d. x 250 mm; Nacalai Tesque, Inc.) to yield 1.37 mg of a white amorphous solid.

The molecular formula of **10** was established as $C_{25}H_{34}O_5$ by HR-MS, which suggested that **10** is a dehydrogenated form of **8**. Accordingly, in the $^1$H/$^{13}$C NMR spectra of **10**, the signals for the oxymethine at C-3 of **8** disappeared, while a new signal for a ketone (δ 215.0) appeared in the $^{13}$C NMR spectrum of **10**. Thus, it was indicated that **10** is the oxidized form of **8** with the ketone functionality at C-3, which was confirmed by the further analysis of 2D NMR spectra of **10**.

$[\alpha]^{25}_D$ -72.8 (*c* 0.08, $CHCl_3$); HR-ESI-MS found *m/z* 415.2473 $[M + H]^+$ (calcd 415.2479 for $C_{25}H_{35}O_5$). The NMR data of **10** was reportedly recorded in DMSO-$d_6$[4], but **10** was unstable in DMSO and we were not able

to obtain $^{13}C$ NMR spectrum in DMSO-$d_6$. Therefore, the NMR data was obtained in $CDCl_3$.

**Supplementary Note 11. Purification and structural determination of asnovolin J (11).**

The extract from *A. novofumigatus* mutant (*pyrG*$^-$, *ligD*Δ, *nvfH*Δ*::AfpyrG*) cultivated on 100 YES agar plates was subjected to flash chromatography with Biotage® SNAP cartridge containing ISOLUTE® DIOL (100 g) and eluted stepwise using a dichloromethane:ethyl acetate gradient. Fractions that contained **11** were further purified by reverse-phase preparative HPLC (70% aqueous acetonitrile, 5.0 mL min$^{-1}$) using Luna II C18 column (250 × 10 mm, 5 μm, Phenomenex) to yield 4.1 mg of a white amorphous solid.

The molecular formula of **11** was established as $C_{26}H_{38}O_5$ by HR-MS. The NMR spectra of **11** are similar to those of asnovolin A (**2**), and especially, the signals for the DMOA-derived moiety (C-1' to C-11') are almost identical. The major spectral differences between **11** and **2** is that the $^{13}C$ NMR spectrum of **11** lacks signals for the ester carbonyl at C-3 and the oxygenated tertiary carbons at C-4. Alternatively, the $^{13}C$ NMR spectrum of **11** has a new signal for a ketone (δ 215.5), to which HMBC correlations from $H_3$-14 (δ 1.10) and $H_3$-15 (δ 1.10) were observed. This indicates that **11** has a cyclohexanone ring instead of the seven-membered lactone ring of **2**, which was confirmed by further analysis of 2D NMR spectra of **11**.

$[\alpha]^{20}_D$ -205.2 (*c* 0.24, MeOH); HR-ESI-MS found *m/z* 431.2797 $[M + H]^+$ (calcd 431.2792 for $C_{26}H_{39}O_5$).

**Supplementary Note 12. Purification and structural determination of asnovolin K (12).**

The extract from *A. novofumigatus* mutant (*pyrG*$^-$, *ligD*Δ, *nvfM*Δ*::AfpyrG*) cultivated on 100 YES agar plates was subjected to flash chromatography with Biotage® SNAP cartridge containing KP-C18-HS (60 g) and eluted stepwise using a water:methanol gradient. Fractions that contained **12** were further purified by reverse-phase preparative HPLC (55% aqueous acetonitrile containing 0.5% acetic acid, 5.0 mL min$^{-1}$) using Luna II C18 column (250 × 10 mm, 5 μm, Phenomenex) to yield 1.6 mg of a white amorphous solid.

The molecular formula of **12** was established as $C_{26}H_{36}O_6$ by HR-MS. The NMR spectra of **12** are similar to those of asnovolin I (**5**), but the NMR spectra revealed the presence of an additional *O*-methyl group (C-11; $\delta_H/\delta_C$ 3.85/52.3). The HMBC correlation of $H_3$-11' to C-9' (δ 167.6) established the existence of a methyl ester, and therefore it was indicated that **12** is the methyl ester derivative of **5**, which was confirmed by further analysis of 2D NMR spectra of **12**.

$[\alpha]^{20}_D$ +48.8 (*c* 0.13, MeOH); HR-ESI-MS found *m/z* 445.2588 $[M + H]^+$ (calcd 445.2585 for $C_{26}H_{37}O_6$).

**Supplementary Note 13. Purification and structural determination of fumigatonoid A (14).**

The extract from *A. novofumigatus* mutant (*pyrG*$^-$, *ligD*Δ, *nvfD-G*Δ, *e-anaPS*Δ*::AfpyrG*) cultivated on 100 YES agar plates was subjected to flash chromatography with Biotage® SNAP cartridge containing ISOLUTE® DIOL (50 g) and eluted stepwise using a dichloromethane:ethyl acetate gradient. Fractions that contained **14** were further purified by reverse-phase preparative HPLC (50% aqueous acetonitrile, 5.0 mL min$^{-1}$) using

Luna II C18 column (250 × 10 mm, 5 μm, Phenomenex) to yield 4.3 mg of a white amorphous solid.

The molecular formula of **14** was established as $C_{26}H_{38}O_9$ by HR-MS, indicating eight degrees of unsaturation. The structure of **14** was initially investigated using NMR spectra recorded in $CDCl_3$. The $^{13}$C NMR spectrum revealed 26 signals, consisting of seven methyls (including one methoxy; C-11'), six methylenes including one oxymethylene (C-13), four methines, two *sp*$^3$ quaternary carbons, three oxygenated tertiary carbons (C-4, C-9, and C-3'), one acetal or hemiacetal (C-2'), two esters (C-3 and C-9'), and one ketone (C-4'). Meanwhile, no olefinic carbon was observed in the $^{13}$C NMR spectrum, thus indicating the pentacyclic nature of **14**.

Interpretation of the $^1$H-$^1$H DQF-COSY spectrum then revealed the spin systems of H-1/H-2, H-5/H-6/H-7/H-8/H-12, and H-5'/H-6'/H-10' (Supplementary Fig. 8b). Additionally, HMBC correlations from the methyl signals were observed as follows: $H_3$-12 (δ 0.82) to C-7, C-8, and C-9; $H_3$-14 (δ 1.39) to C-4, C-5, and C-15; $H_3$-15 (δ 1.18) to C-3, C-4, C-5, and C-14; $H_3$-7' (δ 1.20) to C-11, C-1', C-2', and C-6'; $H_3$-8' (δ 1.23) to C-2', C-3', and C-4'; $H_3$-10' (δ 0.90) to C-1', C-5', and C-6'; and $H_3$-11' (δ 3.63) to C-9'. These cross peaks confirmed the connections of C-4 to C-5, C-14, and C-15 as well as to C-3 *via* one oxygen atom, C-8 to C-9, C-1' to C-11, C-2', C-6', and C-7', C-3' to C-2', C-4', and C-8', and C-11' to C-9' *via* one oxygen atom (Supplementary Fig. 8b). Furthermore, the HMBC correlations of $H_2$-11 (δ 2.31 and 2.08) to C-8, $H_2$-13 (δ 4.80 and 4.49) to C-1, C-5, C-9, and C-10, and H-6' (δ 2.51) to C-4' and C-9' revealed the connections of C-10 to C-1, C-5, C-9, and C-13, C-9 to C-11, and C-5' to C-4' and C-9' (Supplementary Fig. 8b). Finally, another HMBC correlation of $H_2$-2 (δ 2.56) to C-3 established the connection of C-2 to C-3, which allowed connecting all the carbon atoms in the structure. The presence of the C-3' alcohol was then confirmed by the NMR spectra recorded in DMSO-$d_6$, since a signal for a hydroxyl proton (δ 6.00) was clearly observed in the $^1$H NMR spectrum and the signal correlates with C-2', C-3', C-4', and C-8' in the HMBC spectrum.

As discussed above, six out of nine oxygen atoms in **14** have been successfully placed in the structure, but the placement of the other three oxygens, which should be bound to C-9, C-13, and C-2', still remained elusive (Supplementary Fig. 9a). Given the number of remaining oxygen atoms to be placed, this could be accounted for by one ether and one endoperoxide linkages to connect all the atoms, which leaves three possibilities as the planar structure of **14**. Considering that another endoperoxide **15** has the peroxide linkage between C-13 and C-2' as described in the main text and that novofumigatonin (**1**) and their analogues known to date all possess the tetrahydrofuran C-ring, we conclude that **14** also has an endoperoxide linkage between C-13 and C-2', which established the planar structure of **14**.

The relative configuration of **14** was mainly determined by NOESY correlations obtained in $CDCl_3$ (Supplementary Fig. 8c). For the relative configuration of the A- and B-rings, the 5*R**, 8*S**, 10*S** configuration was established based on the correlations of H-5 (δ 2.16) and H-6α/H-8 and H-13β (δ 4.80) and H-6β. For the relative configuration of the C- and D-rings, the 1'*R**, 5'*S**, 6'*S** configuration was deduced based on the correlations of H-11β (δ 2.08) and H-6' and H-7' (δ 1.30) and H-11α/H-5'/H-10'. The *trans* configuration of the hydrogens at C-5' and C-6' is also supported by the large coupling constant ($J$ = 13.2 Hz)

of these two protons in the $^{1}$H NMR spectrum. The 3'$R$* configuration was determined by the correlation of H-7' (δ 1.30) and 3'-OH observed in the NOESY spectrum obtained in DMSO-$d_6$. The stereochemistry of the spiro center at C-9 was established as $S$* based on the correlations of H-11α (δ 2.31) and H-1α/H-1β. Finally, the 2'$S$* configuration was deduced, since the 2'$R$* configuration is highly unlikely due to the orthogonal positioning of the B- and C- rings.

$[\alpha]^{20}_{D}$ +138.8 ($c$ 0.19, $CHCl_3$); HR-ESI-MS found $m/z$ 495.2592 $[M + H]^+$ (calcd 495.2589 for $C_{26}H_{39}O_9$).

### Supplementary Note 14. Purification and structural determination of fumigatonoid B (15).

The extract from *A. novofumigatus* mutant (*pyrG*$^-$, *ligD*Δ, *nvfE*Δ*::AfpyrG*) cultivated on 100 YES agar plates was subjected to flash chromatography with Biotage® SNAP cartridge containing ISOLUTE® DIOL (100 g) and eluted stepwise using a dichloromethane:ethyl acetate gradient. Fractions that contained **15** were further purified by reverse-phase preparative HPLC (50% aqueous acetonitrile, 5.0 mL min$^{-1}$) using Luna II C18 column (250 × 10 mm, 5 µm, Phenomenex) to yield 7.5 mg of a white amorphous solid.

The molecular formula of **15** was established as $C_{25}H_{36}O_8$ by HR-MS, indicating eight degrees of unsaturation. The $^{13}$C NMR spectrum revealed 25 signals including two esters, which indicated the hexacyclic nature of **15**. The $^{1}$H and $^{13}$C NMR spectra of **15** were somewhat similar to those of **14** but revealed the disappearance of signals for the C-11' methyl group and C-4' ketone, and alternatively one new oxymethine signal was observed at $\delta_H/\delta_C$ 4.06/77.4. In a similar manner described for **14**, detailed interpretation of 2D NMR spectra enabled the connection of all the 25 carbon atoms and confirmed the presence of the seven-membered lactone A-ring as well as the hydroxyl group attached to C-4' (Supplementary Fig. 9b). At this point, four out of eight oxygen atoms were successfully placed, and four more oxygens, bound to C-9, C-13, C-2', C-3', and C-9', needed to be correctly set in the structure (Supplementary Fig. 9a). Given the hexacyclic nature of **15** and the number of oxygens in the structure, this altogether indicated the presence of one additional lactone, one ether, and one endoperoxide. As described in the main text, the position of the endoperoxide linkage was determined to be between C-13 and C-2', which leaves three possibilities as the planar structure of **15**. Considering the structures of novofumigatonin (**1**) and **14**, it would be most likely that **15** possesses the tetrahydrofuran C-ring and the five-membered lactone F-ring as seen in **1**, and the planar structure of **15** was thus established.

The relative configuration of **15** was mainly determined by NOESY correlations (Supplementary Fig. 9c). The 5$R$*, 10$S$* configuration was established based on the correlations of H-5 (δ 2.21) and H-6α/H-14 and H-13β (δ 4.79) and H-6β/H-15, and the 9$S$*, 1'$R$*, 6'$S$* configuration was determined based on the correlations of H-11α (δ 2.22) and H-1α/H-1β/H-7' and H-11β (δ 2.16) and H-6'. The correlation of the C-4' hydroxyl proton (δ 4.17) and H-7β/H-12/H-6' then confirmed the 8$S$*, 4'$S$* configuration. The stereocheimstry at C-5' was deduced to be $R$* based on the small coupling constant between C-5' and C-6' protons ($J$ = 1.5 Hz), which indicates that the orientation of H-5' is different from that of **14**. Considering that the methyl group at C-3' and H-5' should be *cis* oriented to make it possible to form the five-membered lactone ring, the 3'$R$* configuration

was deduced. Finally, the 2'*S** configuration was deduced as discussed above for **14**, establishing the relative configuration of **15**.

$[\alpha]^{20}_{D}$ +157.9 (*c* 0.37, $CHCl_3$); HR-ESI-MS found *m/z* 465.2485 $[M + H]^+$ (calcd 465.2483 for $C_{25}H_{37}O_8$).

**Supplementary Note 15. Structural determination of compound 16.**

The molecular formula of **16** was established as $C_{25}H_{38}O_8$ by HR-MS, which is consistent with the fact that **16** is a hydrogenated product of **15**. The positions of two resulting hydroxyl groups from the hydrogenation were determined by HMBC correlations of one hydroxyl proton (δ 4.12) to C-4, C-5, C-14, and C-15 and another hydroxyl proton (δ 5.19) to C-1' and C-2'. Thus, **16** has hydroxyl groups at C-4 and C-2' as well as at C-4', which indicates that the seven-membered lactone ring of **15** is opened by the translactonization caused by the C-13 hydroxyl group generated by the hydrogenation of the peroxide of **15**. This predicted structure was then confirmed by the further analysis of 2D NMR spectra of **16**.

**Supplementary Note 16. Purification and structural determination of novofumigatonol (19).**

The extract from *A. novofumigatus* mutant (*pyrG*⁻, *ligD*Δ, *nvfG*Δ*::AfpyrG*) cultivated on 100 YES agar plates was subjected to flash chromatography with Biotage® SNAP cartridge containing ISOLUTE® DIOL (100 g) and eluted stepwise using a dichloromethane:ethyl acetate gradient. Fractions that contained **19** were further purified by reverse-phase preparative HPLC (45% aqueous acetonitrile, 5.0 mL $min^{-1}$) using Luna II C18 column (250 × 10 mm, 5 µm, Phenomenex) to yield 1.5 mg of a white amorphous solid.

The molecular formula of **19** was established as $C_{25}H_{32}O_9$ by HR-MS. The NMR spectra of **19** are highly similar to those of novofumigatonin (**1**), except that a signal for the oxymethine at C-5' of **1** is missing in **19**. Alternatively, the $^{13}C$ NMR spectrum of **19** revealed the presence of an additional signal for an acetal or hemiacetal (δ 105.4). Given that **19** has one additional oxygen atom compared with **1**, it was indicated that **19** possesses a hydroxyl group at C-5', which was confirmed by further analysis of 2D NMR spectra of **19**.

$[\alpha]^{20}_{D}$ -12.0 (*c* 0.18, MeOH); HR-ESI-MS found *m/z* 477.2215 $[M + H]^+$ (calcd 477.2219 for $C_{25}H_{33}O_9$).

**Supplementary Note 17. Purification and structural determination of compound 21.**

The purified NvfI (final conc. 9.6 µM) was added to 50 mL of reaction buffer (50 mM Tris-HCl buffer pH 7.5, 0.1 mM $FeSO_4$, 2.5 mM α-ketoglutarate, 4 mM ascorbate) and incubated with 250 µM fumigatonoid A (**14**) at 30 °C for 3 hrs. The reaction mixture was extracted with ethyl acetate and purified by reverse-phase preparative HPLC (50% aqueous acetonitrile, 4.0 mL $min^{-1}$) using COSMOSIL $5C_{18}$-AR-II column (10 i.d. x 250 mm; Nacalai Tesque, Inc.) to yield 2.96 mg of white amorphous solid.

The molecular formula of **21** was established as $C_{26}H_{38}O_9$ by HR-MS. The NMR spectra of **21** are similar to those of fumigatonoid A (**14**), but the signals for the oxymethylene at C-13 are missing in **21**. Alternatively, the $^{1}$NMR spectrum of **21** revealed the presence of a hemiacetal ($\delta_H/\delta_C$ 5.27/103.7) at C-13. The HMBC

correlation of the hemiacetal proton to C-2' established the connection of C-13 and C-2' *via* one oxygen atom. This indicated that **21** is the isomer of **14** with a hemiacetal linkage between C-13 and C-2', which was confirmed by further analysis of 2D NMR spectra of **21**.

$[\alpha]^{25}_{D}$ +36.3 (*c* 0.24, $CHCl_3$); HR-ESI-MS found *m/z* 495.2594 $[M + H]^+$ (calcd 495.2589 for $C_{26}H_{39}O_9$).

## Supplementary Note 18. Purification and structural determination of fumigatonoid C (26).

The purified NvfE (final conc. 2.6 μM) was added to 100 mL of reaction buffer (50 mM Tris-HCl buffer pH 7.5, 0.1 mM $FeSO_4$, 4 mM ascorbate) and incubated with 50 μM fumigatonoid B (**15**) at 30 °C for 1.5 hrs. The reaction mixture was extracted with ethyl acetate and purified by reverse-phase preparative HPLC (55% aqueous acetonitrile, 4.0 mL $min^{-1}$) using COSMOSIL $5C_{18}$-AR-II column (10 i.d. x 250 mm; Nacalai Tesque, Inc.) to yield 1.60 mg of white amorphous solid.

The molecular formula of **26** was established as $C_{26}H_{36}O_8$ by HR-MS. The $^{13}C$ NMR spectra of **26** were somewhat similar to those of **15** but revealed the disappearance of a signal for the oxygenated tertiary carbon at C-3' of **15**, and alternatively one new orthoester signal was observed at 120.7 ppm (C-1'). Additionally, the signals for the C-13 oxymethylene ($\delta_H/\delta_C$ 4.31, 3.50/64.8) were observed at higher magnetic field compared with those of **15**, suggesting a structural change around this position. The HMBC correlation from $H_2$-13 to C-1' then established the connection of C-13 and C-1' *via* one oxygen atom. Furthermore, HMBC correlations from $H_3$-8' (δ 1.63) were only observed at two carbons, C-6' acetal (δ 108.6) and C-7' oxymethine (δ 74.2), which indicated the insertion of an oxygen atom between C-1' and C-6' to generate the C-1' orthoester and the C-6' acetal. Collectively, it was proposed that **26** has the same backbone structure as that of fumigatonin[5], which was confirmed by further analysis of 2D NMR spectra of **26**.

$[\alpha]^{25}_{D}$ +35.7 (*c* 0.14, acetone); HR-ESI-MS found *m/z* 465.2479 $[M + H]^+$ (calcd 465.2483 for $C_{25}H_{37}O_8$).

## Supplementary References


1 Rank, C. *et al.* Novofumigatonin, a new orthoester meroterpenoid from *Aspergillus novofumigatus*. *Org. Lett.* **10**, 401-404 (2008).

2 Ishikawa, K. *et al.* Asnovolins A-G, spiromeroterpenoids isolated from the fungus *Aspergillus novofumigatus*, and suppression of fibronectin expression by asnovolin E. *J. Nat. Prod.* **79**, 2167-2174 (2016).

3 Itoh, T. *et al.* Identification of a key prenyltransferase involved in biosynthesis of the most abundant fungal meroterpenoids derived from 3,5-dimethylorsellinic acid. *ChemBioChem* **13**, 1132-1135 (2012).

4 Liu, H. *et al.* Chermesins A-D: Meroterpenoids with a drimane-type spirosesquiterpene skeleton from the marine algal-derived endophytic fungus *Penicillium chermesinum* EN-480. *J. Nat. Prod.* **79**, 806-811 (2016).

5 Okuyama, E., Yamazaki, M. & Katsube, Y. Fumigatonin, a new meroterpenoid from *Aspergillus fumigatus*. *Tetrahedron Lett.* **25**, 3233-3234 (1984).
