# Supplementary material for: Novofumigatonin biosynthesis involves a non-heme iron-dependent endoperoxide isomerase for orthoester formation
Source: Nat Commun. 2018 Jul 3;9:2587. doi: 10.1038/s41467-018-04983-2 (PMC6030086; doi:10.1038/s41467-018-04983-2)
Supplement: Supplementary file 3 — Description of Additional Supplementary Files [file 41467_2018_4983_MOESM3_ESM.pdf]

Description of Additional Supplementary Files

File Name: Supplementary Data 1
Description: Primers used in this study.

File Name: Supplementary Data 2
Description: Plasmids constructed in this study.
